# Exploration of binary protein–protein interactions between tick-borne flaviviruses and *Ixodes ricinus*

**DOI:** 10.1186/s13071-021-04651-3

**Published:** 2021-03-06

**Authors:** Manon Lemasson, Grégory Caignard, Yves Unterfinger, Houssam Attoui, Lesley Bell-Sakyi, Edouard Hirchaud, Sara Moutailler, Nicholas Johnson, Damien Vitour, Jennifer Richardson, Sandrine A. Lacour

**Affiliations:** 1 UMR 1161 Virologie Laboratoire de Santé Animale, ANSES, INRAE, Ecole Nationale Vétérinaire d’Alfort, Paris-Est Sup, Maisons-Alfort, France; 2 Department of Infection Biology and Microbiomes, Institute of Infection, Veterinary and Ecological Sciences, University of Liverpool, Liverpool, UK; 3 Viral Genetic and Biosecurity Unit, Ploufragan-Plouzané-Niort Laboratory, ANSES, Ploufragan, France; 4 UMR BIPAR, Laboratoire de Santé Animale, ANSES, INRAE, Ecole Nationale Vétérinaire d’Alfort, Paris-Est Sup, Maisons-Alfort, France; 5 Animal and Plant Health Agency (APHA), Addlestone, Surrey, UK

## Abstract

**Background:**

Louping ill virus (LIV) and tick-borne encephalitis virus (TBEV) are tick-borne flaviviruses that are both transmitted by the major European tick, *Ixodes ricinus*. Despite the importance of *I. ricinus* as an arthropod vector, its capacity to acquire and subsequently transmit viruses, known as vector competence, is poorly understood. At the molecular scale, vector competence is governed in part by binary interactions established between viral and cellular proteins within infected tick cells.

**Methods:**

To investigate virus-vector protein–protein interactions (PPIs), the entire set of open reading frames for LIV and TBEV was screened against an *I. ricinus* cDNA library established from three embryonic tick cell lines using yeast two-hybrid methodology (Y2H). PPIs revealed for each viral bait were retested in yeast by applying a gap repair (GR) strategy, and notably against the cognate protein of both viruses, to determine whether the PPIs were specific for a single virus or common to both. The interacting tick proteins were identified by automatic BLASTX, and in silico analyses were performed to expose the biological processes targeted by LIV and TBEV.

**Results:**

For each virus, we identified 24 different PPIs involving six viral proteins and 22 unique tick proteins, with all PPIs being common to both viruses. According to our data, several viral proteins (pM, M, NS2A, NS4A, 2K and NS5) target multiple tick protein modules implicated in critical biological pathways. Of note, the NS5 and pM viral proteins establish PPI with several tumor necrosis factor (TNF) receptor-associated factor (TRAF) proteins, which are essential adaptor proteins at the nexus of multiple signal transduction pathways.

**Conclusion:**

We provide the first description of the TBEV/LIV-*I. ricinus* PPI network, and indeed of any PPI network involving a tick-borne virus and its tick vector. While further investigation will be needed to elucidate the role of each tick protein in the replication cycle of tick-borne flaviviruses, our study provides a foundation for understanding the vector competence of *I. ricinus* at the molecular level. Indeed, certain PPIs may represent molecular determinants of vector competence of *I. ricinus* for TBEV and LIV, and potentially for other tick-borne flaviviruses.
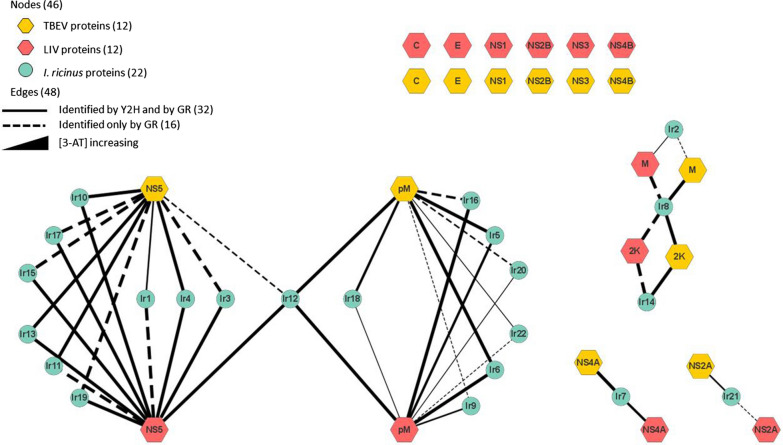

**Supplementary Information:**

The online version contains supplementary material available at 10.1186/s13071-021-04651-3.

## Background

Ticks are obligate hematophagous arthropods and vectors of a broad diversity of pathogens that affect humans, livestock and wildlife throughout the world. Ticks transmit parasites, bacteria and viruses, of which the last-mentioned belong to diverse viral families [1]. In Europe, two of the major tick-borne viruses, tick-borne encephalitis virus (TBEV) and louping ill virus (LIV), are members of the genus *Flavivirus* of the family *Flaviviridae* [2]. Both viruses are principally transmitted by the major tick species of northern Europe, *Ixodes ricinus* [3], which is the most significant arthropod vector in Europe and responsible for the transmission of multiple pathogens of medical and veterinary importance.

Despite high genetic proximity (95% at the amino acid level, data not shown), TBEV and LIV are mainly responsible for clinical disease in humans and sheep, respectively. While human infections by TBEV are mostly asymptomatic (70–95% of cases), between 10,000 and 15,000 cases of tick-borne encephalitis (TBE) are reported every year in Eurasia [4]. Clinical manifestations are diverse, but can include moderate fever, subacute encephalitis with complete or partial recovery and, in very rare cases, fatal encephalitis [5]. While humans generally acquire TBEV following a tick bite, the virus may also be transmitted via milk or dairy products from domestic animals such as goats, sheep and cattle [6]. LIV has predominantly been reported from the United Kingdom, and having been identified almost 100 years ago, was the first tick-borne virus to be discovered [7]. LIV causes an encephalitic disease in sheep, known as louping ill, but has also been isolated from cattle, horses and grouse [8]. Due to the economic losses it engenders, louping ill is an important veterinary problem in the UK, especially in Scotland [9]. Occasional human cases of LIV, diagnosed in farmers, butchers, abattoir and laboratory workers, appear to be related to occupational exposure rather than tick bites [10].

To this day, the capacity of a given tick species for viral acquisition and subsequent transmission, called vector competence, is not completely understood, in particular at the molecular level. Indeed, vector competence depends partly on cellular permissivity to viruses, which is largely driven by molecular biological processes. These are usually performed by physical assemblies of proteins (protein modules), whether in the form of stable protein complexes or functional modules, the latter including signal transduction pathways [11]. These two types of modules are connected by stable or intermittent protein–protein interactions (PPIs), respectively. The biological processes performed by these modules are vulnerable to viral hijacking, essentially manifested by the establishment of PPIs between viral proteins and critical components of the modules.

At present, relatively few complete PPI networks (interactomes) have been established for viruses, and focus has been on mosquito-borne flaviviruses [12]. To the best of our knowledge, no interactome has as yet been established for any tick-borne virus and its tick vector. Resolving such PPI networks and identifying molecular mechanisms that enable vector competence is essential to instructing risk assessment—through identification of competent vectors—and thereby improving preparedness [13, 14].

Flaviviruses are enveloped viruses possessing a positive-sense single-stranded RNA genome of 11 kb. The genome encodes a single polyprotein that is cleaved by host and viral proteases into three structural proteins—capsid protein (C), precursor membrane protein (pM) and envelope protein (E)—and seven non-structural proteins (NS1, NS2A, NS2B, NS3, NS4A, NS4B and NS5). The structural proteins are components of the viral particle, while non-structural proteins essentially take part in replication and evasion of the host immune response [15].

In this study, the PPI network established between TBEV/LIV and the tick *I. ricinus* was characterized for the first time. To this end, the entire set of TBEV and LIV proteins was screened against tick proteins encoded by a cDNA library generated from three *I. ricinus* embryo-derived cell lines using yeast two-hybrid methodology. For each virus, we identified 24 different interactions involving six viral proteins and 22 unique tick proteins, with all interactions being common to both viruses. According to our data, viral proteins target multiple protein modules implicated in critical biological pathways, such as those governing signal transduction, protein degradation and cytoskeletal function. Of note, the NS5 and pM viral proteins establish PPI with several tumor necrosis factor (TNF) receptor-associated factor (TRAF) proteins, which are essential adaptor proteins at the nexus of multiple signal transduction pathways. We presume that subversion of these processes by TBEV and LIV enhances viral survival in ticks and potentially viral transmission to mammalian hosts. Our results thus provide leads for the discovery of biological processes with a potential role in the vector competence of *I. ricinus* for TBEV and LIV.

## Methods

### Tick cDNA library

The tick cDNA library used in this study was derived from three *I. ricinus* embryonic cell lines, IRE11 [16], IRE/CTVM19 and IRE/CTVM20 [17]. The IRE11 cell line was established from eggs laid by a single female tick collected in Germany, while the IRE/CTVM19 and IRE/CTVM20 cell lines were established from a pool of eggs laid by four female ticks collected in the United Kingdom. Preparation of the *I. ricinus* cDNA library was subcontracted (Thermo Fisher Scientific). Briefly, mRNA of each tick cell line was isolated and reverse-transcribed by the subcontractor. An oligo(dT) primer was employed for reverse transcription, thus ensuring that the carboxyl termini of all tick proteins were intact.

### Viral production

The Hypr strain of TBEV (GenBank accession number U39292.1) was first isolated in 1953 from the blood of a 10-year-old child with TBE in the Brno region of the Czech Republic [18]. The LI3/1 strain of LIV (accession number KP144331.1), kindly provided by Nicholas Johnson (APHA, UK), was first isolated in 1962 from a sheep in Oban, Scotland [19]. Viral features have been summed up in Additional file 1. TBEV and LIV were amplified by five and three successive passages, respectively, on 80% confluent Vero cells (ATCC No. CCL-81) grown in Minimum Essential Medium (MEM; Thermo Fisher Scientific) supplemented with 2% fetal bovine serum (FBS) at a multiplicity of infection (MOI) of 0.001 Pfu/cell.

### Viral bait construction

TBEV and LIV viral RNAs were extracted using the QIAamp Viral RNA Mini Kit (QIAGEN, Hilden, Germany) according to the manufacturer’s protocol, and were reverse-transcribed using the Transcriptor High Fidelity cDNA Synthesis Kit (Roche, Basel, Switzerland). Briefly, viral RNAs were incubated with random hexamer primers (60 µM) for 10 min at 65 °C in a volume of 11.4 µL, and then chilled on ice. Transcriptase reaction buffer (4 µL), Protector RNase Inhibitor (20 U), dNTP (1 mM of each), DTT (5 mM) and Transcriptor High Fidelity Reverse Transcriptase (22 U) were added to the template-primer mix. Reverse transcription and inactivation of reverse transcriptase were achieved by incubation for 30 min at 55 °C and 5 min at 85 °C, respectively. Each viral bait was amplified by polymerase chain reaction (PCR) using Phusion High-Fidelity DNA Polymerase (Thermo Fisher Scientific) using the primers listed in Additional file 2. The primers contained a recombination sequence at the 5′ terminus, allowing insertion of each viral bait into a pDONR207 using a recombination cloning system (Gateway^®^ System; Invitrogen, Carlsbad, CA, USA). PCR was performed using 1 µL of Reverse Transcriptase (RT) product in high-fidelity buffer in a final volume of 50 µL, with 0.02 U/µL of Phusion Hot Start II DNA Polymerase, 200 µM of dNTPs and 0.3 µM of forward and reverse primers. Amplification was performed for 35 cycles as follows: 98 °C for 10 s, 56 °C for 15 s and 72 °C for 3 min.

### Cloning

Each amplified viral bait was cloned into pDONR207 (Additional file 3) using the Gateway^®^ cloning technology (Invitrogen). Briefly, 500 ng of pDONR207 vector was mixed with 500 ng of PCR product in the presence of 2 µL of BP Clonase™ in a final volume of 10 µL and incubated overnight at room temperature (RT). Similarly, viral ORFs cloned into pDONR207 were transferred into a Gateway^®^-compatible destination vector by an LR Clonase™ reaction. To this end, 500 ng of pDONR207 vector encoding each viral ORF insert and 500 ng of the destination vector were incubated overnight at RT with 2 µL of LR Clonase™ in a final volume of 10 µL. Each construction was confirmed by sequencing (Eurofins Genomics, Ebersberg, Germany).

### Bacterial transformation and plasmid DNA purification

All pDONR207 constructs were amplified after transformation of *E. coli* DH5α (NEB) (Additional file 1), following the manufacturer’s instructions. Plasmid DNA was extracted from bacteria using the NucleoSpin Plasmid kit (Macherey Nagel) according to the manufacturer’s recommendations.

### Yeast two-hybrid assay (Y2H)

**Fig. 1:**
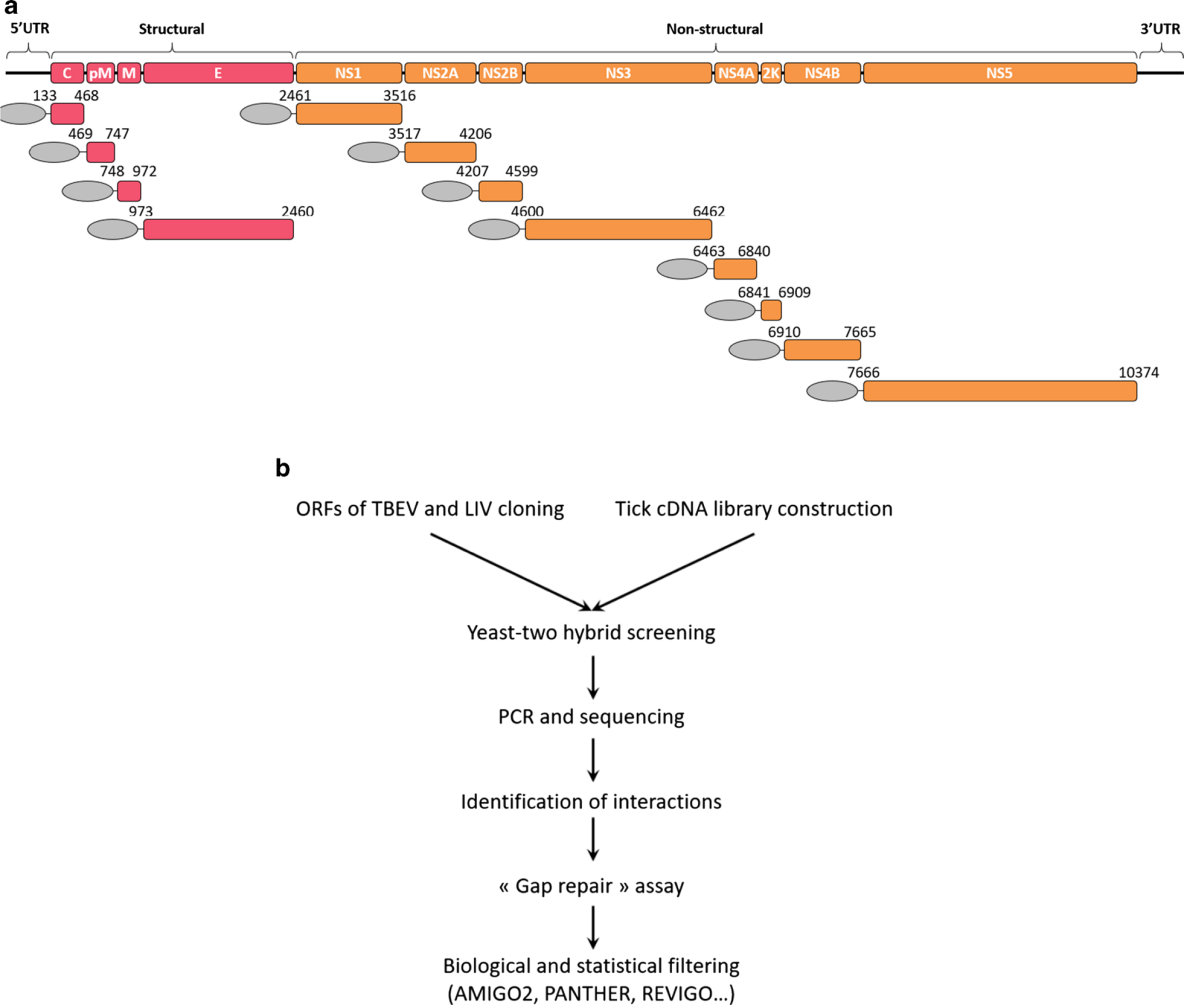
Strategy of TBEV and LIV protein screening. **a** Schematic representation of TBEV and LIV genome; pink and orange boxes represent structural and non-structural constructs, respectively, used for protein–protein interaction (PPI) screening. Grey circles represent the GAL4 DNA-binding domain used in the yeast two-hybrid assay. **b** Pipeline describing yeast two-hybrid screening and gap repair validation to define virus–tick PPIs

The coding sequences of viral baits were recombined into pPC97 (Additional file 3) to be expressed in frame downstream of the DNA-binding domain of Gal4 (Gal4-DB; Fig. 1a), while *I. ricinus* cDNA prey were recombined by a subcontractor into pDEST22 (Additional file 3) to be expressed in frame downstream of the activation domain of Gal4 (Gal4-AD). To perform Y2H experiments, Y2H Gold and Y187 yeast strains (Additional file 1) were transformed with vector pPC97 constructs encoding viral baits and the pDEST22 cDNA library encoding *I. ricinus* preys, respectively. Mating of the two strains was performed on selective medium lacking histidine, leucine and tryptophan and supplemented with 5 mM 3-amino-1,2,4-triazole (3-AT; Sigma-Aldrich, Saint-Louis, MO, USA). Since certain bait proteins may transactivate the *HIS3* reporter gene in Y2HGold yeast when expressed in frame with Gal4-DB [20], the transactivation level was measured for each viral bait and the concentration of 3-AT, which is a competitive inhibitor of the HIS3 enzyme, adjusted to optimize the stringency of the screen. After 6 days of incubation on selective medium, colonies were picked and purified on fresh selective medium over 3 weeks to maintain selection pressure and eliminate false-positive colonies [21]. Gal4-AD-cDNAs were amplified by PCR after zymolyase treatment using primers (Additional file 2) that hybridize within regions of pDEST22 flanking the cDNA inserts. PCR products were sequenced and cellular interactors were identified by automatic BLAST analysis.

### Gap repair (GR)

To identify new interactions and/or confirm interactions between viral baits and cellular prey, PCR products from the Y2H output were subjected to a GR procedure [22]. Briefly, yeast-carrying plasmids expressing DB-fused viral proteins were co-transformed with 10 ng of linearized pDEST22 empty vector and 3 µL of PCR product and plated on selective medium as described above. Homologous recombination between pDEST22 and the PCR product reconstituted AD-fused *I. ricinus* cDNA, and growth on medium lacking leucine, tryptophan and histidine was conditioned by physical interaction between viral and *I. ricinus* proteins. All AD-fused cDNAs encoding *I. ricinus* proteins were retested in yeast cells using this procedure. The strength of the interaction was also assessed by evaluating yeast growth in the presence of increasing 3-AT concentrations (5, 20, 35 and 50 mM). Last, to ensure a robust PPI network, only the PPIs supported by a GR confirmation were retained for further Gene Ontology (GO) analyses.

### In silico and Gene Ontology (GO) analyses

The Galaxy server was used to perform automatic BLASTX 2.2.31 analysis and identify orthologues of *I. ricinus* proteins from the *I. scapularis* genome [23]. The PPI network was generated with the open-source software Cytoscape v3.8.0 [24] and analyzed with NetworkAnalyzer [25]. The PANTHER (protein analysis through evolutionary relationships; http://pantherdb.org/http://pantherdb.org/) biological database was exploited using the AmiGO2 portal (http://amigo2.berkeleybop.org/amigo). The *I. scapularis* gene IDs were submitted to the GO term enrichment service using an *I. scapularis* organism filter in order to identify biological processes enriched in our dataset. The GO terms obtained ensued from the PANTHER overrepresentation test based on Fisher’s exact test and application of a false discovery rate (FDR) correction (Additional file 4). The REVIGO (reduce and visualize Gene Ontology; http://revigo.irb.hr/) web server was used to condense the list of GO terms and their associated *p*-values. The REVIGO algorithm was run with the default settings and generated a shorter GO term list without redundancy and with an associated frequency of the GO term in the UniProt database (Additional file 5). Functional descriptions for tick, mosquito, fly and human proteins were extracted from the UniProt (https://www.uniprot.org/), VectorBase (https://vectorbase.org/vectorbase/app) and FlyBase (https://flybase.org/) databases.

For comparison of bait sets, the fold enrichment score for each enriched GO term was partitioned among bait sets by multiplying the score by the fraction of proteins that belonged to each bait set within the set of proteins attributed to the GO term, to obtain an enrichment ratio. After replacing enrichment ratios assigned a null value by a value equivalent to half of the lowest non-null value in the complete bait set, all enrichment values were log2-transformed using the clusterMaker2 Cytoscape plugin. By means of the hierarchical cluster algorithm provided by this application, viral bait sets were grouped using the un-centered metric of similarity with no filtering and the pairwise average-linkage clustering method. Clusters were visualized using JTree TreeView.

## Results

### *Ixodes ricinus* cDNA library construction

Before initiating the Y2H screen, an *I. ricinus* cDNA library had to be constructed. To this end, tick cDNAs were cloned in frame downstream from the activation domain of Gal4. The cDNA was derived from three embryo-derived tick cell lines, that is, IRE11, IRE/CTVM19 and IRE/CTVM20. While IRE/CTVM19 and IRE/CTVM20 have been reported to be permissive to TBEV infection [26–28] and IRE/CTVM20 to LIV infection [27], the sensitivity of IRE11 to these viruses has not been reported.

### Generating a tick-borne flavivirus–tick PPI network

**Fig. 2:**
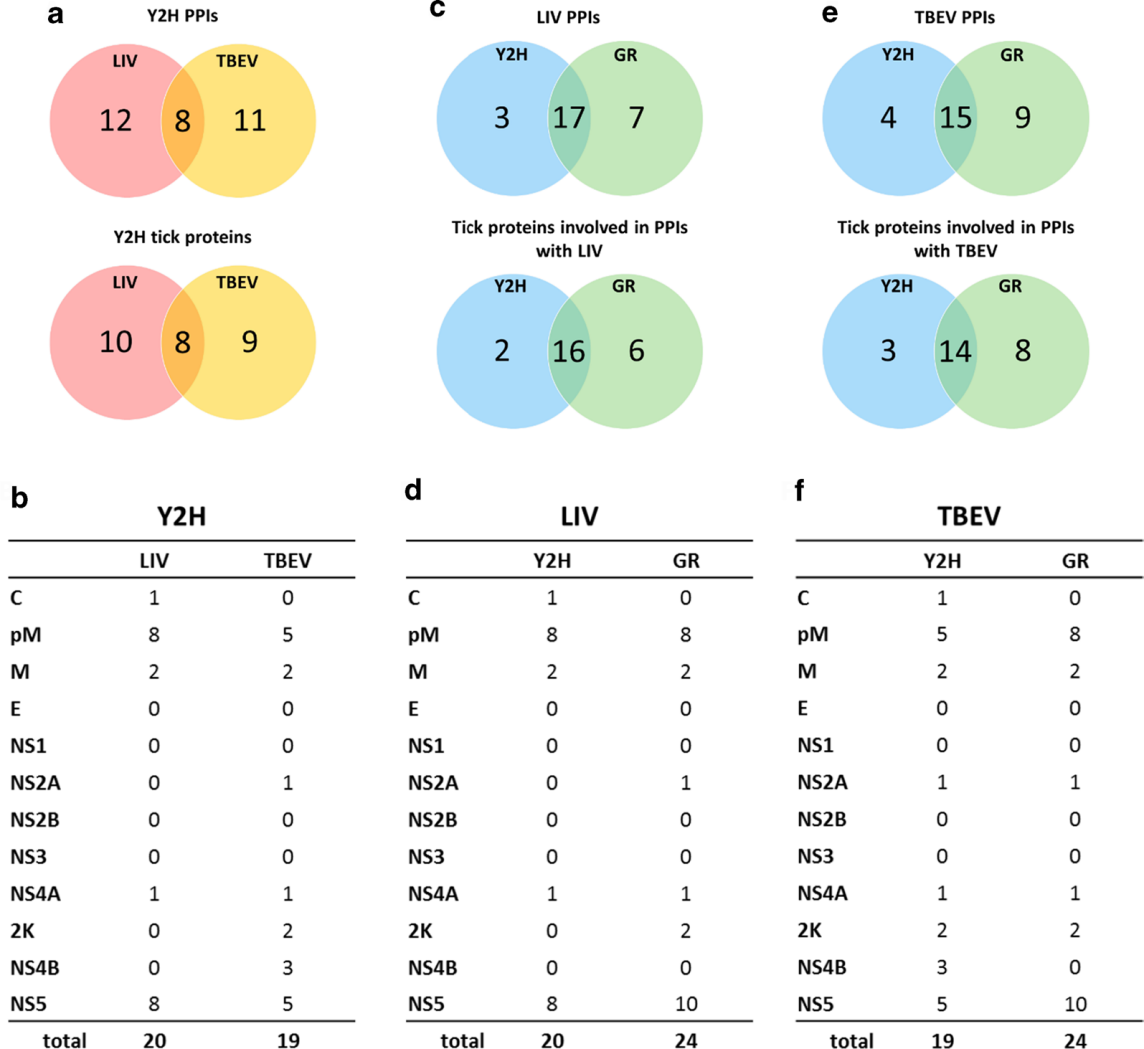
Summary of data from Y2H and GR screens. **a**, **c**, **e** Venn diagrams showing the number of protein–protein interactions (PPIs) and tick proteins obtained by Y2H screens for louping ill virus (LIV) and tick-borne encephalitis virus (TBEV); the number indicated in the intersection represents the PPIs or the tick proteins common to LIV and TBEV and identified using Y2H (**a**) and the PPIs or tick proteins confirmed by GR for LIV (**c**) and TBEV (**e**). **b**, **d**, **f** Tables indicating the PPIs identified for each viral protein identified in Y2H (**b**), for LIV (**d**) and TBEV (**f**). **g** Histogram indicating the number of PPIs identified for each viral bait for LIV and TBEV

In order to identify new PPIs between LIV, TBEV and *I. ricinus* proteins, we screened the *I. ricinus* cDNA library with the complete set of ORFs of TBEV and LIV (Fig. 1b). A single screen was performed against the tick cDNA library for each viral bait. Among 464 colonies picked for sequencing, comprising 131 and 333 for LIV and TBEV, respectively, 18 tick proteins interacting with LIV and 17 with TBEV were identified, of which eight were common to both viruses (Fig. 2a). These tick proteins are involved in 20 different PPIs with LIV and 19 with TBEV: of these PPIs, eight were common to both viruses at this step (Fig. 2a, b).

To verify the authenticity of the Y2H interactions and to determine whether tick preys found to interact with a single virus actually interacted with the cognate viral protein of the other virus, each of the tick preys was tested pairwise for its capacity to interact with the orthologous viral bait of both viruses. In particular, the reconstitution of a plasmid encoding a putative interactor by homologous recombination (GR) was used to verify pairwise PPI. That is, this strategy revealed whether tick prey proteins identified for a single virus were actually common to both. By this means, 17 of the 20 PPIs were confirmed for LIV (85%) and 15 of 19 (79%) for TBEV (Fig. 2c–f). These confirmed PPIs involved 22 distinct tick proteins, of which two, Ir8 and Ir12, interacted with two different viral proteins, that is, M and 2K for Ir8 and NS5 and pM for Ir12. Moreover, we were able to identify seven and nine virus–tick interactions for LIV and TBEV, respectively, that had not been detected in the initial Y2H screen. Conversely, seven PPIs implicating five tick proteins were not confirmed using this strategy and thus were not retained for further analysis. The majority (36/48) of the PPIs involved the pM (16) and the NS5 (20) viral proteins, with the remaining interactions concerning the M, NS2A, NS4A and 2K proteins (Fig. 2g). Of note, no interactions were evidenced for six viral proteins (C, E, NS1, NS2B, NS3 and NS4B) for either LIV or TBEV using the chosen approach. In sum, each tick prey identified was common to both viruses and interacted with the corresponding viral protein—or in two cases, viral proteins—of LIV and TBEV. In total, 48 PPIs were identified, 24 for each virus, and these involved 22 different tick proteins.

### Analysis of the tick-borne flavivirus—tick PPI network

**Fig. 3:**
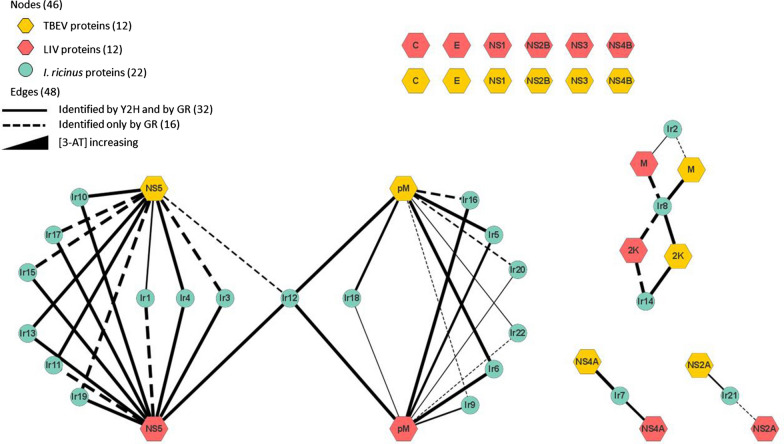
The TBEV/LIV-tick PPI network. Network of PPIs identified by yeast two-hybrid (Y2H) screens and gap repair (GR). LIV and TBEV proteins are represented in orange and red hexagons, respectively. Green nodes indicate tick (*Ixodes ricinus*) proteins identified in our screens. Edges represent PPIs. Solid lines denote interactions identified by both Y2H and GR. Dashed lines denote interactions only identified by GR. The line width represents the concentration of 3-amino-1,2,4-triazole used for the screen

Based on our high-throughput Y2H screen and GR confirmation, we used a network representation to visualize the LIV/TBEV-tick PPIs (Fig. 3). The network is thus composed of 46 nodes that are connected by 48 edges. Among the nodes, 24 and 22 represent viral or tick proteins, respectively. All of the latter interacted with the cognate protein of LIV and TBEV. Of the 48 edges, which symbolize the PPIs, 32 were identified with Y2H and GR, and 16 by GR. To address the strength of the PPIs, the GR screen was performed at a range of 3-AT concentrations (5, 20, 35 and 50 mM). Indeed, the higher the concentration of 3-AT that a PPI can withstand, the stronger the PPI is presumed to be. Thus, among the 48 identified PPIs, 31 could be evidenced with 50 mM of 3-AT, of which 16 and 15 were for LIV and TBEV, respectively. In most cases (13), the PPIs that withstood elevated stringency—suggesting strong physical interactions in the Y2H context—withstood for both viruses. In contrast, eight PPIs, five for LIV and three for TBEV, were only evidenced using 5 mM of 3-AT, suggesting a weaker interaction.

Gene assignment was performed for the 22 *I. ricinus* preys, arbitrarily designated Ir1 to Ir22, by BLASTX analysis against the *I. scapularis* genome. The *I. scapularis* orthologues are listed with their corresponding descriptions in Table 1. Nineteen *I. ricinus* proteins of the 22 identified by the Y2H screen displayed greater than 70% amino acid identity to *I. scapularis* proteins. In particular, the amino acid sequences of Ir5, Ir7, Ir12 and Ir17 were identical to those of ISCW003606, ISCW006829, ISCW012742 and ISCW021388, respectively, with a query cover ranging from 74 to 99% for Ir7, Ir12 and Ir17, though only 40% for Ir5. The remaining three proteins, Ir4, Ir19 and Ir21, were found to possess 22.6%, 37.2% and 55.4% amino acid sequence identity with ISCW002391, ISCW021630 and ISCW024393 proteins, respectively (Table 1; Additional file 6).

**Table 1:** *Ixodes scapularis* orthologues of *Ixodes ricinus* genes and encoded proteins identified by yeast two-hybrid screening and gap repair

*I. ricinus *ID	*I. scapularis* gene stable ID	*I. scapularis *protein name and/or description	% identity of *I. ricinus* protein to *I. scapularis* orthologue	% query cover	E-value
Ir1	ISCW000126	Conserved hypothetical protein	98.48	98	3.00E−40
Ir2	ISCW000339	Lamin Dm0-like	90.41	86	4.00E−177
Ir3	ISCW000545	Transcription factor jun-D	93.70	71	4.00E−122
Ir4	ISCW002391	TNF receptor-associated factor 4	22.58	71	5.00E−08
Ir5	ISCW003606	Dynein light chain 1	100.00	39	5.00E−57
Ir6	ISCW006409	Uncharacterized protein LOC8050906	79.76	73	3.00E−115
Ir7	ISCW006829	Ubiquilin-1	100.00	74	7.00E−68
Ir8	ISCW010489	Small glutamine-rich tetratricopeptide repeat-containing protein beta-like	99.15	77	5.00E−147
Ir9	ISCW011773	TNF receptor-associated factor 6-like	88.78	76	9.00E−134
Ir10	ISCW011803	Serine/threonine-protein kinase 26	97.75	71	9.00E−137
Ir11	ISCW011930	TNF receptor-associated factor 5-like	76.07	56	8.00E−85
Ir12	ISCW012742	Transcription initiation factor TFIID subunit 1	100.00	85	8.00E−180
Ir13	ISCW015959	RUN domain-containing protein 1	99.25	80	9.00E−117
Ir14	ISCW017452	Methionine-trna ligase	99.17	76	1.00E−143
Ir15	ISCW021017	Unconventional myosin-Va	100.00	78	3.00E−144
Ir16	ISCW021148	Adenosine kinase 2	97.45	65	2.00E−139
Ir17	ISCW021388	Serine/threonine-protein kinase 26	100.00	99	1.00E−69
Ir18	ISCW021545	Fibrillin-2	95.88	95	7.00E−159
Ir19	ISCW021630	TNF receptor-associated factor 4	36.39	87	1.00E−47
Ir20	ISCW023717	Protein SGT1 homolog	93.91	73	7.00E−135
Ir21	ISCW024393	E3 ubiquitin-protein ligase TRIM68-like	55.35	87	2.00E−65
Ir22	ISCW024608	Glutathione *S*-transferase	97.10	68	7.00E−95

Among the available annotated genomes, that of *I. scapularis* is the closest to *I. ricinus*. Nevertheless, the *I. scapularis* genome is still rather sparsely defined. In order to enrich annotation of the PPI network obtained by Y2H, we thus performed a BLASTX search in which the 22 *I. ricinus* sequences were used to query the genomes of three other organisms, namely, *Drosophila melanogaster*, *Aedes aegypti* and *Homo sapiens.* The orthologous proteins and their description are listed in Additional files 7, 8 and 9, respectively. No orthologues were identified for Ir4, Ir6, Ir19, Ir21 and Ir22 in *D. melanogaster*, for Ir6 and Ir21 in *Ae. aegypti*, or for Ir4 and Ir6 in *H. sapiens*.

### Tick cellular functions targeted by LIV and TBEV

**Fig. 4:**
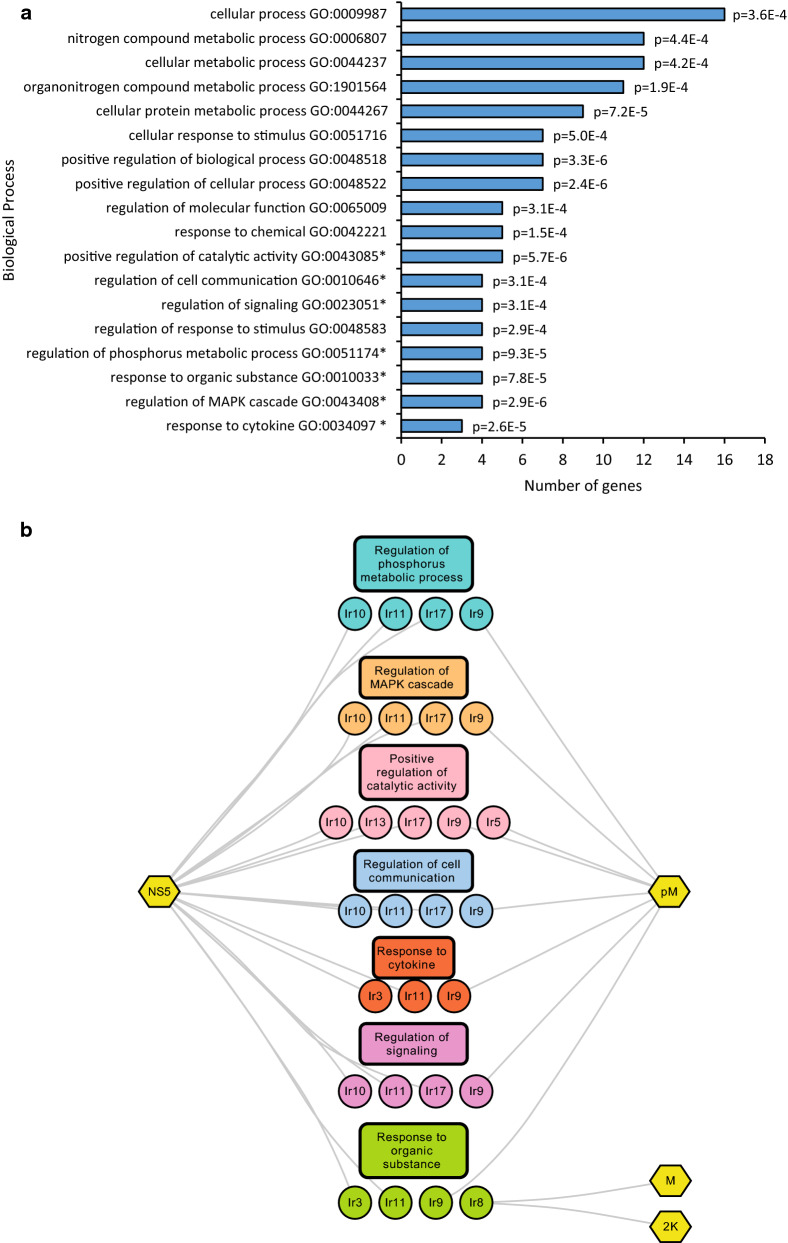
Functional enrichment analysis of tick cellular functions targeted by LIV and TBEV. **a** Histogram indicating statistical enrichment for specific biological processes (BP) determined by Gene Ontology analysis with PANTHER and REVIGO webserver. *Represents the 7 most specific biological processes according to the REVIGO (**b**) PPI network of *Ixodes ricinus* proteins identified by our screen and clustered into the 7 functional modules according to enriched GO terms of REVIGO. Yellow hexagons represent viral proteins, circle nodes represent *I. ricinus* proteins and rectangles indicate the enriched biological processes

To identify cellular processes that may be targeted by LIV and TBEV, we took advantage of the PANTHER classification system as accessed by the AMIGO2 portal. PANTHER is a large curated biological database of gene/protein families enabling functional characterization of genes and proteins based on their evolutionary relationships to those with known functions, by combining gene function, ontology, pathways and statistical analysis tools [29, 30]. We thus obtained 51 different enriched biological processes (BP) for our set of 22 *I. scapularis* prey (Additional file 4). To identify and visualize the most relevant BP, we used the REVIGO web server (Additional file 5). Briefly, this server summarizes long lists of GO terms by removing redundant terms using a simple clustering algorithm that relies on semantic similarity measures [31]. From the list of 51 GO terms and their associated *p*-values, 18 BP were retained (Fig. 4a). These GO terms are displayed with the filter of *I. ricinus* proteins to which they were attributed in Fig. 4b. Of note, seven GO terms possessed a frequency in the UniProt database lower than 1%, and corresponded to relatively specific terms within the hierarchical classification of GO terms, according to the REVIGO algorithm. These BP are “response to cytokine,” “regulation of MAPK cascade,” “regulation of phosphorus metabolic process,” “positive regulation of catalytic activity,” “response to organic substance,” “regulation of cell communication” and “regulation of signaling,” and are attributed to one or more of the eight *I. ricinus* proteins Ir3, Ir5, Ir8, Ir9, Ir10, Ir11, Ir13 and Ir17.

The TBEV/LIV and tick interactomes appeared to be significantly enriched in proteins (Ir3, Ir9, Ir11) with the attribute “response to cytokine.” All of these proteins possess a nucleic acid-binding domain, such as the bZIP domain for Ir3, a ring-type domain for Ir9 or a zinc-finger domain for Ir11, according to UniProt. Judging by the orthologues found in our reference genomes, Ir3 appears to be the transcription factor JunD, which belongs to the bZIP family and the Jun subfamily. It has been reported to play a role in dorsal closure, eggshell chorion assembly, epithelial morphogenesis and synaptic growth at neuromuscular junctions in *D. melanogaster* [32–34]. Ir9 was matched to a TRAF protein, TRAF6, in *I. scapularis*, a myosin-binding protein in *D. melanogaster*, an E3 ubiquitin-protein ligase NRDP1 in *Ae. aegypti* and a RING finger protein in *H. sapiens.* Query with Ir11 also returned a TRAF protein, or an E3 ubiquitin ligase in the case of *Ae. aegypti*. The TRAF proteins, of which most possess an E3 ubiquitin ligase activity, are signaling adaptor proteins that are involved in diverse biological processes, such as innate immunity and inflammation [35, 36].

The BP “regulation of MAPK cascade” was attributed to the Ir9, Ir10, Ir11 and Ir17 proteins. Ir10 and Ir17 possess a serine/threonine kinase (STK) domain, and the orthologue of *I. scapularis* Ir17 also comprises a SARAH (for Sav/Rassf/Hpo) domain. This last domain is a carboxy-terminal module of nearly 50 amino acids, which has been detected in three classes of tumor suppressors. In *D. melanogaster*, orthologues of Ir10 and Ir17 are depicted as germinal center kinases, which participate in a range of signaling pathways that regulate such cellular processes as apoptosis, cell proliferation, polarity and migration [37]. Regarding the human orthologues, STK25 is known as an oxidant stress-activated kinase, and may play a role in the response to environmental stress and regulation of diverse functions such as protein transport, cell adhesion and migration [38]. STK26, also called MST-4, has been described as a mediator of cell growth and reported to modulate apoptosis [39].

The Ir9, Ir10, Ir11 and Ir17 *I. ricinus* proteins are also related to the following three BP: “regulation of phosphorus metabolic process,” “regulation of signaling” and “regulation of cell communication.”

The BP “response to organic substance” is associated with Ir3, Ir8, Ir9 and Ir11. The Ir8 protein appears to be a cytosolic protein or associated to the membrane, according to UniProt. Moreover, Ir8 possesses three tetratricopeptide repeat (TPR) domains, a TPR domain being a degenerate sequence of ~ 34 amino acids, between amino acids 91 and 192. This motif enables proteins, such as Hsp70 and Hsp90, to act as scaffolds for the assembly of different multiprotein complexes [40]. Query of our reference genomes returned the orthologues Sgt, SUGT1 and SGTA for *D. melanogaster*, *Ae. aegypti* and *H. sapiens* genomes, respectively. Sgt, or small glutamine-rich tetratricopeptide protein, is predicted to have molecular adaptor activity, and has been implicated in positive regulation of chaperone-mediated protein folding [41, 42]. SUGT1 is thought to play a role in ubiquitination and thus in the proteasomal degradation of target proteins. SGTA is a co-chaperone protein reported to regulate the sorting of misfolded proteins to the proteasome, in collaboration with the BAG6 protein complex comprising BAG6, TRC35 and UBL4A proteins [43, 44].

Finally, the last highly specific BP “positive regulation of catalytic activity” involves the five *I. ricinus* proteins Ir5, Ir9, Ir10, Ir13 and Ir17. Query of the reference genomes with Ir5 returned dynein light chain 2 (DYNLL2), which is found in the microtubule motors dynein-1 and dynein-2 [45]. These motors carry out retrograde transport in the cytoplasm, with the latter being specialized in transport along motile and sensory cilia and flagella [46]. The *Drosophila* homolog of DYNLL2, Cut up, is thought to be implicated in dynein-mediated transport of neuronal proteasomes [47]. Ir13 appears to be a RUN domain-containing protein, judging by the orthologues recovered for *I. scapularis*, *Ae. aegypti* and *H. sapiens*. This protein has been implicated in the function of GTPases of the Rap and Rab families [48]. Furthermore, the RUN domain may play a role in the interaction of various proteins with cytoskeletal filaments [49] and be involved in intracellular protein transport.

**Fig. 5:**
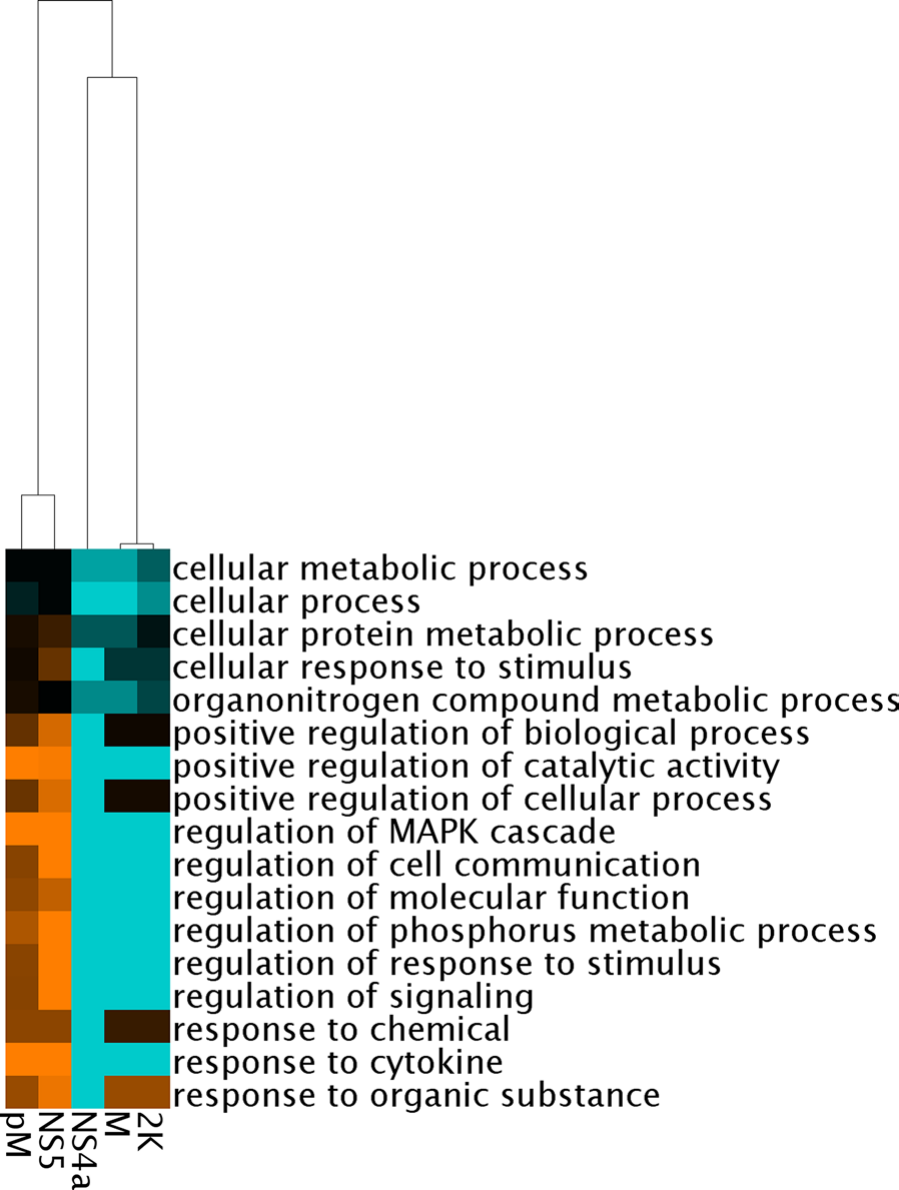
Clustering of viral bait according to GO enrichment ratio for biological processes. Clustering and visualization were performed using clusterMaker2 and JTree TreeView, respectively. 2K: 2K peptide, M: membrane protein, NS4a: Nonstructural protein 4a, NS5: Nonstructural protein 5, pM: pre-membrane protein

Finally, an unsupervised clustering of the viral bait set was applied according to the GO enrichment ratios for biological processes (Fig. 5). The viral protein bait set was clustered into two major groups (2K, M and NS4A versus pM and NS5), suggesting a possible community of biological functions targeted by the two groups of viral proteins.

## Discussion

In the arboviral life cycle, vector competence is defined as the intrinsic ability of an arthropod vector to acquire a virus during feeding on a viremic host and then to support sufficient viral replication for transmission to a new vertebrate host during a subsequent bloodmeal [50]. Viruses are obligate intracellular life forms, whose survival requires subversion of the host cell’s metabolic pathways and evasion of innate immunity. While for competent arthropod vectors, fitness seems little affected by arboviral infection, co-habitation implies extensive molecular crosstalk between virus and vector, of which binary PPIs are an essential element. In this study, we provide the first description of the PPI network connecting the tick-borne flaviviruses LIV and TBEV with their common vector, the *I. ricinus* tick. This network includes 48 PPIs, 24 for each virus, involving 22 distinct *I. ricinus* cellular proteins. The same set of PPIs was identified for both LIV and TBEV. Several protein–protein interactomes have already been published for flaviviruses with their mammalian hosts, especially humans. In this regard, PPI between tick-borne flaviviruses and human cells has been reported in a single publication [51]. Far fewer reports, however, have described PPI for flaviviruses with their arthropod vectors. The latter concern mosquito-borne flaviviruses and their mosquito vectors [52–54]. To the best of our knowledge, no PPI network has been previously published for a tick-borne flavivirus—or indeed for any tick-borne virus—and its tick vector.

### Choice of Y2H method

Ticks are the leading arthropod vectors responsible for the transmission of the broadest spectrum of pathogens to both humans and animals, and are second only to mosquitoes where transmission of human pathogens is concerned [55]. Despite their importance, relatively little attention has been paid to ticks compared with mosquitoes, and published data are thus comparatively limited. Indeed, the genome of the *I. scapularis* tick was published in 2016 but has still not been fully annotated [23]. More recently, Jia et al. [56] published the genomes of six tick species, including *Ixodes persulcatus*, and some efforts have been made to sequence and annotate the genome of *I. ricinus* [57, 58]. In this situation, the choice of strategies to establish a PPI network is limited. Methodologies such as affinity purification coupled to mass spectrometry (AP/MS), in which peptides derived from proteins of interest must be identified by referral to protein databases, are only effective when the sequence of the protein in question or its close homolog has been deposited in the database. For *I. ricinus* proteins, Y2H, in which the amino acid sequence of sizable portions of proteins may be recovered, is an appropriate choice.

### The cDNA library

To identify binary PPI between viral and tick proteins by the Y2H approach, viral “baits” were used to screen tick “preys” encoded by a cDNA library. The *I. ricinus* cDNA library was derived from three *I. ricinus* cell lines: IRE11, IRE/CTVM19 and IRE/CTVM20. By including multiple cell lines, we intended to enhance the diversity of the library. The library can be applied to the study of PPIs between *I. ricinus* and diverse tick-borne pathogens.

### Performance of Y2H

By Y2H and GR, our study revealed 24 PPIs for each virus. The fact that the same PPIs were recovered for the two viruses suggests that the interactions are likely to be authentic. Although the PPIs have not yet been validated by an unrelated approach, 80% of PPIs identified at least three times by Y2H can typically be confirmed in a second experimental system [59]. Nevertheless, while Y2H methodology provides high-quality binary PPI [60], individual screens are generally far from exhaustive. Indeed, a quarter of the PPI at best may be identified in a single Y2H screen [61]. In our study, *I. ricinus* interactors were not identified for C, E, NS1, NS2B, NS3 and NS4B proteins. Different explanations may be advanced for the absence of such interactors, and notably for the E protein whose principal role is to bind to the host receptor. First, cDNA for many interactors may be absent or poorly represented in prey cDNA libraries, which is why it is advisable to perform screens with multiple cDNA libraries to achieve a more complete view of the interactome [62, 63]. Second, certain viral proteins may be unable to gain entry or fold accurately in the yeast nucleus, where Y2H interactions occur, owing to physicochemical constraints, such as the presence of transmembrane helices, or to extended length [64]. This limitation could potentially be overcome by performing the screen with viral genes expressed as a set of domains, as demonstrated by Khadka et al. [65] for Dengue virus (DENV) genes. Indeed, this approach evidenced interactions that were missed when full-length genes were used, though using the Y2H methodology in both cases.

Moreover, certain other proteomic approaches, such as the abovementioned AP/MS, are capable of recovering interactors that associate with the proteins of interest in their natural cellular compartment. While AP/MS retrieves assemblies of interactors, of which only some interact directly with the protein of interest, it is useful for generation of “complex” interactome networks, complementary to the “binary” interactome established with the Y2H methodology. Thus, comparative study of flavivirus–host protein interaction networks between DENV and Zika virus (ZIKV) and human host and mosquito vectors revealed a role for NS5 in immune evasion through inhibition of ISG expression. The study also revealed several possible mechanisms for ZIKV microcephaly and neuropathogenesis implicating the NS4A protein [66]. The work of Hafirassou et al. [67], which focused on the role of DENV NS1 during viral replication, led to identification of a set of restriction and dependency factors that deeply affect DENV infection, and revealed cellular protein modules that are co-opted by the replication complex of DENV. The AP/MS methodology would thus be useful for investigating the entire interactomes of TBEV and LIV and *I. ricinus* and, more particularly, when the database of tick genes and proteins is more complete.

### Functional consequences

In order to be maintained and transmitted by the tick vector, tick-borne viruses must replicate within the arthropod vector, which can only be accomplished by co-opting the biological machinery of the host cell and evading its antiviral defenses, and notably by means of virus-cell PPIs. These PPI involved primarily NS5 and pM, but also NS4a, NS2a, 2k and M viral proteins. While interpretation of the full biological meaning of these PPI will require functional analyses, our Y2H screen revealed multiple cellular partners, and thus critical cellular functions of the tick that are presumably hijacked by TBEV and LIV, some of which we will highlight below.

#### Signal transduction/transcriptional response/protein degradation pathways

According to our Y2H screen, TBEV and LIV appear to target signal transduction pathways that control critical cellular processes. First, four tick proteins—Ir4, Ir9, Ir11 and Ir19—identified as interacting with the NS5 or pM viral proteins, belong to the family of TRAF proteins. TRAFs are a group of signaling adaptors that play diverse roles in inflammation and immunity [35]. In mammals they transduce signals delivered by diverse receptors, including those not only for TNF and related cytokines, but also for such pattern recognition receptors (PRR) as Toll-like receptors (TLR) and retinoic-inducible RIG-I-like receptors (RLR). Immediate downstream consequences of TRAF signaling include activation of the transcription factor NFκB and mitogen-activated protein kinases (MAPK). By analogy to cytosolic viral recognition in mammals, in which viral RNA is sensed by various RLR and leads to NF-κB activation, an antiviral response is triggered in insects upon recognition of cytosolic viral RNA by the RIG-I family member Dicer-2 [68, 69]. In *Culex* mosquitoes, flaviviral infection has been shown to activate the NF-κB ortholog Rel2 in a signaling cascade requiring TRAF [70]. In insects, activated Rel2 is translocated to the nucleus, where it induces expression of the interferon-like gene Vago [68–70]. Secreted Vago activates the Jak-STAT signaling pathway, and has been shown to induce an antiviral response to DENV [71] and ZIKV [72] in *Ae. aegypti* mosquitoes*.* We hypothesize that interactions between viral proteins (NS5 or pM) and tick TRAF-family members represent viral countermeasures to repress induction of the antiviral response. In particular, TRAF proteins Ir4, Ir9, Ir11 and Ir19 might possibly be sequestered by the interaction with viral proteins, thereby impeding restriction of viral replication.

The Ir3 protein was identified in our screen as an ortholog of JunD, which belongs to the Jun family of proteins whose members are components of activating protein-1 (AP-1) transcription factors. AP-1 designates a set of homo- or heterodimeric transcription factors composed of members of not only Jun, but also Fos, Maf and ATF protein families. AP-1 activity is regulated by MAPK signaling, and as such is influenced by a wide variety of extracellular stimuli, including viral infection and cytokines [73, 74]. AP-1 dimers recognize DNA motifs that are abundant in the genome, and thus influence the expression of a plethora of genes involved in diverse critical functions, including proliferation, differentiation, apoptosis and inflammation. While expression of AP-1 is regulated in a wide variety of viral infections, few binary interactions between viral proteins and AP-1 family members have been documented. Nevertheless, several proteins (bZIP, APH-2, -3, -4) expressed by the human T-cell leukemia virus (HTLV) have been shown to bind to various AP-1 components, including JunD, with variable impact on transcription depending on the factor and the HTLV subtype [75–77]. AP-1 dimers selectively recognize different DNA motifs, depending on the identity of the dimer, and interact cooperatively with other transcription factors. Thus, interaction between NS5 and JunD, which is expected to affect only JunD-containing AP-1 dimers, is likely to affect transcription of a subset of AP-1-regulated genes. Whether such interaction serves to increase an advantageous transcriptional response, or repress an unfavorable one such as an antiviral response triggered by DICER-2 sensing of viral RNA, remains to be determined.

The Ir7 protein, which interacts with NS4A, is an ortholog of ubiquilin-1. Ubiquilins play important roles in both cytosolic and transmembrane protein degradation pathways, not only as adaptor molecules that shuttle polyubiquitinated proteins to the proteosome, but also as regulators of autophagy and endoplasmic reticulum-associated protein degradation (for a review see [78]). Proteins of several viruses have been found to interact with ubiquilin family members, as is notably the case for the NS5B protein of hepatitis C virus, whose interaction with human ubiquilin-1 diminishes the half-life of NS5B and which may regulate viral replication [79], and for the NS4A protein of West Nile virus [80]. Intriguingly, the TLR3 antiviral pathway may be subject to inhibition by ubiquilin-1 through its interaction with the TLR domain, according to a Y2H-based study [81]. It is thus likely that physical interaction between tick ubiquilin (Ir7) and NS4A of TBEV and LIV affects viral interplay with protein degradation pathways, but whether to the viruses’ advantage or disadvantage remains to be elucidated.

#### Cytoskeleton

Two cellular proteins, Ir5 and Ir15, retrieved as interacting with the pM and NS5 viral proteins, respectively, correspond to known components of cytoskeletal motor complexes. Ir5 was identified as a member of the family of dynein light chains, which are highly conserved proteins originally identified as being components of both the dynein microtubular and myosin Va motor complexes [82]. Various proteins of diverse viruses have been shown to bind to dynein light chains, but whether such interactions permit coupling of viruses to molecular motors and hence use of microtubules or F-actin tracks for intracellular viral transport is still open to debate (for review see [83]). Dynein light chains have since been shown to bind to a plethora of cellular proteins and have been linked to a large number of cellular functions. Of note, the *Drosophila* homolog, Cut up, has been proposed to be implicated in dynein-mediated transport of neuronal proteasomes [47], and human DYNLL2—in conjunction with myosin Va—in sequestration of pro-apoptotic factors, suggesting a role for these last two proteins as pro-survival molecules [84].

Orthologs of Ir15, on the other hand, were identified as belonging to the myosin V family of unconventional myosin motor proteins, namely myosin 5a in *H. sapiens* or Didum in *D. melanogaster*. Myosin V is involved in short-range transport of diverse intracellular cargo—such as organelles, vesicles, protein complexes and mRNA—along actin filaments [85]. To the best of our knowledge, no published report of a binary interaction between a viral protein and myosin V exists in the literature, although human myosin 5a has been shown to be important for early events during infection by rhinovirus B14 [86], for transport of herpes simplex virus 1 from the trans-Golgi network toward the plasma membrane [87] and for nuclear egress of human cytomegalovirus [88]. Moreover, the myosin V paralog 5c has been implicated in the release of DENV [89]. NS5 being a nonstructural protein, it would be unlikely to promote viral egress by direct coupling of viral particles to myosin V. Rather, the interaction might promote intracellular mobility of the NS5 protein itself or the proteins and/or cargo with which NS5 interacts, or might disrupt coupling of myosin V to its conventional cargo, as remains to be elucidated.

#### Other processes

Our PPI network revealed diverse processes that appear to be targeted by TBEV and LIV, such as cell proliferation and cell cycle regulation. The Nop seven-associated 2 (NSA2) protein—an orthologue of Ir1 in our screen and also known as TINP1 (TGF-β-inducible nuclear protein 1)—is a nucleolar protein involved in cell proliferation and cell cycle regulation [90]. NSA2 is evolutionarily conserved across different species, and required for ribosome biogenesis and synthesis [91]. Flaviviruses have been reported to disrupt ribosome biogenesis [92], in particular for Japanese encephalitis virus (JEV) and DENV via the interaction of capsid with ribosome biogenesis factors [93, 94]. Our screen suggests that the NS5 of TBEV and LIV could also disrupt ribosome biogenesis, presumably to decrease the translation of host proteins and to promote the translation of the viral polyprotein instead. By interfering with cell proliferation, NS5 could increase the number of cells that potentially could be infected by the virus, and so raise the infectious viral particle production. In a recent study, cell proliferation rate was shown to influence mosquito infection and vector competence for DENV [95].

#### Significance of PPI for ecology of tick-borne viruses

Despite high genetic proximity, TBEV and LIV are ecologically distinct, possibly in relation to distinct PPI maintained with their arthropod vectors, mammalian reservoir or accidental hosts. In our screen, identical PPI with the *I. ricinus* tick cells were recovered for both viruses. As mentioned above, Y2H screens are never exhaustive, and in all likelihood only a small fraction of existing PPI was recovered in this study. We therefore cannot exclude that virus-specific PPIs with *I. ricinus* exist. Nevertheless, our results do not provide evidence that TBEV and LIV interact differently with *I. ricinus*, and thus encourage investigation of PPI between these viruses and alternative tick vectors [96, 97] and reservoir species, so as to provide molecular understanding of the distinctive ecology of these viruses.

## Conclusion

The present study, providing the PPI network established between two tick-borne flaviviruses (TBEV and LIV) and their arthropod vector *I. ricinus*, represents the first published protein–protein interaction for a tick-borne virus with its arthropod host. Our study reveals that viral proteins of TBEV and LIV interact with multiple protein modules of host cells, including critical pathways governing signal transduction, transcription, protein degradation and cytoskeletal function. Viral intrusion into these pathways provides multiple avenues for discovery of the molecular determinants of vector competence of *I. ricinus* for these viruses, which may be of general importance for other tick-borne viruses. Given the relatively modest number of interactions recovered in the present screen, and the scarcity of interactors that overlap with those identified in other flavivirus-arthropod studies, it is likely that identification of such PPIs is far from reaching the saturation point. Identification of PPIs for tick-borne flaviviruses should thus be pursued, but could also be combined with gene perturbation approaches (RNAi, CRISPR/Cas9), to gain further insight into the function of tick proteins during replication of TBEV, LIV and other tick-borne flaviviruses.

## Supplementary Information

**Additional file 1.** List of strains and cell lines used in this study.

**Additional file 2.** List of primers used for Gateway^®^ cloning in the present study.

**Additional file 3.** List of vectors used for the clonings in this study.

**Additional file 4.** List of GO terms obtained with PANTHER.

**Additional file 5.** List of GO terms summarized with REVIGO.

**Additional file 6.** Protein alignment of Ir1.

**Additional file 7.** *Drosophila melanogaster* orthologues of *Ixodes ricinus* genes and encoded proteins identified by yeast two-hybrid screening and gap repair.

**Additional file 8.** *Aedes aegypti* orthologues of *Ixodes ricinus* genes and encoded proteins identified by yeast two-hybrid screening and gap repair.

**Additional file 9.** *Homo sapiens* orthologues of *Ixodes ricinus* genes and encoded proteins identified by yeast two-hybrid screening and gap repair.

## Data Availability

All the data are included within the article and its additional files. The tick cDNA library will be made available upon request.

## Abbreviations

TBEV: Tick-borne encephalitis virus
LIV: Louping ill virus
PPI: Protein–protein interaction
Y2H: Yeast two-hybrid
GR: Gap repair
C: Capsid protein
pM: Precursor membrane protein
M: Membrane protein
E: Envelope protein
NS1: Non-structural protein 1
NS2A: Non-structural protein 2A
NS2B: Non-structural protein 2B
NS3: Non-structural protein 3
NS4A: Non-structural protein 4A
2K: 2K peptide
NS4B: Non-structural protein 4B
NS5: Non-structural protein 5
MAPK: Mitogen-activated protein kinase
*I. ricinus*: *Ixodes ricinus*
*I. scapularis*: *Ixodes scapularis*
*D. melanogaster*: *Drosophila melanogaster*
*Ae. Aegypti*: *Aedes aegypti*
*H. sapiens*: *Homo sapiens*
*E. coli*: *Escherichia coli*
TNF: Tumor necrosis factor
TRAF: TNF receptor-associated factor
FBS: Fetal bovine serum
MOI: Multiplicity of infection
3-AT: 3-Amino-1,2,4-triazole
PRR: Pattern recognition receptor
TLR: Toll-like receptor
RLR: RIG-I-like receptor
GO: Gene Ontology
STK: Serine/threonine kinase
DYNLL2: Dynein light chain 2
AP-1: Activating protein-1
AP/MS: Affinity purification coupled to mass spectrometry
DENV: Dengue virus
ZIKV: Zika virus
NK-κB: Nuclear factor-kappa B
HTLV: Human T-cell leukemia virus
JEV: Japanese encephalitis virus
TINP1: TGF-β-inducible nuclear protein 1
RNAi: RNA interference

## References

[1] Labuda M, Nuttall PA (2004). Tick-borne viruses. Parasitology.

[2] Calisher CH (1988). Antigenic classification and taxonomy of flaviviruses (family Flaviviridae) emphasizing a universal system for the taxonomy of viruses causing tick-borne encephalitis. Acta Virol.

[3] Gritsun TS, Nuttall PA, Gould EA (2003). Tick-borne flaviviruses. Adv Virus Res.

[4] Süss J (2011). Tick-borne encephalitis 2010: epidemiology, risk areas, and virus strains in Europe and Asia—an overview. Ticks Tick Borne Dis.

[5] Gritsun TS, Lashkevich VA, Gould EA (2003). Tick-borne encephalitis. Antiviral Res.

[6] Lindquist L, Vapalahti O (2008). Tick-borne encephalitis. Lancet.

[7] Pool WA, Brownlee A, Wilson DR (1930). The etiology of “louping-ill”. J Comp Pathol Ther.

[8] Jeffries CL, Mansfield KL, Phipps LP, Wakeley PR, Mearns R, Schock A (2014). Louping ill virus: an endemic tick-borne disease of Great Britain. J Gen Virol.

[9] Gilbert L (2016). Louping ill virus in the UK: a review of the hosts, transmission and ecological consequences of control. Exp Appl Acarol.

[10] Davidson MM, Williams H, Macleod JA (1991). Louping ill in man: a forgotten disease. J Infect.

[11] Spirin V, Mirny LA (2003). Protein complexes and functional modules in molecular networks. Proc Natl Acad Sci USA.

[12] Kovanich D, Saisawang C, Sittipaisankul P, Ramphan S, Kalpongnukul N, Somparn P (2019). Analysis of the Zika and Japanese Encephalitis virus NS5 interactomes. J Proteome Res.

[13] de la Fuente J, Antunes S, Bonnet S, Cabezas-Cruz A, Domingos AG, Estrada-Peña A (2017). Tick–pathogen interactions and vector competence: identification of molecular drivers for tick-borne diseases. Front Cell Infect Microbiol.

[14] Grabowski JM, Hill CA (2017). A roadmap for tick-borne flavivirus research in the “Omics” era. Front Cell Infect Microbiol.

[15] Lindenbach BD, Thiel H-J, Rice CM, Knipe DM, Howley PM (2007). *Flaviviridae*: the viruses and their replication. Fields virology.

[16] Simser JA, Palmer AT, Fingerle V, Wilske B, Kurtti TJ, Munderloh UG (2002). *Rickettsia monacensis* sp. nov., a spotted fever group rickettsia, from ticks (*Ixodes ricinus*) collected in a European city park. Appl Environ Microbiol.

[17] Bell-Sakyi L, Zweygarth E, Blouin EF, Gould EA, Jongejan F (2007). Tick cell lines: tools for tick and tick-borne disease research. Trends Parasitol.

[18] Pospíšil L, Jandásek L, Pešek J (1954). Isolation of new strains of meningoencephalitis virus in the Brno region during the summer of 1953. Lek List.

[19] Mansfield KL, Morales AB, Johnson N, Ayllón N, Höfle U, Alberdi P (2015). Identification and characterization of a novel tick-borne flavivirus subtype in goats (*Capra hircus*) in Spain. J Gen Virol.

[20] Titz B, Thomas S, Rajagopala SV, Chiba T, Ito T, Uetz P (2006). Transcriptional activators in yeast. Nucleic Acids Res.

[21] Vidalain PO, Boxem M, Ge H, Li S, Vidal M (2004). Increasing specificity in high-throughput yeast two-hybrid experiments. Methods.

[22] Walhout AJM, Vidal M (2001). High-throughput yeast two-hybrid assays for large-scale protein interaction mapping. Methods.

[23] Gulia-Nuss M, Nuss AB, Meyer JM, Sonenshine DE, Roe RM, Waterhouse RM (2016). Genomic insights into the *Ixodes scapularis* tick vector of Lyme disease. Nat Commun.

[24] Shannon P, Markiel A, Ozier O, Baliga NS, Wang JT, Ramage D (2003). Cytoscape: a software environment for integrated models of biomolecular interaction networks. Genome Res.

[25] Assenov Y, Ramírez F, Schelhorn SESE, Lengauer T, Albrecht M (2008). Computing topological parameters of biological networks. Bioinformatics.

[26] Weisheit S, Villar M, Tykalová H, Popara M, Loecherbach J, Watson M (2015). *Ixodes scapularis* and *Ixodes ricinus* tick cell lines respond to infection with tick-borne encephalitis virus: transcriptomic and proteomic analysis. Parasites Vectors.

[27] Mansfield KL, Cook C, Ellis RJ, Bell-Sakyi L, Johnson N, Alberdi P (2017). Tick-borne pathogens induce differential expression of genes promoting cell survival and host resistance in *Ixodes ricinus* cells. Parasites Vectors.

[28] Růžek D, Bell-Sakyi L, Kopecký J, Grubhoffer L (2008). Growth of tick-borne encephalitis virus (European subtype) in cell lines from vector and non-vector ticks. Virus Res.

[29] Mi H, Muruganujan A, Thomas PD (2013). PANTHER in 2013: modeling the evolution of gene function, and other gene attributes, in the context of phylogenetic trees. Nucleic Acids Res.

[30] Thomas PD, Kejariwal A, Campbell MJ, Mi H, Diemer K, Guo N (2003). PANTHER: a browsable database of gene products organized by biological function, using curated protein family and subfamily classification | Nucleic Acids Research | Oxford Academic. Nucleic Acids Res.

[31] Supek F, Bošnjak M, Škunca N, Šmuc T (2011). Revigo summarizes and visualizes long lists of gene ontology terms. PLoS ONE.

[32] Riesgo-Escovar JR, Hafen E (1997). *Drosophila* Jun kinase regulates expression of *decapentaplegic* via the ETS-domain protein Aop and the AP-1 transcription factor DJun during dorsal closure. Genes Dev.

[33] Kockel L, Zeitlinger J, Staszewski LM, Mlodzik M, Bohmann D (1997). Jun in *Drosophila* development: redundant and nonredundant functions and regulation by two MAPK signal transduction pathways. Genes Dev.

[34] Franciscovich AL, Vrailas Mortimer AD, Freeman AA, Gu J, Sanyal S (2008). Overexpression screen in drosophila identifies neuronal roles of GSK-3β/*shaggy* as a regulator of AP-1-dependent developmental plasticity. Genetics.

[35] Bishop GA, Abdul-Sater AA, Watts TH (2019). Editorial: TRAF proteins in health and disease. Front Immunol.

[36] Xie P (2013). TRAF molecules in cell signaling and in human diseases. J Mol Signal.

[37] Yin H, Shi Z, Jiao S, Chen C, Wang W, Greene MI (2012). Germinal center kinases in immune regulation. Cell Mol Immunol.

[38] Preisinger C, Short B, De Corte V, Bruyneel E, Haas A, Kopajtich R (2004). YSK1 is activated by the Golgi matrix protein GM130 and plays a role in cell migration through its substrate 14–3-3ζ. J Cell Biol.

[39] Lin JL, Chen HC, Fang HI, Robinson D, Kung HJ, Shih HM (2001). MST4, a new Ste20-related kinase that mediates cell growth and transformation via modulating ERK pathway. Oncogene.

[40] Scheufler C, Brinker A, Bourenkov G, Pegoraro S, Moroder L, Bartunik H (2000). Structure of TPR domain-peptide complexes: critical elements in the assembly of the Hsp70-Hsp90 multichaperone machine. Cell.

[41] Uytterhoeven V, Lauwers E, Maes I, Miskiewicz K, Melo MN, Swerts J (2015). Hsc70-4 deforms membranes to promote synaptic protein turnover by endosomal microautophagy. Neuron.

[42] Tobaben S, Thakur P, Fernández-Chacón R, Südhof TC, Rettig J, Stahl B (2001). A trimeric protein complex functions as a synaptic chaperone machine. Neuron.

[43] Leznicki P, High S (2012). SGTA antagonizes BAG6-mediated protein triage. Proc Natl Acad Sci USA.

[44] Krysztofinska EM, Martínez-Lumbreras S, Thapaliya A, Evans NJ, High S, Isaacson RL (2016). Structural and functional insights into the E3 ligase, RNF126. Sci Rep.

[45] Asante D, Stevenson NL, Stephens DJ (2014). Subunit composition of the human cytoplasmic dynein-2 complex. J Cell Sci.

[46] Roberts AJ, Kon T, Knight PJ, Sutoh K, Burgess SA (2013). Functions and mechanics of dynein motor proteins. Nat Rev Mol Cell Biol.

[47] Kreko-Pierce T, Eaton BA (2017). The *Drosophila* LC8 homolog cut up specifies the axonal transport of proteasomes. J Cell Sci.

[48] Callebaut I, De Gunzburg J, Goud B, Mornon JP (2001). RUN domains: a new family of domains involved in Ras-like GTPase signaling. Trends Biochem Sci.

[49] Mari M, Macia E, Le Marchand-Brustel Y, Cormont M (2001). Role of the FYVE finger and the RUN domain for the subcellular localization of Rabip4. J Biol Chem.

[50] Jongejan F, Uilenberg G (2004). The global importance of ticks. Parasitology.

[51] Le Breton M, Meyniel-Schicklin L, Deloire A, Coutard B, Canard B, de Lamballerie X (2011). Flavivirus NS3 and NS5 proteins interaction network: a high-throughput yeast two-hybrid screen. BMC Microbiol.

[52] Tham HW, Balasubramaniam VRMT, Chew MF, Ahmad H, Hassan SS (2015). Protein–protein interactions between *A. aegypti* midgut and dengue virus 2: two-hybrid screens using the midgut cDNA library. J Infect Dev Ctries.

[53] Guo X, Xu Y, Bian G, Pike AD, Xie Y, Xi Z (2010). Response of the mosquito protein interaction network to dengue infection. BMC Genom.

[54] Mairiang D, Zhang H, Sodja A, Murali T, Suriyaphol P, Malasit P (2013). Identification of new protein interactions between dengue fever virus and its hosts, human and mosquito. PLoS ONE.

[55] Mansfield KL, Jizhou L, Phipps LP, Johnson N (2017). Emerging tick-borne viruses in the twenty-first century. Front Cell Infect Microbiol.

[56] Jia N, Wang J, Shi W, Du L, Sun Y, Zhan W (2020). Large-scale comparative analyses of tick genomes elucidate their genetic diversity and vector capacities. Cell.

[57] Cramaro WJ, Revets D, Hunewald OE, Sinner R, Reye AL, Muller CP (2015). Integration of *Ixodes ricinus* genome sequencing with transcriptome and proteome annotation of the naïve midgut. BMC Genom.

[58] Cramaro WJ, Hunewald OE, Bell-Sakyi L, Muller CP (2017). Genome scaffolding and annotation for the pathogen vector *Ixodes ricinus* by ultra-long single molecule sequencing. Parasites Vectors.

[59] Li S, Armstrong CM, Bertin N, Ge H, Milstein S, Boxem M (2004). A map of the interactome network of the metazoan *C. elegans*. Science.

[60] Chen WY, Ho KC, Leu JH, Liu KF, Wang HC, Kou GH (2008). WSSV infection activates STAT in shrimp. Dev Comp Immunol.

[61] Braun P, Tasan M, Dreze M, Barrios-Rodiles M, Lemmens I, Yu H (2009). An experimentally derived confidence score for binary protein–protein interactions. Nat Methods.

[62] Kim MS, Pinto SM, Getnet D, Nirujogi RS, Manda SS, Chaerkady R (2014). A draft map of the human proteome. Nature.

[63] Wilhelm M, Schlegl J, Hahne H, Gholami AM, Lieberenz M, Savitski MM (2014). Mass-spectrometry-based draft of the human proteome. Nature.

[64] Jensen LJ, Bork P (2008). Biochemistry: not comparable, but complementary. Science.

[65] Khadka S, Vangeloff AD, Zhang C, Siddavatam P, Heaton NS, Wang L (2011). A physical interaction network of dengue virus and human proteins. Mol Cell Proteom.

[66] Shah PS, Link N, Jang GM, Sharp PP, Zhu T, Swaney DL (2018). Comparative flavivirus-host protein interaction mapping reveals mechanisms of dengue and Zika virus pathogenesis. Cell.

[67] Hafirassou ML, Meertens L, Umaña-Diaz C, Labeau A, Dejarnac O, Bonnet-Madin L (2018). A global interactome map of the dengue virus NS1 identifies virus restriction and dependency host factors. Cell Rep.

[68] Deddouche S, Matt N, Budd A, Mueller S, Kemp C, Galiana-Arnoux D (2008). The DExD/H-box helicase Dicer-2 mediates the induction of antiviral activity in drosophila. Nat Immunol.

[69] Paradkar PN, Trinidad L, Voysey R, Duchemin JB, Walker PJ (2012). Secreted Vago restricts West Nile virus infection in *Culex* mosquito cells by activating the Jak-STAT pathway. Proc Natl Acad Sci USA.

[70] Paradkar PN, Duchemin J-B, Voysey R, Walker PJ (2014). Dicer-2-dependent activation of *Culex* Vago occurs via the TRAF-Rel2 signaling pathway. PLoS Negl Trop Dis.

[71] Souza-Neto JA, Sim S, Dimopoulos G (2009). An evolutionary conserved function of the JAK-STAT pathway in anti-dengue defense. Proc Natl Acad Sci USA.

[72] Angleró-Rodríguez YI, MacLeod HJ, Kang S, Carlson JS, Jupatanakul N, Dimopoulos G (2017). *Aedes aegypti* molecular responses to Zika Virus: modulation of infection by the toll and Jak/Stat immune pathways and virus host factors. Front Microbiol.

[73] Eferl R, Wagner EF (2003). AP-1: a double-edged sword in tumorigenesis. Nat Rev Cancer.

[74] Hernandez JM, Floyd DH, Weilbaecher KN, Green PL, Boris-Lawrie K (2008). Multiple facets of junD gene expression are atypical among AP-1 family members. Oncogene.

[75] Thébault S, Basbous J, Hivin P, Devaux C, Mesnard JM (2004). HBZ interacts with JunD and stimulates its transcriptional activity. FEBS Lett.

[76] Larocque E, Andre-Arpin C, Borowiak M, Lemay G, Switzer WM, Duc Dodon M (2014). Human T-cell leukemia virus type 3 (HTLV-3) and HTLV-4 antisense-transcript-encoded proteins interact and transactivate Jun family-dependent transcription via their atypical bZIP motif. J Virol.

[77] Kuhlmann AS, Villaudy J, Gazzolo L, Castellazzi M, Mesnard JM, Dodon MD (2007). HTLV-1 HBZ cooperates with JunD to enhance transcription of the human telomerase reverse transcriptase gene (hTERT). Retrovirology.

[78] Jantrapirom S, Piccolo LL, Pruksakorn D, Potikanond S, Nimlamool W (2020). Ubiquilin networking in cancers. Cancers.

[79] Gao L, Tu H, Shi ST, Lee K-J, Asanaka M, Hwang SB (2003). Interaction with a ubiquitin-like protein enhances the ubiquitination and degradation of hepatitis C virus RNA-dependent RNA polymerase. J Virol.

[80] Li M, Johnson JR, Truong B, Kim G, Weinbren N, Dittmar M (2019). Identification of antiviral roles for the exon–junction complex and nonsense-mediated decay in flaviviral infection. Nat Microbiol.

[81] Biswas N, Liu S, Ronni T, Aussenberg SE, Liu W, Fujita T (2011). The ubiquitin-like protein PLIC-1 or ubiquilin 1 inhibits TLR3-Trif signaling. PLoS ONE.

[82] Rapali P, Szenes Á, Radnai L, Bakos A, Pál G, Nyitray L (2011). DYNLL/LC8: a light chain subunit of the dynein motor complex and beyond. FEBS J.

[83] Dodding MP, Way M (2011). Coupling viruses to dynein and kinesin-1. EMBO J.

[84] Izidoro-Toledo TC, Borges AC, Araujo DD, Leitão Mazzi DPS, Junior FON, Sousa JF (2013). A myosin-Va tail fragment sequesters dynein light chains leading to apoptosis in melanoma cells. Cell Death Dis.

[85] Fili N, Toseland CP (2020). Unconventional myosins: how regulation meets function. Int J Mol Sci.

[86] Real-Hohn A, Provance DW, Gonçalves RB, Denani CB, de Oliveira AC, Salerno VP (2017). Impairing the function of MLCK, myosin Va or myosin Vb disrupts Rhinovirus B14 replication. Sci Rep.

[87] Roberts KL, Baines JD (2010). Myosin Va enhances secretion of herpes simplex virus 1 virions and cell surface expression of viral glycoproteins. J Virol.

[88] Wilkie AR, Sharma M, Pesola JM, Ericsson M, Fernandez R, Coen DM (2018). A role for myosin Va in human cytomegalovirus nuclear egress. J Virol.

[89] Xu XF, Chen ZT, Gao N, Zhang JL, An J (2009). Myosin Vc, a member of the actin motor family associated with Rab8, is involved in the release of DV2 from HepG2 cells. Intervirology.

[90] Zhang H, Ma X, Shi T, Song Q, Zhao H, Ma D (2010). NSA2, a novel nucleolus protein regulates cell proliferation and cell cycle. Biochem Biophys Res Commun.

[91] Xing J, Nan X, Cui Q, Ma W, Zhao H (2018). Nop-7-associated 2 (NSA2) is required for ribosome biogenesis and protein synthesis. Biochem Biophys Res Commun.

[92] Sotcheff S, Routh A (2020). Understanding flavivirus capsid protein functions: the tip of the iceberg. Pathogens.

[93] Tsuda Y, Mori Y, Abe T, Yamashita T, Okamoto T, Ichimura T (2006). Nucleolar protein B23 interacts with Japanese encephalitis virus core protein and participates in viral replication. Microbiol Immunol.

[94] Balinsky CA, Schmeisser H, Ganesan S, Singh K, Pierson TC, Zoon KC (2013). Nucleolin Interacts with the dengue virus capsid protein and plays a role in formation of infectious virus particles. J Virol.

[95] Taracena ML, Bottino-Rojas V, Talyuli OAC, Walter-Nuno AB, Oliveira JHM, Angleró-Rodriguez YI (2018). Regulation of midgut cell proliferation impacts *Aedes aegypti* susceptibility to dengue virus. PLoS Negl Trop Dis.

[96] Ličková M, Fumačová Havlíková S, Sláviková M, Slovák M, Drexler JF, Klempa B (2020). *Dermacentor reticulatus* is a vector of tick-borne encephalitis virus. Ticks Tick Borne Dis.

[97] Belova OA, Litov AG, Kholodilov IS, Kozlovskaya LI, Bell-Sakyi L, Romanova LI (2017). Properties of the tick-borne encephalitis virus population during persistent infection of ixodid ticks and tick cell lines. Ticks Tick Borne Dis.

