# Supplementary material for: Exploration of binary protein–protein interactions between tick-borne flaviviruses and Ixodes ricinus
Source: Parasit Vectors. 2021 Mar 6;14:144. doi: 10.1186/s13071-021-04651-3 (PMC7937244; doi:10.1186/s13071-021-04651-3)
Supplement: Supplementary file 6 — Additional file 6. Protein alignment of Ir1. [file 13071_2021_4651_MOESM6_ESM.pdf]

Protein alignment of Ir1

conserved hypothetical protein [Ixodes scapularis]

Sequence ID: EEC00213.1 **Length:** 260 **Number of Matches:** 1

Range 1: 82 to 147 GenPept Graphics ▼ Next Match ▲ Previous Match

Score	Expect	Method	Identities	Positives	Gaps	Frame
132 bits(332)	3e-40	Compositional matrix adjust.	65/66(98%)	66/66(100%)	0/66(0%)	+3

```
Query  3    AQGAVPVYLLDRENQSRAKVLSNMIKQKRKEKAGKWDVPVPKVRGVGDSEAFRVLKTGKR  182
            AQGAVPVYLLDRENQSRAKVLSNMIKQKRKEKAGKWDVPVPKVRGVGD+EAFRVLKTGKR
Sbjct  82   AQGAVPVYLLDRENQSRAKVLSNMIKQKRKEKAGKWDVPVPKVRGVGDAEAFRVLKTGKR  141

Query  183  KKKAWK  200
            KKKAWK
Sbjct  142  KKKAWK  147
```

intronic protein 259, isoform B [Drosophila melanogaster]

Sequence ID: NP_001260347.1 **Length:** 259 **Number of Matches:** 1

See 4 more title(s) ⌄ See all Identical Proteins(IPG)

Range 1: 83 to 146 GenPept Graphics ▼ Next Match ▲ Previous Match

Score	Expect	Method	Identities	Positives	Gaps	Frame
102 bits(253)	3e-28	Compositional matrix adjust.	50/64(78%)	55/64(85%)	0/64(0%)	+3

```
Query  9    GAVPVYLLDRENQSRAKVLSNMIKQKRKEKAGKWDVPVPKVRGVGDSEAFRVLKTGKRKK  188
            GA+P YLLDR  QS AKVLSNMIKQKRKEKAGKWDVP+PKVR   D+E F+VLKTGK K+
Sbjct  83   GALPHYLLDRGIQSSAKVLSNMIKQKRKEKAGKWDVPIPKVRAQSDAEVFKVLKTGKTKR  142

Query  189  KAWK  200
            KAWK
Sbjct  143  KAWK  146
```

ribosome biogenesis protein NSA2 homolog [Aedes aegypti]

Sequence ID: XP_021700401.1 **Length:** 259 **Number of Matches:** 1

See 3 more title(s) ⌄ See all Identical Proteins(IPG)

Range 1: 83 to 146 GenPept Graphics ▼ Next Match ▲ Previous Match

Score	Expect	Method	Identities	Positives	Gaps	Frame
102 bits(253)	2e-28	Compositional matrix adjust.	48/64(75%)	55/64(85%)	0/64(0%)	+3

```
Query  9    GAVPVYLLDRENQSRAKVLSNMIKQKRKEKAGKWDVPVPKVRGVGDSEAFRVLKTGKRKK  188
            GA+P YLLDR  QS AKVLSNMIKQKRKEKAGKWDVP+PKVR   DSE F+V+++GK K+
Sbjct  83   GAIPAYLLDRGVQSSAKVLSNMIKQKRKEKAGKWDVPIPKVRAQADSEVFKVIRSGKTKR  142

Query  189  KAWK  200
            KAWK
Sbjct  143  KAWK  146
```

ribosome biogenesis protein NSA2 homolog isoform 3 [Homo sapiens]

Sequence ID: NP_001351435.1 **Length:** 193 **Number of Matches:** 1

Range 1: 83 to 147 GenPept Graphics ▼ Next Match ▲ Previous Match

Score	Expect	Method	Identities	Positives	Gaps	Frame
110 bits(274)	2e-31	Compositional matrix adjust.	52/65(80%)	59/65(90%)	0/65(0%)	+3

```
Query  6    QGAVPVYLLDRENQSRAKVLSNMIKQKRKEKAGKWDVPVPKVRGVGDSEAFRVLKTGKRK  185
            QGAVP YLLDRE QSRAKVLSNMIKQKRKEKAGKW+VP+PKVR  G++E  +V++TGKRK
Sbjct  83   QGAVPAYLLDREGQSRAKVLSNMIKQKRKEKAGKWEVPLPKVRAQGETEVLKVIRTGKRK  142

Query  186  KKAWK  200
            KKAWK
Sbjct  143  KKAWK  147
```

Protein alignment of Ir2

lamin Dm0-like, partial [Ixodes scapularis]

Sequence ID: XP_029839273.1 **Length:** 561 **Number of Matches:** 1

Range 1: 279 to 548 GenPept Graphics ▼ Next Match ▲ Previous Match

Score	Expect	Method	Identities	Positives	Gaps	Frame
502 bits(1292)	4e-177	Compositional matrix adjust.	245/271(90%)	257/271(94%)	1/271(0%)	+1

```
Query  133  REQYESQMQLNREEMQDIYENKMRDLQSLLEQRSTDSSTKMEEMRAYKSRLEGLNSKISE  312
            RE YESQMQL+RE+ Q+IYENKMRDLQ LLEQ S+DSS+KMEEMRAYKSR+EGLNSKISE
Sbjct  279  RELYESQMQLDREKTQNIYENKMRDLQRLLEQSSSDSSSKMEEMRAYKSRIEGLNSKISE  338

Query  313  LESQNLSLTARVRDLERLLDQEREWHGRALMAKEEEISRLRTEIEDQLREYQDLLDIKVA  492
            LESQNLSLTARVRDLERLLDQEREWHGRALMAKEEE+SRLR EIEDQLREYQ+LLDIKVA
Sbjct  339  LESQNLSLTARVRDLERLLDQEREWHGRALMAKEEEVSRLRAEIEDQLREYQNLLDIKVA  398

Query  493  LDLEIAAYRKLLEGEESRLHITPSASTSGAEMYTSPLLRGTKRKRTFLSHREEQLNSDVQ  672
            LDLE AAYRKLLEGEESRLH TPSASTSGAEMYTSPLLRG KR+ TF SHREEQ N DVQ
Sbjct  399  LDLETAAYRKLLEGEESRLHFTPSASTSGAEMYTSPLLRGAKRRLTF-SHREEQSNFDVQ  457

Query  673  VSASAKGDLEISDHCPDGKYVIVKNKGTKEVPLAGWRIDRKSGDDEFTFRFHRTHVIQPG  852
            VSASAKGDLEISDHCPDGKYVIVKNKGTKE+PLAGWRIDR+SG+DEFTFRFHRTHVIQPG
Sbjct  458  VSASAKGDLEISDHCPDGKYVIVKNKGTKEIPLAGWRIDRRSGNDEFTFRFHRTHVIQPG  517

Query  853  TTITVWSSDAGATHSPPSDLVMRNQQWPHGE  945
            TTITVWSSDAGA HSPPSDLVMRNQQWPH E
Sbjct  518  TTITVWSSDAGAAHSPPSDLVMRNQQWPHVE  548
```

lamin, isoform B [Drosophila melanogaster]

Sequence ID: NP_001245892.1 **Length:** 622 **Number of Matches:** 1

See 9 more title(s) See all Identical Proteins(IPG)

Range 1: 270 to 553 GenPept Graphics ▼ Next Match ▲ Previous Match

Score	Expect	Method	Identities	Positives	Gaps	Frame
256 bits(654)	2e-80	Compositional matrix adjust.	131/284(46%)	191/284(67%)	15/284(5%)	+1

```
Query  127  QLREQYESQMQLNREEMQDIYENKMRDLQSLLEQRSTDSSTKMEEMRAYKSRLEGLNSKI  306
            +LR QYE QMQ+NR+E+Q +YE+K++ LQ    + S   +   +EE+R+ + R++ LN+ I
Sbjct  270  ELRAQYEEQMQINRDEIQSLYEDKIQRLQEAAARTSNSTHKSIEELRSTRVRIDALNANI  329

Query  307  SELESQNLSLTARVRDLERLLDQEREWHGRALMAKEEEISRLRTEIEDQLREYQDLLDIK  486
            +ELE   N  L AR+RDLER LD +RE HG+ +    E+E+ RLR E+   QL+EYQDL+DIK
Sbjct  330  NELEQANADLNARIRDLERQLDNDRERHGQEIDLLEKELIRLREEMTQQLKEYQDLMDIK  389

Query  487  VALDLEIAAYRKLLEGEESRLHITPSASTSGAEMYTSPLLRGT-------------KRKR  627
            V+LDLEIAAY KLL GEE+RL+ITP+ +T+   + ++   L    T             KRKR
Sbjct  390  VSLDLEIAAYDKLLVGEEARLNITPATNTATVQSFSQSLRNSTRATPSRRTPSAAVKRKR  449

Query  628  TFLSHREEQLNSDVQVSASAKGDLEISDHCPDGKYVIVKNKGTKEVPLAGWRIDRKSGDD  807
              +   E+   +D  VSASAKG++EI +   P+GK+V + NKG++EV + GW++ R    +
Sbjct  450  AVVDESEDHSVADYYVSASAKGNVEIKEIDPEGKFVRLFNKGSEEVAIGGWQLQRLINEK  509

Query  808  --EFTFRFHRTHVIQPGTTITVWSSDAGATHSPPSDLVMRNQQW  933
                T++FHR+  I+P   ITVWS+D  A+H PPS LVM++Q+W
Sbjct  510  GPSTTYKFHRSVRIEPNGVITVWSADTKASHEPPSSLVMKSQKW  553
```

LOW QUALITY PROTEIN: lamin Dm0 [Aedes aegypti]

Sequence ID: XP_021710074.1 **Length:** 579 **Number of Matches:** 1

Range 1: 249 to 520 GenPept Graphics ▼ Next Match ▲ Previous Match

Score	Expect	Method	Identities	Positives	Gaps	Frame
206 bits(523)	1e-61	Compositional matrix adjust.	118/286(41%)	170/286(59%)	28/286(9%)	+1

```
Query  130  LREQYESQMQLNREEMQDIYENKMRDLQSLLEQRSTDSSTKMEEMRAYKSRLEGLNSKIS  309
            L EQYE+++Q   ++++D Y+ +MR         + D  +++ E+  + S +  +
Sbjct  249  LSEQYEAKLQQTLQDLRDQYDGQMRA--------NRDEISELYEVSVFPSSIHTIL----  296

Query  310  ELESQNLSLTARVRDLERLLDQEREWHGRALMAKEEEISRLRTEIEDQLREYQDLLDIKV  489
             L     S  +R+RDLE  L+ ER+ H         ++EI RLR +I  QL+EYQDL+DIK+
Sbjct  297  -LTLLXPSFQSRLRDLELQLNAERQRHAEEKSKLQDEIERLRNQISQQLQEYQDLMDIKI  355

Query  490  ALDLEIAAYRKLLEGEESRLHITPSAST--------------SGAEMYTSPLLRGTKRKR  627
            +LD+EIAAY KLL  EE RL+ITP + T              SGA M  +P     KRKR
Sbjct  356  SLDMEIAAYDKLLSSEEVRLNITPGSQTTIFQTSSSSSSTARSGA-MRRTPQRAAAKRKR  414

Query  628  TFLSHREEQLNSDVQVSASAKGDLEISDHCPDGKYVIVKNKGTKEVPLAGWRIDRKSGDD  807
            T L   +E+  SD  V++SAKGD+EI +  P+GK+V + NK  KEV + GW + RK G +
Sbjct  415  TVLEESDERSVSDFSVTSSAKGDIEIFEVDPEGKFVKLHNKSAKEVQIGGWTLVRKVGTN  474

Query  808  EFTFRFHRTHVIQPGTTITVWSSDAGATHSPPSDLVMRNQQWPHGE  945
            E  F+FHRT  +  G+ +TVWSSD G TH PPS +VM+ Q+W  G+
Sbjct  475  ETVFKFHRTLKVDGGSFVTVWSSDLGQTHEPPSTIVMKGQKWFAGD  520
```

lamin-B1 isoform 2 [Homo sapiens]

Sequence ID: NP_001185486.1 **Length:** 376 **Number of Matches:** 1

Range 1: 38 to 315 GenPept Graphics ▼ Next Match ▲ Previous Match

Score	Expect	Method	Identities	Positives	Gaps	Frame
177 bits(448)	2e-51	Compositional matrix adjust.	100/279(36%)	178/279(63%)	7/279(2%)	+1

```
Query  127  QLREQYESQMQLNREEMQDIYENKMRDLQSLLEQRSTDSSTKMEEMRAYKSRLEGLNSKI  306
            ++REQ+++Q++L +EE++   Y  K+ + +    E  ++  ++  EE+    + R+E L+S++
Sbjct  38   EMREQHDAQVRLYKEELEQTYHAKLENARLSSEMNTSTVNSAREELMESRMRIESLSSQL  97

Query  307  SELESQNLSLTARVRDLERLLDQEREWHGRALMAKEEEISRLRTEIEDQLREYQDLLDIK  486
            S L+ ++ +   R+++LE LL +E++   R L  KE E++ +R +++ QL +Y+ LLD+K
Sbjct  98   SNLQKESRACLERIQELEDLLAKEKDNSRRMLTDKEREMAEIRDQMQQQLNDYEQLLDVK  157

Query  487  VALDLEIAAYRKLLEGEESRLHITPSAST-----SGAEMYTSPLLRGTKRKRTFLSHREE  651
            +ALD+EI+AYRKLLEGEE RL ++PS S+         +    +    RG KRKR  +   E
Sbjct  158  LALDMEISAYRKLLEGEEERLKLSPSPSSRVTVSRASSSRSVRTTRG-KRKRVDVEESEA  216

Query  652  QLNSDVQVSASAKGDLEISDHCPDGKYVIVKNKGTKEVPLAGWRIDRKSGDDEFTFRFHR  831
              +  +  SASA G++ I +   DGK++ +KN   ++ P+ GW + RK GD   ++++
Sbjct  217  SSSVSISHSASATGNVCIEEIDVDGKFIRLKNTSEQDQPMGGWEMIRKIGDTSVSYKYTS  276

Query  832  THVIQPGTTITVWSSDAGATHSPPSDLVMRNQQ-WPHGE  945
             +V++ G T+T+W+++AG T SPP+DL+ +NQ  W  GE
Sbjct  277  RYVLKAGQTVTIWAANAGVTASPPTDLIWKNQNSWGTGE  315
```

Protein alignment of Ir3

transcription factor jun-D [Ixodes scapularis]

Sequence ID: XP_002404571.2 **Length:** 291 **Number of Matches:** 1

See 1 more title(s) See all Identical Proteins(IPG)

Range 1: 1 to 235 GenPept Graphics ▼ Next Match ▲ Previous Match

Score	Expect	Method	Identities	Positives	Gaps	Frame
353 bits(905)	4e-122	Compositional matrix adjust.	223/238(94%)	224/238(94%)	5/238(2%)	+2

```
Query  278  MDTTFYEDIRGG--TRLAAGSqhaqpttqpqqhqqqhhpqqqqpqLVRRDDLSSRKRSM  451
            MDTTFYEDIRGG  +RLAAGSQHAQPTTQ QQ Q      Q Q  LVRRDDLSSRKRSM
Sbjct  1    MDTTFYEDIRGGGGSRLAAGSQHAQPTTQHQQQHHPQQQQPQ---LVRRDDLSSRKRSM  57

Query  452  TLDLNSAGRPERKAARFSSLLTSPDLNMLQLASPELERLIIAHNGLVtttptpttQYLFT  631
            TLDLNSAGRPERKAARFSSLLTSPDLNMLQLASPELERLIIAHNGLVTTTPTPTTQYLFT
Sbjct  58   TLDLNSAGRPERKAARFSSLLTSPDLNMLQLASPELERLIIAHNGLVTTTPTPTTQYLFT  117

Query  632  KTATEEQEQYARGFVDALAQLHQTAGGPSETalsaaeasdsgastssdsFILPSSSEHSL  811
            KTATEEQEQYARGFVDALAQLHQTAGGPSETALSAAEASDSGASTSSDSFILPSSSEHSL
Sbjct  118  KTATEEQEQYARGFVDALAQLHQTAGGPSETALSAAEASDSGASTSSDSFILPSSSEHSL  177

Query  812  GGGDGHVKDEPQTVPNLGATPPLSPIDMRDQERIKLERKRLRNRIAASKCRKRKLERI  985
            GGGDGHVKDEPQTVPNLGATPPLSPIDMRDQERIKLERKRLRNRIAASKCRKRKLERI
Sbjct  178  GGGDGHVKDEPQTVPNLGATPPLSPIDMRDQERIKLERKRLRNRIAASKCRKRKLERI  235
```

Djun gene product [Drosophila melanogaster]

Sequence ID: AAA28650.1 **Length:** 289 **Number of Matches:** 1

See 1 more title(s) See all Identical Proteins(IPG)

Range 1: 56 to 237 GenPept Graphics ▼ Next Match ▲ Previous Match

Score	Expect	Method	Identities	Positives	Gaps	Frame
87.0 bits(214)	3e-19	Compositional matrix adjust.	70/195(36%)	103/195(52%)	25/195(12%)	+2

```
Query  437  RKRSMTLDLNSAGRPERKAARFSSL-LTSPDLNMLQLASPELERLIIAHNGLVtttptpt  613
             KR  +LDLNS     ++   F+ L + SPDL+   + +P+LE++++++N +
Sbjct  56   NKRPGSLDLNSKSAKNKRI--FAPLVINSPDLSSKTVNTPDLEKILLSNNLM---QTPQP  110

Query  614  tQYLFTKT--ATEEQEQYARGFVDALAQLHQTAGG---------PSETalsaaeasdsga  760
             +   TK    T EQ  + RGF +AL  LH  +            +    +AA  +
Sbjct  111  GKVFPTKAGPVTVEQLDFGRGFEEALHNLHTNSQAFPSANSAANSAANNTTAAAMTAVNN  170

Query  761  stssdsFILPSSSEHSLGGGDGHVKDEPQTVPNLGATPPLSPIDMRDQERIKLERKRLRN  940
              S  +F   + +E     G   +KDEP    N  ++P ++PIDM  QE+IKLERKR RN
Sbjct  171  GISGGTFTYTNMTE-----GFSVIKDEPV---NQASSPTVNPIDMEAQEKIKLERKRQRN  222

Query  941  RIAASKCRKRKLERI  985
            R+AASKCRKRKLERI
Sbjct  223  RVAASKCRKRKLERI  237
```

transcription factor AP-1 isoform X2 [Aedes aegypti]

Sequence ID: XP_001663792.2 **Length:** 278 **Number of Matches:** 1

Range 1: 35 to 227 GenPept Graphics ▼ Next Match ▲ Previous Match

Score	Expect	Method	Identities	Positives	Gaps	Frame
119 bits(299)	2e-31	Compositional matrix adjust.	82/202(41%)	119/202(58%)	26/202(12%)	+2

```
Query  431  SSRKRSMTLDLNSAGRPERKAARFSS------LLTSPDLNMLQLASPELERLIIAHNGLV  592
            +S KR  TL+LN++   + + ARF++      ++TSPDL +L+L SPELE++II    L
Sbjct  35   NSLKRPATLELNAS---KSRKARFNASVTAPPVITSPDLQVLKLVSPELEKIIINSAAL-  90

Query  593  tttptpttQYLFTKTATEEQEQYARGFVDALAQLHQ-----TAGGPSETalsaaeasdsg  757
                   +  L+   AT EQEQ+A+GF +AL  + +          +    S    + +
Sbjct  91   --PTPTPSSILYPTKATTEQEQFAKGFDEALLSMREKDNINKMNNNNNNNKSINNNASTT  148

Query  758  astssdsFILPSSSEHSLGGGD------GHVKDEPQTVPNLGATPPLSPIDMRDQERIKL  919
             +  S      +++ +++ GGD      G +K+EPQ VP    + P+SPIDM +QERIKL
Sbjct  149  IAAISAISTATTTTHNTMSGGDITYTDLGVIKEEPQIVPQ---SSPMSPIDMENQERIKL  205

Query  920  ERKRLRNRIAASKCRKRKLERI  985
            ERKRLRNR+AASKCRKRKLERI
Sbjct  206  ERKRLRNRVAASKCRKRKLERI  227
```

JUN protein, partial [Homo sapiens]

Sequence ID: AAH09874.2 **Length:** 231 **Number of Matches:** 2

Range 1: 125 to 177 GenPept Graphics ▼ Next Match ▲ Previous Match

Score	Expect	Method	Identities	Positives	Gaps	Frame
90.1 bits(222)	6e-20	Compositional matrix adjust.	43/53(81%)	47/53(88%)	1/53(1%)	+2

```
Query  830  VKDEPQTVPNL-GATPPLSPIDMRDQERIKLERKRLRNRIAASKCRKRKLERI  985
            +K+EPQTVP + G TPPLSPIDM  QERIK ERKR+RNRIAASKCRKRKLERI
Sbjct  125  LKEEPQTVPEMPGETPPLSPIDMESQERIKAERKRMRNRIAASKCRKRKLERI  177
```

Range 2: 1 to 31 GenPept Graphics ▼ Next Match ▲ Previous Match ⮝ First Match

Score	Expect	Method	Identities	Positives	Gaps	Frame
34.3 bits(77)	1.0	Compositional matrix adjust.	17/31(55%)	20/31(64%)	0/31(0%)	+2

```
Query  632  KTATEEQEQYARGFVDALAQLHQTAGGPSET  724
            K  T+EQE +A GFV ALA+LH     PS T
Sbjct  1    KNVTDEQEGFAEGFVRALAELHSQNTLPSVT  31
```

Protein alignment of Ir4

TNF receptor-associated factor 4 isoform X2 [Ixodes scapularis]

Sequence ID: XP_029836788.1 **Length:** 386 **Number of Matches:** 1

Range 1: 16 to 315 GenPept Graphics ▼ Next Match ▲ Previous Match

Score	Expect	Method	Identities	Positives	Gaps	Frame
54.3 bits(129)	5e-08	Compositional matrix adjust.	70/310(23%)	112/310(36%)	92/310(29%)	+3

```
Query  213   KVEQMEWLSEDYYIYGTSIPEKIRCHGCRCVSNTIYPLENKCRGHGLCKDCKK--LDFTC  386
             KV+     L  D+  +    +P++ +C  C C+    IY L    C  HGLC DCK+  + F C
Sbjct  16    KVKGFIGLGVDFLRFPCQVPDRAKCKICDCIPERIYTL--LCPHHGLCSDCKEFFIGFYC  73

Query  387   YDHGGDVTADALRNAVGQTNYAAKRLWILCLFCGSNEAFLNLKDH-------MYKKHSE-  542
              +      T + L        N     RL +LC  C          L++H        + KK S+
Sbjct  74    RECDIHTTPEQLGALRSDYNLDGLRLKVLCPMCDKKLLLGELEEHNRSECPKIVKKCSDC  133

Query  543   ---ELAYMLKSSQKTDYEQ------------------------EKPASTEEKHRTSALT  638
                 L   L+S Q+ + ++                        EKP    ++
Sbjct  134   GVKVLNENLQSHQEVECQKRVVLCGSCKSAMSRDKLGVHDQVCPEKPVVCDD---CKGEV  190

Query  639   WRQDNFFQNADDTVEFVDDEAPACKHCKEEWDARDLGEHEKTCPKKDVQCRYCDEWIK--  812
              R+D      ++   +   V        C++C+   +        EH  K  C   K + C YC   ++K
Sbjct  191   LRKDESKHASECPMRVV-----CCENCEGSYAYSSEKEHRKQCEIKKLACEYCKLYLKGD  245

Query  813   -----------------------------QEELSDHKNVVH-------------TILDLQ  866
                                          ++E+  +H+    H               TI  +LQ
Sbjct  246   DEKKAHLKICEEASIECAFKDFGCKEKFPRKEMQEHEKDPHNALLNQVILKAMDTISELQ  305

Query  867   QKIKEMERCT  896
             QKIK+MERC
Sbjct  306   QKIKDMERCN  315
```

TNF receptor-associated factor 4 [Aedes aegypti]

Sequence ID: XP_001652160.1 **Length:** 481 **Number of Matches:** 1

See 1 more title(s) ⌄ See all Identical Proteins(IPG)

Range 1: 213 to 244 GenPept Graphics ▼ Next Match ▲ Previous Match

Score	Expect	Method	Identities	Positives	Gaps	Frame
32.3 bits(72)	0.63	Compositional matrix adjust.	14/32(44%)	17/32(53%)	1/32(3%)	+3

```
Query  708   CKHCKEEWDARDLGEHEKTCPKKDVQC-RYCD  800
             C HC   E+ A   L   H   TCP+   V C + CD
Sbjct  213   CPHCSREFSADTLSAHGATCPRSPVPCPQRCD  244
```

TNF receptor-associated factor 6 [Homo sapiens]

Sequence ID: AAH31052.1 **Length:** 522 **Number of Matches:** 1

Range 1: 188 to 233 GenPept Graphics ▼ Next Match ▲ Previous Match

Score	Expect	Method	Identities	Positives	Gaps	Frame
32.3 bits(72)	0.001	Compositional matrix adjust.	12/46(26%)	25/46(54%)	0/46(0%)	+3

```
Query  705   ACKHCKEEWDARDLGEHEKTCPKKDVQCRYCDEWIKQEELSDHKNV  842
             +C +C          D    H++ CP   +V  C YC+   +  +E++  +H ++
Sbjct  188   SCDNCAASMAFEDKEIHDQNCPLANVICEYCNTILIREQMPNHYDL  233
```

Protein alignment of Ir5

dynein light chain 1, cytoplasmic [Ixodes scapularis]

Sequence ID: XP_002408929.1 **Length:** 89 **Number of Matches:** 1

See 1 more title(s) See all Identical Proteins(IPG)

Range 1: 1 to 83 GenPept Graphics ▼ Next Match ▲ Previous Match

Score	Expect	Method	Identities	Positives	Gaps	Frame
175 bits(444)	5e-57	Compositional matrix adjust.	83/83(100%)	83/83(100%)	0/83(0%)	+3

```
Query  165   MSDKKAVIKNADMSEEMQQDAVDVATQALEKYNIEKDIAAYIKKEFDKKYNPTWHCIVGR  344
             MSDKKAVIKNADMSEEMQQDAVDVATQALEKYNIEKDIAAYIKKEFDKKYNPTWHCIVGR
Sbjct  1     MSDKKAVIKNADMSEEMQQDAVDVATQALEKYNIEKDIAAYIKKEFDKKYNPTWHCIVGR  60

Query  345   NFGSYVTHETKHFIYFYLGQVAI  413
             NFGSYVTHETKHFIYFYLGQVAI
Sbjct  61    NFGSYVTHETKHFIYFYLGQVAI  83
```

cut up, isoform F [Drosophila melanogaster]

Sequence ID: NP_001356933.1 **Length:** 267 **Number of Matches:** 1

See 1 more title(s) See all Identical Proteins(IPG)

Range 1: 1 to 83 GenPept Graphics ▼ Next Match ▲ Previous Match

Score	Expect	Method	Identities	Positives	Gaps	Frame
179 bits(455)	4e-56	Compositional matrix adjust.	80/83(96%)	82/83(98%)	0/83(0%)	+3

```
Query  165   MSDKKAVIKNADMSEEMQQDAVDVATQALEKYNIEKDIAAYIKKEFDKKYNPTWHCIVGR  344
             MSD+KAVIKNADMSEEMQQDAVD ATQALEKYNIEKDIAAYIKKEFDKKYNPTWHCIVGR
Sbjct  1     MSDRKAVIKNADMSEEMQQDAVDCATQALEKYNIEKDIAAYIKKEFDKKYNPTWHCIVGR  60

Query  345   NFGSYVTHETKHFIYFYLGQVAI  413
             NFGSYVTHET+HFIYFYLGQVAI
Sbjct  61    NFGSYVTHETRHFIYFYLGQVAI  83
```

dynein light chain 1, cytoplasmic isoform X1 [Aedes aegypti]

Sequence ID: XP_021709064.1 **Length:** 128 **Number of Matches:** 1

See 3 more title(s) See all Identical Proteins(IPG)

Range 1: 37 to 122 GenPept Graphics ▼ Next Match ▲ Previous Match

Score	Expect	Method	Identities	Positives	Gaps	Frame
172 bits(436)	4e-55	Compositional matrix adjust.	79/86(92%)	83/86(96%)	0/86(0%)	+3

```
Query  156   SLRMSDKKAVIKNADMSEEMQQDAVDVATQALEKYNIEKDIAAYIKKEFDKKYNPTWHCI  335
             S  +MSD+KAVIKNADM EEMQQDAVD ATQALEKYNIEKDIAAYIKKEFDK+YNPTWHCI
Sbjct  37    SSKMSDRKAVIKNADMGEEMQQDAVDCATQALEKYNIEKDIAAYIKKEFDKRYNPTWHCI  96

Query  336   VGRNFGSYVTHETKHFIYFYLGQVAI  413
             VGRNFGSYVTHET+HFIYFYLGQVAI
Sbjct  97    VGRNFGSYVTHETRHFIYFYLGQVAI  122
```

dynein light chain 2, cytoplasmic [Homo sapiens]

Sequence ID: NP_542408.1 **Length:** 89 **Number of Matches:** 1

See 5 more title(s) See all Identical Proteins(IPG)

Range 1: 1 to 83 GenPept Graphics ▼ Next Match ▲ Previous Match

Score	Expect	Method	Identities	Positives	Gaps	Frame
170 bits(431)	3e-54	Compositional matrix adjust.	79/83(95%)	82/83(98%)	0/83(0%)	+3

```
Query  165   MSDKKAVIKNADMSEEMQQDAVDVATQALEKYNIEKDIAAYIKKEFDKKYNPTWHCIVGR  344
             MSD+KAVIKNADMSE+MQQDAVD ATQA+EKYNIEKDIAAYIKKEFDKKYNPTWHCIVGR
Sbjct  1     MSDRKAVIKNADMSEDMQQDAVDCATQAMEKYNIEKDIAAYIKKEFDKKYNPTWHCIVGR  60

Query  345   NFGSYVTHETKHFIYFYLGQVAI  413
             NFGSYVTHETKHFIYFYLGQVAI
Sbjct  61    NFGSYVTHETKHFIYFYLGQVAI  83
```

Protein alignment of Ir6

LOW QUALITY PROTEIN: uncharacterized protein LOC8050906 [Ixodes scapularis]

Sequence ID: XP_002434995.2 Length: **331** Number of Matches: **1**

Range 1: 78 to 323 GenPept Graphics ▼ Next Match ▲ Previous Match

Score	Expect	Method	Identities	Positives	Gaps	Frame
337 bits(864)	3e-115	Compositional matrix adjust.	201/252(80%)	217/252(86%)	11/252(4%)	+1

```
Query  103   TSLYKKVGGRPTYQNREALLTHARRHKHSLPFELKRGDVCRRKQASSQAAQHIIVPGAQ  282
             TS + + G   TYQNREALLTHARRHKHSLPFELKRGDVCRRKQASSQAAQHIIVPGAQ
Sbjct  78    TSWHCQCGA--TYQNREALLTHARRHKHSLPFELKRGDVCRRKQASSQAAQHIIVPGAQ  135

Query  283   VIIVIQDQKTSSGQKSQDIPLHGANRTTGQWRNIVPKassipmvmpvpsvpplpsavaga  462
             VIIVIQDQKTSSGQK QDIP+HGANRTTGQWRNIVPKASS+P+VMPVPSVPPLPSA+AGA
Sbjct  136   VIIVIQDQKTSSGQKGQDIPVHGANRTTGQWRNIVPKASSVPIVMPVPSVPPLPSAMAGA  195

Query  463   TLPSGSTICEGKVHQASQTNVSCKSKRTKDSSIQASTTTSCMRDASELVDLPPKACRRHR  642
             +L SG+TICEGKVHQASQTNVSCKSKRTKDSSIQASTTTSCMRDASELVDLPPK   + +
Sbjct  196   SLSSGNTICEGKVHQASQTNVSCKSKRTKDSSIQASTTTSCMRDASELVDLPPKXVSKTQ  255

Query  643   AMKTAVQTQTSGFDV---QCKGNQPNL--SRNGSCHKKASHGCSIETQTVESAVCKAKKT  807
             + + +    +S  D+    C     NL     GSCHKKASHGCSIETQT+ESAVCKAKKT
Sbjct  256   SHEDS----SSDADIWXSTCSARGTNLICPEXGSCHKKASHGCSIETQTIESAVCKAKKT  311

Query  808   RARRTSSKQPPK  843
             RARRT SKQPPK
Sbjct  312   RARRTPSKQPPK  323
```

Protein alignment of Ir7

ubiquilin-1 [Ixodes scapularis]

Sequence ID: XP_029826161.1 **Length:** 570 **Number of Matches:** 1

Range 1: 236 to 451 GenPept Graphics ▼ Next Match ▲ Previous Match

Score	Expect	Method	Identities	Positives	Gaps	Frame
221 bits(563)	7e-68	Compositional matrix adjust.	216/216(100%)	216/216(100%)	0/216(0%)	+2

```
Query  128   RRMYTELQEPMMNAAQEQFGGNPFASLLNaggagspgappqpqqeaggqspsNQGTENRN   307
             RRMYTELQEPMMNAAQEQFGGNPFASLLNAGGAGSPGAPPQPQQEAGGQSPSNQGTENRN
Sbjct  236   RRMYTELQEPMMNAAQEQFGGNPFASLLNAGGAGSPGAPPQPQQEAGGQSPSNQGTENRN   295

Query  308   PLpnpwapgsggspagngtggqappaggvlgglgglggpgtggagmfgtpgMQSVMRQLT   487
             PLPNPWAPGSGGSPAGNGTGGQAPPAGGVLGGLGGLGGPGTGGAGMFGTPGMQSVMRQLT
Sbjct  296   PLPNPWAPGSGGSPAGNGTGGQAPPAGGVLGGLGGLGGPGTGGAGMFGTPGMQSVMRQLT   355

Query  488   EDPSLMQNMMNAPYVQNMLQALAANPEMANQVMASNPLLAGNPQLQEQMRRLMPQFLQQL   667
             EDPSLMQNMMNAPYVQNMLQALAANPEMANQVMASNPLLAGNPQLQEQMRRLMPQFLQQL
Sbjct  356   EDPSLMQNMMNAPYVQNMLQALAANPEMANQVMASNPLLAGNPQLQEQMRRLMPQFLQQL   415

Query  668   QNPEVQGLITNPqamqammqiqqgmeqLHRVAPSVF   775
             QNPEVQGLITNPQAMQAMMQIQQGMEQLHRVAPSVF
Sbjct  416   QNPEVQGLITNPQAMQAMMQIQQGMEQLHRVAPSVF   451
```

ubiquilin, isoform B [Drosophila melanogaster]

Sequence ID: NP_001285457.1 **Length:** 547 **Number of Matches:** 2

See 4 more title(s) ⌄ See all Identical Proteins(IPG)

Range 1: 312 to 416 GenPept Graphics ▼ Next Match ▲ Previous Match

Score	Expect	Method	Identities	Positives	Gaps	Frame
94.0 bits(232)	4e-21	Compositional matrix adjust.	52/105(50%)	89/105(84%)	0/105(0%)	+2

```
Query  461   MQSVMRQLTEDPSLMQNMMNAPYVQNMLQALAANPEMANQVMASNPLLAGNPQLQEQMRR   640
             M+S+++Q+ ++P++MQN++NAPY ++M+++++ +P+MA ++++S+PL++ NP LQEQ+R+
Sbjct  312   MRSLLQQMADNPAMMQNLLNAPYTRSMMESMSQDPDMAARLLSSSPLMSNNPALQEQVRQ   371

Query  641   LMPQFLQQLQNPEVQGLITNPqamqammqiqqgmeqLHRVAPSVF   775
             +MPQF+ Q+QNPEV  ++TNP AM A++QIQQGMEQL   AP +
Sbjct  372   MMPQFMAQMQNPEVMNMLTNPDAMNAILQIQQGMEQLRSAAPGLV   416
```

Range 2: 226 to 274 GenPept Graphics ▼ Next Match ▲ Previous Match ⏶ First Match

Score	Expect	Method	Identities	Positives	Gaps	Frame
47.8 bits(112)	9e-06	Compositional matrix adjust.	31/62(50%)	39/62(62%)	13/62(20%)	+2

```
Query  128   RRMYTELQEPMMNAAQEQFGGNPFASLLNaggagspgappqpqqeaggqspsNQGTENRN   307
             +R+Y ++QEPMMNAA E FG NPFA L++ GG+G+   P              QGTENRN
Sbjct  226   QRIYRDIQEPMMNAATESFGRNPFAGLVDGGGSGAGNNPQ-------------QGTENRN   272

Query  308   PL   313
             PL
Sbjct  273   PL   274
```

ubiquilin-1 [Aedes aegypti]

Sequence ID: XP_001652587.1 **Length:** 505 **Number of Matches:** 1

See 1 more title(s) ⌄ See all Identical Proteins(IPG)

Range 1: 296 to 400 GenPept Graphics ▼ Next Match ▲ Previous Match

Score	Expect	Method	Identities	Positives	Gaps	Frame
110 bits(274)	7e-27	Compositional matrix adjust.	67/105(64%)	90/105(85%)	0/105(0%)	+2

```
Query  461   MQSVMRQLTEDPSLMQNMMNAPYVQNMLQALAANPEMANQVMASNPLLAGNPQLQEQMRR   640
             MQS+++Q+ ++PSLM NMM+APY +NML+AL+A+P MA  +M+ NPLLA NP LQEQMR
Sbjct  296   MQSLLQQMGDNPSLMSNMMSAPYTRNMLEALSADPNMAANLMSQNPLLANNPALQEQMRT   355

Query  641   LMPQFLQQLQNPEVQGLITNPqamqammqiqqgmeqLHRVAPSVF   775
             +MPQFLQQLQNPE+Q +++NPQA++A++QIQQGMEQL  VAP +
Sbjct  356   MMPQFLQQLQNPEMQQMMSNPQAIEAILQIQQGMEQLRSVAPGLV   400
```

ubiquilin-1 isoform 1 [Homo sapiens]

Sequence ID: NP_038466.2 **Length:** 589 **Number of Matches:** 2

See 5 more title(s) ⌄ See all Identical Proteins(IPG)

Range 1: 377 to 457 GenPept Graphics ▼ Next Match ▲ Previous Match

Score	Expect	Method	Identities	Positives	Gaps	Frame
101 bits(252)	4e-26	Compositional matrix adjust.	47/81(58%)	70/81(86%)	0/81(0%)	+2

```
Query  461   MQSVMRQLTEDPSLMQNMMNAPYVQNMLQALAANPEMANQVMASNPLLAGNPQLQEQMRR   640
             MQS+++Q+TE+P LMQNM++APY+++M+Q+L+ NP++A Q+M +NPL AGNPQLQEQMR+
Sbjct  377   MQSLLQQITENPQLMQNMLSAPYMRSMMQSLSQNPDLAAQMMLNNPLFAGNPQLQEQMRQ   436

Query  641   LMPQFLQQLQNPEVQGLITNP   703
              +P FLQQ+QNP+    ++NP
Sbjct  437   QLPTFLQQMQNPDTLSAMSNP   457
```

Range 2: 273 to 301 GenPept Graphics ▼ Next Match ▲ Previous Match ⏶ First Match

Score	Expect	Method	Identities	Positives	Gaps	Frame
55.8 bits(133)	8e-11	Compositional matrix adjust.	23/29(79%)	29/29(100%)	0/29(0%)	+2

```
Query  128   RRMYTELQEPMMNAAQEQFGGNPFASLLN   214
             RRMYT++QEPM++AAQEQFGGNPFASL++
Sbjct  273   RRMYTDIQEPMLSAAQEQFGGNPFASLVS   301
```

Protein alignment of Ir8

small glutamine-rich tetratricopeptide repeat-containing protein beta-like [Ixodes scapularis]

Sequence ID: XP_029827243.1 Length: 324 Number of Matches: 2

See 2 more title(s) See all Identical Proteins(IPG)

Range 1: 5 to 238 GenPept Graphics ▼ Next Match ▲ Previous Match

Score	Expect	Method	Identities	Positives	Gaps	Frame
417 bits(1071)	5e-147	Compositional matrix adjust.	232/234(99%)	234/234(100%)	0/234(0%)	+2

```
Query  200   RRLVLSIVQFLRQQLQTADLSSDAKESVEVAVQCLETAYGVSIDDLSNDSLLVSRTLLDI  379
             +RLVLSIVQFLRQQLQTADLSSDAKESVEVAVQCLETAYGVSIDDLSNDSLLVSRTLLDI
Sbjct  5     KRLVLSIVQFLRQQLQTADLSSDAKESVEVAVQCLETAYGVSIDDLSNDSLLVSRTLLDI  64

Query  380   FRevvvqehaqvheTLPEPTEAQRAEAEKYKQEGNNMMKLEMYTAALECYTKAISLDGNN  559
             FREVVVQEHAQVHETLPEPTEAQRAEAEKYKQEGNNMMKLEMYTAALECYTKAISLDGNN
Sbjct  65    FREVVVQEHAQVHETLPEPTEAQRAEAEKYKQEGNNMMKLEMYTAALECYTKAISLDGNN  124

Query  560   AVYYCNRAAAHSKLNNHANAIEDCQRALDIDPKYGKAYGRIGLAYASLNEHQRAKECYQK  739
             AVYYCNRAAAHSKLNNHA+AIEDCQRALDIDPKYGKAYGRIGLAYASLNEHQRAKECYQK
Sbjct  125   AVYYCNRAAAHSKLNNHADAIEDCQRALDIDPKYGKAYGRIGLAYASLNEHQRAKECYQK  184

Query  740   AVELDPENQSYINNLRVAEEKLRGMPSPgngdarraggvgggaPMDFGSLLNNP  901
             AVELDPENQSYINNLRVAEEKLRGMPSPGNGDARRAGGVGGGAPMDFGSLLNNP
Sbjct  185   AVELDPENQSYINNLRVAEEKLRGMPSPGNGDARRAGGVGGGAPMDFGSLLNNP  238
```

Range 2: 238 to 251 GenPept Graphics ▼ Next Match ▲ Previous Match First Match

Score	Expect	Method	Identities	Positives	Gaps	Frame
33.5 bits(75)	0.26	Compositional matrix adjust.	14/14(100%)	14/14(100%)	0/14(0%)	+1

```
Query  898   PTLMNMAATLMQDP  939
             PTLMNMAATLMQDP
Sbjct  238   PTLMNMAATLMQDP  251
```

small glutamine-rich tetratricopeptide containing protein, isoform B [Drosophila melanogaster]

Sequence ID: NP_001246058.1 Length: 331 Number of Matches: 1

See 4 more title(s) See all Identical Proteins(IPG)

Range 1: 7 to 227 GenPept Graphics ▼ Next Match ▲ Previous Match

Score	Expect	Method	Identities	Positives	Gaps	Frame
113 bits(282)	1e-28	Compositional matrix adjust.	73/232(31%)	116/232(50%)	45/232(19%)	+2

```
Query  200   RRLVLSIVQFLRQQLQTADLSSDAKESVEVAVQCLETAYGVSIDDLS-------------  340
             +  V S + +L++Q     +S D  ES+EVA+QCL+ A+ +  DD+
Sbjct  7     QSFVRSFIDYLKKQGDV--MSPDQTESIEVAIQCLQAAFDLG-DDVEAAPAAAGEEQATT  63

Query  341   ----------NDSLLVSRTL-----------LDIFRevvvqehaqvheTLPEPTEAQRAE  457
                       +D++                 +D+F         +  E+L        A
Sbjct  64    QSSSTASAPDDDAVASGSAGIGAAAAAVPNNIDMFELFQSLYTERNPESL--------AL  115

Query  458   AEKYKQEGNNMMKLEMYTAALECYTKAISLDGNNAVYYCNRAAAHSKLNNHANAIEDCQR  637
             AE  K EGN +MK   Y  AL  Y +AI+ D  N ++YCNRAAAH +L  +  A+ DC+
Sbjct  116   AESIKNEGNRLMKENKYNEALLQYNRAIAFDPKNPIFYCNRAAAHIRLGENERAVTDCKS  175

Query  638   ALDIDPKYGKAYGRIGLAYASLNEHQRAKECYQKAVELDPENQSYINNLRVA  793
             AL  +  Y KAY R+G+AY+++   ++A++ Y KA+EL+P+N+ Y +NL  A
Sbjct  176   ALVYNNNYSKAYCRLGVAYSNMGNFEKAEQAYAKAIELEPDNEVYKSNLEAA  227
```

small glutamine-rich tetratricopeptide repeat-containing protein beta [Aedes aegypti]

Sequence ID: XP_001657084.1 Length: 327 Number of Matches: 1

See 2 more title(s) See all Identical Proteins(IPG)

Range 1: 4 to 205 GenPept Graphics ▼ Next Match ▲ Previous Match

Score	Expect	Method	Identities	Positives	Gaps	Frame
172 bits(436)	4e-51	Compositional matrix adjust.	88/213(41%)	133/213(62%)	14/213(6%)	+2

```
Query  191   VGGRRLVLSIVQFLRQQLQTADLSSDAKESVEVAVQCLETAYGVSID---DLSNDSLLVS  361
             +  +  V S ++FL  QL+  + SSD++ES+EVA+QCLE  Y +  +   D S ++ L
Sbjct  4     IEAKFFVRSFIRFLNGQLEQPNFSSDSRESLEVAIQCLENVYEIGQEAEGDNSQENPLNH  63

Query  362   RTLLDIFRevvvqehaqvheTLPEPTEAQRAEAEKYKQEGNNMMKLEMYTAALECYTKAI  541
               L +++R            T    T  ++ EAE  K EGN +MK E Y  AL  Y+KAI
Sbjct  64    IDLFEVYRS-----------TFTNVTPERKQEAENLKNEGNRLMKEEKYQEALNTYSKAI  112

Query  542   SLDGNNAVYYCNRAAAHSKLNNHANAIEDCQRALDIDPKYGKAYGRIGLAYASLNEHQRA  721
             SLD  N V+YCNRAAA+S+L ++  A +DC+ +L  DP Y KAYGR+GLAY+ +N+H++A
Sbjct  113   SLDATNPVFYCNRAAAYSRLGDYQAAADDCRMSLRYDPNYSKAYGRLGLAYSKMNKHEQA  172

Query  722   KECYQKAVELDPENQSYINNLRVAEEKLRGMPS  820
              + YQ A+ ++P+NQ Y NN+ V +++L  + S
Sbjct  173   LDAYQNALRIEPDNQDYKNNMGVTQQRLEELRS  205
```

small glutamine-rich tetratricopeptide repeat-containing protein alpha [Homo sapiens]

Sequence ID: NP_003012.1 Length: 313 Number of Matches: 1

See 5 more title(s) See all Identical Proteins(IPG)

Range 1: 5 to 228 GenPept Graphics ▼ Next Match ▲ Previous Match

Score	Expect	Method	Identities	Positives	Gaps	Frame
216 bits(551)	1e-67	Compositional matrix adjust.	116/237(49%)	155/237(65%)	16/237(6%)	+2

```
Query  200   RRLVLSIVQFLRQQLQTADLSSDAKESVEVAVQCLETAYGVSIDDLSNDSLLVSRTLLDI  379
             +RL  +I+QFL  QL+   LSSDA+ES+EVA+QCLETA+GV+++D     L + +TL +I
Sbjct  5     KRLAYAIIQFLHDQLRHGGLSSDAQESLEVAIQCLETAFGVTVED---SDLALPQTLPEI  61

Query  380   FRevvvqehaqvheTLP---EPTEAQRAEAEKYKQEGNNMMKLEMYTAALECYTKAISLD  550
             F      +        P    P+E   AEAE+ K EGN  MK+E + AA+  Y KAI L+
Sbjct  62    FEAAATGKEMPQDLRSPARTPPSEEDSAEAERLKTEGNEQMKVENFEAAVHFYGKAIELN  121

Query  551   GNNAVYYCNRAAAHSKLNNHANAIEDCQRALDIDPKYGKAYGRIGLAYASLNEHQRAKEC  730
               NAVY+CNRAAA+SKL N+A A++DC+RA+ IDP Y KAYGR+GLA +SLN+H  A
Sbjct  122   PANAVYFCNRAAAYSKLGNYAGAVQDCERAICIDPAYSKAYGRMGLALSSLNKHVEAVAY  181

Query  731   YQKAVELDPENQSYINNLRVAEEKLRGMPSPgngdarraggvgggaPMDFGSLLNNP  901
             Y+KA+ELDP+N++Y +NL++AE KLR  PSP  G              D   LLNNP
Sbjct  182   YKKALELDPDNETYKSNLKIAELKLREAPSPTGGVGS----------FDIAGLLNNP  228
```

Protein alignment of Ir9

TNF receptor-associated factor 6-like [Ixodes scapularis]

Sequence ID: XP_029846366.1 **Length:** 449 **Number of Matches:** 1

Range 1: 1 to 205 GenPept Graphics ▼ Next Match ▲ Previous Match

Score	Expect	Method	Identities	Positives	Gaps	Frame
385 bits(990)	9e-134	Compositional matrix adjust.	182/205(89%)	191/205(93%)	0/205(0%)	+2

```
Query  194  MRSIFISGFSDTLDWRPLYFQESSVAQSACSLCGLVSRKIVRLPCDHALCAECHEESQRR  373
            MRS FISGFSDTLDWRPLYFQESSVA SACSLCGL  R +VRLPCDH LC+ECH+ESQRR
Sbjct  1    MRSTFISGFSDTLDWRPLYFQESSVAHSACSLCGLCWRNVVRLPCDHILCSECHQESQRR  60

Query  374  GSTCPLDKESFAKENIVHLDISEGYILKRTVACGNAPNGCDFIGQASRLVDHYKQCSFHV  553
            GSTCPLDKESF  +NIVHLDISEGYILKRTVACGNAPNGCDFIGQASRLVDHYKQCSFHV
Sbjct  61   GSTCPLDKESFGDDNIVHLDISEGYILKRTVACGNAPNGCDFIGQASRLVDHYKQCSFHV  120

Query  554  VPCPRCKSSVLRTELVGHCKDGCSSESTTPVPIPNYINVNYDNLEITSSELKREMLKISE  733
            VPCPRC+SSVLRTELVGHCKDGCSS STTPVPIP YINVNYD+LEITSSELKREM KISE
Sbjct  121  VPCPRCQSSVLRTELVGHCKDGCSSASTTPVPIPYYINVNYDHLEITSSELKREMFKISE  180

Query  734  DLSSLQTSLNQWFEEVRTLEKSTSK  808
            +LS LQT LNQWFEEVRT EK+T+K
Sbjct  181  NLSCLQTGLNQWFEEVRTFEKNTNK  205
```

early girl, isoform B [Drosophila melanogaster]

Sequence ID: NP_001261904.1 **Length:** 315 **Number of Matches:** 1

See 4 more title(s) ⌄ See all Identical Proteins(IPG)

Range 1: 8 to 183 GenPept Graphics ▼ Next Match ▲ Previous Match

Score	Expect	Method	Identities	Positives	Gaps	Frame
52.4 bits(124)	2e-07	Compositional matrix adjust.	43/179(24%)	77/179(43%)	13/179(7%)	+2

```
Query  251  FQESSVAQSACSLC-GLVSRKIVRLPCDHALCAECHEESQRRGSTCPLDKESFAKENIVH  427
            FQ     +  C +C G++   +  + C+HA C  C  E   R  TCP+D+ S    N+
Sbjct  8    FQGEVDEELTCPICSGVLEDPLQAVMCEHAFCRGCINEWLTRQPTCPVDRNSLTTANLRA  67

Query  428  L-DISEGYILKRTVACGNAPNGCDFIGQASRLVDHYKQCSFH---VVPCPR-CKSSVLRT  592
            +  I    + + ++ C NAP GC  + +      H  +C  +     PC + C   + +
Sbjct  68   VPRILRNLLSRLSITCDNAPYGCTAVLKLDAYNSHLDECIHNPKRPFPCEKGCGFDIPKD  127

Query  593  ELVGHCKDGCSSESTTPV----PIPNYINVNYDNLEITSSELKREMLKISEDLSSLQTS  757
            EL  H    C  E  T +         +     + ++T +ELKRE+    + + +++ S
Sbjct  128  ELKDH---NCVRELRTLIVKQTEKMGELKSELTDQQLTINELKRELQLFKDFMRAMRVS  183
```

E3 ubiquitin-protein ligase NRDP1 [Aedes aegypti]

Sequence ID: XP_001658984.1 **Length:** 315 **Number of Matches:** 1

See 5 more title(s) ⌄ See all Identical Proteins(IPG)

Range 1: 8 to 183 GenPept Graphics ▼ Next Match ▲ Previous Match

Score	Expect	Method	Identities	Positives	Gaps	Frame
49.7 bits(117)	1e-06	Compositional matrix adjust.	43/183(23%)	79/183(43%)	21/183(11%)	+2

```
Query  251  FQESSVAQSACSLC-GLVSRKIVRLPCDHALCAECHEESQRRGSTCPLDKESFAKENIVH  427
            FQ     +  C +C G++   +  + C+HA C  C  E   R  TCP+D+      N+
Sbjct  8    FQGDVDEELICPICSGVLEEPLQAVACEHAFCRACITEWLSRQPTCPVDRNPITNSNLRA  67

Query  428  L-DISEGYILKRTVACGNAPNGCDFIGQASRLVDHYKQCSFH---VVPCPR-CKSSVLRT  592
            +  I    + +  ++C NA  GC  + +   L  H ++C  +    +PC + C   + +
Sbjct  68   VPRILRNLLSRLNISCENAIYGCTLVLKLDTLATHIEECEHNPKRPLPCEKGCGFVIPKD  127

Query  593  ELVGHCKDGCSSESTTPV--------PIPNYINVNYDNLEITSSELKREMLKISEDLSSL  748
            E   H    C  E  + V         + N IN    +  +  +ELKRE+  + + + ++
Sbjct  128  EYKDH---NCFRELRSLVHNQQQKMSELKNEIN----DQNLVINELKRELNLVKDFMRAM  180

Query  749  QTS  757
            + S
Sbjct  181  RVS  183
```

RING finger protein 151 isoform X1 [Homo sapiens]

Sequence ID: XP_005255186.1 **Length:** 254 **Number of Matches:** 1

Range 1: 29 to 153 GenPept Graphics ▼ Next Match ▲ Previous Match

Score	Expect	Method	Identities	Positives	Gaps	Frame
59.7 bits(143)	2e-09	Compositional matrix adjust.	38/126(30%)	54/126(42%)	3/126(2%)	+2

```
Query  281  CSLCGLVSRKIVRLPCDHALCAECHEESQRRGSTCPLDKESFAKENIVHLDISEGYILKR  460
            CS+C  V ++  RLPC H  C +C      R  TCP  ++   ++ +VH++     I +
Sbjct  29   CSVCHGVLKRPARLPCSHIFCKKCILRWLARQKTCPCCRKEVKRKKVVHMNKLRKTIGRL  88

Query  461  TVACGNAPNGCDFIGQASRLVDHYKQCSFHVVPCPR--CKSSVLRTELVGHCKDGCSSES  634
             V C NA  GC      +    H   C F +  CP   C S V R  L  H +  C   S
Sbjct  89   EVKCKNADAGCIVTCPLAHRKGHQDSCPFELTACPNEGCTSQVPRGTLAEH-RQHCQQGS  147

Query  635  TTPVPI  652
                P+
Sbjct  148  QQRCPL  153
```

Protein alignment of Ir10

serine/threonine-protein kinase 26 isoform X1 [Ixodes scapularis]

Sequence ID: XP_029827734.1 Length: 582 Number of Matches: 1

Range 1: 13 to 234 GenPept Graphics ▼ Next Match ▲ Previous Match

Score	Expect	Method	Identities	Positives	Gaps	Frame
400 bits(1027)	9e-137	Compositional matrix adjust.	217/222(98%)	219/222(98%)	0/222(0%)	+3

```
Query  270  ALKMKVDPELIFTKQERIGKGSFGEVFKGMDNRTQQVVaikiidleeaedeiediqqeiM  449
            A + KVDPELIFTKQERIGKGSFGEVFKGMDNRTQQVVAIKIIDLEEAEDEIEDIQQEIM
Sbjct  13   ASQFKVDPELIFTKQERIGKGSFGEVFKGMDNRTQQVVAIKIIDLEEAEDEIEDIQQEIM  72

Query  450  VLSQCDSPWVTKYYGSYLKGTKLWIIMEYLGGGSALDLMKAGRFEELHIAVILREVLKGL  629
            VLSQCDSPWVTKYYGSYLKGTKLWIIMEYLGGGSALDLMKAGRFEELHIAVILREVLKGL
Sbjct  73   VLSQCDSPWVTKYYGSYLKGTKLWIIMEYLGGGSALDLMKAGRFEELHIAVILREVLKGL  132

Query  630  DYLHCERKLHRDIKAANVLLSEMGDVKLADFGVAGQLTNTTSKRNTFVGTPFWMAPEVIK  809
            DYLHCERKLHRDIKAANVLLSEMGDVKLADFGVAGQLTNTTSKRNTFVGTPFWMAPEVIK
Sbjct  133  DYLHCERKLHRDIKAANVLLSEMGDVKLADFGVAGQLTNTTSKRNTFVGTPFWMAPEVIK  192

Query  810  QSAYDSKADIWSLGITAIELAKGEPPNSDLHPMRVLFLIPRT  935
            QSAYDSKADIWSLGITAIELAKGEPPNSDLHPMRVLFLIP+
Sbjct  193  QSAYDSKADIWSLGITAIELAKGEPPNSDLHPMRVLFLIPKN  234
```

germinal centre kinase III [Drosophila melanogaster]

Sequence ID: NP_650596.1 Length: 642 Number of Matches: 1

See 2 more title(s) See all Identical Proteins(IPG)

Range 1: 6 to 223 GenPept Graphics ▼ Next Match ▲ Previous Match

Score	Expect	Method	Identities	Positives	Gaps	Frame
382 bits(980)	8e-129	Compositional matrix adjust.	203/218(93%)	212/218(97%)	0/218(0%)	+3

```
Query  282  KVDPELIFTKQERIGKGSFGEVFKGMDNRTQQVVaikiidleeaedeiediqqeiMVLSQ  461
            KVDPELIFTKQERIGKGSFGEVFKG+DNRTQQVVAIKIIDLEEAEDEI+DIQQEIMVLSQ
Sbjct  6    KVDPELIFTKQERIGKGSFGEVFKGIDNRTQQVVAIKIIDLEEAEDEIDDIQQEIMVLSQ  65

Query  462  CDSPWVTKYYGSYLKGTKLWIIMEYLGGGSALDLMKAGRFEELHIAVILREVLKGLDYLH  641
            CDSP+VTKYYGS+LKGTKLWIIMEYLGGGSALDLMKAG FEE+HI +ILREVLKGLDYLH
Sbjct  66   CDSPYVTKYYGSFLKGTKLWIIMEYLGGGSALDLMKAGSFEEMHIGIILREVLKGLDYLH  125

Query  642  CERKLHRDIKAANVLLSEMGDVKLADFGVAGQLTNTTSKRNTFVGTPFWMAPEVIKQSAY  821
             ERKLHRDIKAANVLLSE GDVKLADFGVAGQLTNTTSKRNTFVGTPFWMAPEVIKQS Y
Sbjct  126  SERKLHRDIKAANVLLSEQGDVKLADFGVAGQLTNTTSKRNTFVGTPFWMAPEVIKQSQY  185

Query  822  DSKADIWSLGITAIELAKGEPPNSDLHPMRVLFLIPRT  935
            D+KADIWSLGITAIELAKGEPPNS+LHPMRVLFLIP+
Sbjct  186  DAKADIWSLGITAIELAKGEPPNSELHPMRVLFLIPKN  223
```

germinal center kinase 1 isoform X4 [Aedes aegypti]

Sequence ID: XP_021700925.1 Length: 691 Number of Matches: 1

Range 1: 43 to 292 GenPept Graphics ▼ Next Match ▲ Previous Match

Score	Expect	Method	Identities	Positives	Gaps	Frame
391 bits(1005)	5e-132	Compositional matrix adjust.	212/250(85%)	228/250(91%)	0/250(0%)	+3

```
Query  186  DSPWQLMRRDMDMSVGDHDDLAVYTIAWALKMKVDPELIFTKQERIGKGSFGEVFKGMDN  365
            DS   +  R  D++  ++D     + + +  MKVDPELIFTKQERIGKGSFGEVFKG+DN
Sbjct  43   DSSVVVASRRQDINSNNNDGPPQMSSSGSEMMKVDPELIFTKQERIGKGSFGEVFKGVDN  102

Query  366  RTQQVVaikiidleeaedeiediqqeiMVLSQCDSPWVTKYYGSYLKGTKLWIIMEYLGG  545
            RTQQVVAIKIIDLEEAEDEIEDIQQEIMVLSQCDSP+VTKY+GSYLKGTKLWIIMEYLGG
Sbjct  103  RTQQVVAIKIIDLEEAEDEIEDIQQEIMVLSQCDSPFVTKYFGSYLKGTKLWIIMEYLGG  162

Query  546  GSALDLMKAGRFEELHIAVILREVLKGLDYLHCERKLHRDIKAANVLLSEMGDVKLADFG  725
            GSALDLMKAG FEE+HIA+ILREVLKGLDYLH ERKLHRDIKAANVLLSE+GDVKLADFG
Sbjct  163  GSALDLMKAGLFEEMHIAIILREVLKGLDYLHSERKLHRDIKAANVLLSELGDVKLADFG  222

Query  726  VAGQLTNTTSKRNTFVGTPFWMAPEVIKQSAYDSKADIWSLGITAIELAKGEPPNSDLHP  905
            VAGQLTNTTSKRNTFVGTPFWMAPEVIKQS YDSKADIWSLGITAIELAKGEPPNS+LHP
Sbjct  223  VAGQLTNTTSKRNTFVGTPFWMAPEVIKQSMYDSKADIWSLGITAIELAKGEPPNSELHP  282

Query  906  MRVLFLIPRT  935
            MRVLFLIP+
Sbjct  283  MRVLFLIPKN  292
```

serine/threonine-protein kinase 26 isoform 1 [Homo sapiens]

Sequence ID: NP_057626.2 Length: 416 Number of Matches: 1

See 5 more title(s) See all Identical Proteins(IPG)

Range 1: 18 to 234 GenPept Graphics ▼ Next Match ▲ Previous Match

Score	Expect	Method	Identities	Positives	Gaps	Frame
347 bits(889)	7e-119	Compositional matrix adjust.	187/217(86%)	203/217(93%)	0/217(0%)	+3

```
Query  285  VDPELIFTKQERIGKGSFGEVFKGMDNRTQQVVaikiidleeaedeiediqqeiMVLSQC  464
             DPE +FTK ERIGKGSFGEVFKG+DNRTQQVVAIKIIDLEEAEDEIEDIQQEI VLSQC
Sbjct  18   ADPEELFTKLERIGKGSFGEVFKGIDNRTQQVVAIKIIDLEEAEDEIEDIQQEITVLSQC  77

Query  465  DSPWVTKYYGSYLKGTKLWIIMEYLGGGSALDLMKAGRFEELHIAVILREVLKGLDYLHC  644
            DS +VTKYYGSYLKG+KLWIIMEYLGGGSALDL++AG F+E  IA +L+E+LKGLDYLH
Sbjct  78   DSSYVTKYYGSYLKGSKLWIIMEYLGGGSALDLLRAGPFDEFQIATMLKEILKGLDYLHS  137

Query  645  ERKLHRDIKAANVLLSEMGDVKLADFGVAGQLTNTTSKRNTFVGTPFWMAPEVIKQSAYD  824
            E+K+HRDIKAANVLLSE GDVKLADFGVAGQLT+T  KRNTFVGTPFWMAPEVI+QSAYD
Sbjct  138  EKKIHRDIKAANVLLSEQGDVKLADFGVAGQLTDTQIKRNTFVGTPFWMAPEVIQQSAYD  197

Query  825  SKADIWSLGITAIELAKGEPPNSDLHPMRVLFLIPRT  935
            SKADIWSLGITAIELAKGEPPNSD+HPMRVLFLIP+
Sbjct  198  SKADIWSLGITAIELAKGEPPNSDMHPMRVLFLIPKN  234
```

Protein alignment of Ir11

TNF receptor-associated factor 5-like [Ixodes scapularis]

Sequence ID: XP_029845770.1 **Length:** 163 **Number of Matches:** 1

Range 1: 1 to 163 GenPept Graphics ▼ Next Match ▲ Previous Match

Score	Expect	Method	Identities	Positives	Gaps	Frame
251 bits(642)	8e-85	Compositional matrix adjust.	124/163(76%)	135/163(82%)	0/163(0%)	+3

```
Query  237  MERKVNMIKGFYPFLDKRQLEFLTSVDEIMSCELCNTITKSYYVAKCRHFFCPDCYSLIQ  416
            MERKV MIK FYP L+ RQLEFLTSVD IMSCELCNTITK YY+A C+HFFC  CYSLI+
Sbjct  1    MERKVKMIKDFYPHLNNRQLEFLTSVDGIMSCELCNTITKYYYMATCKHFFCCTCYSLIE  60

Query  417  KETSPKCPLDNIDWELKTSCCLAECSLNYSRVRCPNTGYGCEYVDYLDRMNDHVGYCAFY  596
            KE  PKCPLD+ D +LKTSCCL E SL+ SRVRCPNTGYGC+    L  MNDHVGYC FY
Sbjct  61   KEEPPKCPLDDSDLKLKTSCCLPEHSLSRSRVRCPNTGYGCKDEGNLTSMNDHVGYCQFY  120

Query  597  PLPCIKCGITVGYNNLVSHLLRSCKFRGNETADPKPAVLDAVE  725
            P+PCIKCG TVG+ NLVSH+  SCK RGNETADPKPAVLDAVE
Sbjct  121  PIPCIKCGDTVGHKNLVSHIRCSCKVRGNETADPKPAVLDAVE  163
```

TRAF6 [Drosophila melanogaster]

Sequence ID: AAD47895.1 **Length:** 475 **Number of Matches:** 1

Range 1: 98 to 227 GenPept Graphics ▼ Next Match ▲ Previous Match

Score	Expect	Method	Identities	Positives	Gaps	Frame
32.7 bits(73)	0.62	Compositional matrix adjust.	35/138(25%)	50/138(36%)	31/138(22%)	+3

```
Query  312  VDEIMSCELCNTITKSYYVAKCRHFFCPDCYSLIQKETSPKCPLDNIDWELKTSCCLAEC  491
            +D    C +C        +  C H FC  C +   ++ +  CP+DN           AE
Sbjct  98   LDSRYECAICIDWLNEPVLTSCGHRFCRSCLTAWMQKNNQCCPMDNKRLS-------AEH  150

Query  492  SL---NYSRVR-------CPNTGYGCEYVDYLDRMNDHVGYCAFY----------PLPCI  611
             +   NY+R         CPN+  GC  V     ++ H+  C +           P   I
Sbjct  151  DIFPDNYTRREIEQLKRDCPNSSLGCSVVASPIELHRHLPSCPYRRQQEPQEEKCPFAKI  210

Query  612  KCGITVGY---NNLVSHL  656
            KC   VG    N L  HL
Sbjct  211  KCDF-VGRPETNQLEEHL  227
```

E3 ubiquitin-protein ligase Bre1 isoform X2 [Aedes aegypti]

Sequence ID: XP_001650655.1 **Length:** 982 **Number of Matches:** 1

See 1 more title(s) See all Identical Proteins(IPG)

Range 1: 911 to 964 GenPept Graphics ▼ Next Match ▲ Previous Match

Score	Expect	Method	Identities	Positives	Gaps	Frame
38.5 bits(88)	0.007	Composition-based stats.	17/54(31%)	25/54(46%)	0/54(0%)	+3

```
Query  279  LDKRQLEFLTSVDEIMSCELCNTITKSYYVAKCRHFFCPDCYSLIQKETSPKCP  440
            +D+  LE +    E ++C  C    K   ++KC H FC DC     +    KCP
Sbjct  911  IDEVMLEEIREYKETLTCPSCKVKRKDAVLSKCFHVFCYDCLRTRYETRQRKCP  964
```

TNF receptor-associated factor 5 isoform b [Homo sapiens]

Sequence ID: NP_001029082.1 **Length:** 557 **Number of Matches:** 1

See 7 more title(s) See all Identical Proteins(IPG)

Range 1: 34 to 164 GenPept Graphics ▼ Next Match ▲ Previous Match

Score	Expect	Method	Identities	Positives	Gaps	Frame
57.4 bits(137)	2e-11	Compositional matrix adjust.	44/134(33%)	60/134(44%)	10/134(7%)	+3

```
Query  297  EFLTSVDEIMSCELCNTITKSYYVAKCRHFFCPDC-YSLIQKETSPKCPLD----NIDWE  461
            +F+  ++E   C  C+++  + +   C H FC  C  SL +  T P CP+D
Sbjct  34   QFVERLEERYKCAFCHSVLHNPHQTGCGHRFCQHCILSLRELNTVPICPVDKEVIKSQEV  93

Query  462  LKTSCCLAECSLNYSRVRCPNTGYGCEYVDYLDRMNDHVGYCAFYPLPCI--KCGITVGY  635
             K +CC  E    Y  V C N   GC     L R  DH+  C F P+ C   KC   V
Sbjct  94   FKDNCCKREVLNLY--VYCSNAP-GCNAKVILGRYQDHLQQCLFQPVQCSNEKCREPVLR  150

Query  636  NNLVSHLLRSCKFR  677
             +L  HL  SC+FR
Sbjct  151  KDLKEHLSASCQFR  164
```

Protein alignment of Ir12

transcription initiation factor TFIID subunit 1 isoform X2 [Ixodes scapularis]

Sequence ID: XP_029839716.1 Length: 1872 Number of Matches: 1

Range 1: 799 to 1058 GenPept Graphics ▼ Next Match ▲ Previous Match

Score	Expect	Method	Identities	Positives	Gaps	Frame
543 bits(1398)	8e-180	Compositional matrix adjust.	260/260(100%)	260/260(100%)	0/260(0%)	+3

```
Query  135   QELPLFEVPGPNSKRANNFVRDFLQVFIYRLFWKSTDSPRRIKMEDIKKAFPSHSESSIR  314
             QELPLFEVPGPNSKRANNFVRDFLQVFIYRLFWKSTDSPRRIKMEDIKKAFPSHSESSIR
Sbjct  799   QELPLFEVPGPNSKRANNFVRDFLQVFIYRLFWKSTDSPRRIKMEDIKKAFPSHSESSIR  858

Query  315   KRLKLCADFKRTGMDSNWWVLKPEFRLPTEDEIRTMVSPEQCCAYYSMISAEQRLKDAGY  494
             KRLKLCADFKRTGMDSNWWVLKPEFRLPTEDEIRTMVSPEQCCAYYSMISAEQRLKDAGY
Sbjct  859   KRLKLCADFKRTGMDSNWWVLKPEFRLPTEDEIRTMVSPEQCCAYYSMISAEQRLKDAGY  918

Query  495   GEKSLFAPEDENDEEMQVKMDDEVKAAPWNTTRAFISSVKGKCLLQLTGVADPTSCGEGF  674
             GEKSLFAPEDENDEEMQVKMDDEVKAAPWNTTRAFISSVKGKCLLQLTGVADPTSCGEGF
Sbjct  919   GEKSLFAPEDENDEEMQVKMDDEVKAAPWNTTRAFISSVKGKCLLQLTGVADPTSCGEGF  978

Query  675   SYVRVPNKPQQSKEDGGSQQPVKKTVTGTDADLRRLSLSNAKQLLRKFGVPEDEIKKLSR  854
             SYVRVPNKPQQSKEDGGSQQPVKKTVTGTDADLRRLSLSNAKQLLRKFGVPEDEIKKLSR
Sbjct  979   SYVRVPNKPQQSKEDGGSQQPVKKTVTGTDADLRRLSLSNAKQLLRKFGVPEDEIKKLSR  1038

Query  855   WEVIDVVRTLSTEQAKAGEE  914
             WEVIDVVRTLSTEQAKAGEE
Sbjct  1039  WEVIDVVRTLSTEQAKAGEE  1058
```

TBP-associated factor 1, isoform F [Drosophila melanogaster]

Sequence ID: NP_001287201.1 Length: 2097 Number of Matches: 1

See 1 more title(s) See all Identical Proteins(IPG)

Range 1: 829 to 1086 GenPept Graphics ▼ Next Match ▲ Previous Match

Score	Expect	Method	Identities	Positives	Gaps	Frame
449 bits(1156)	1e-144	Compositional matrix adjust.	209/260(80%)	239/260(91%)	2/260(0%)	+3

```
Query  135   QELPLFEVPGPNSKRANNFVRDFLQVFIYRLFWKSTDSPRRIKMEDIKKAFPSHSESSIR  314
             QE PL+EVPGPNSKRANNF RDFLQVFIYRLFWKS D+PRRI+M+DIK+AFP+HSESSIR
Sbjct  829   QECPLYEVPGPNSKRANNFTRDFLQVFIYRLFWKSRDNPRRIRMDDIKQAFPAHSESSIR  888

Query  315   KRLKLCADFKRTGMDSNWWVLKPEFRLPTEDEIRTMVSPEQCCAYYSMISAEQRLKDAGY  494
             KRLK CADFKRTGMDSNWWV+KPEFRLP+E+EIR MVSPEQCCAY+SMI+AEQRLKDAGY
Sbjct  889   KRLKQCADFKRTGMDSNWWVIKPEFRLPSEEEIRAMVSPEQCCAYFSMIAAEQRLKDAGY  948

Query  495   GEKSLFAPEDENDEEMQVKMDDEVKAAPWNTTRAFISSVKGKCLLQLTGVADPTSCGEGF  674
             GEK LFAP++++DEE Q+K+DDEVK APWNTTRA+I +++GKCLLQL+G ADPT CGEGF
Sbjct  949   GEKFLFAPQEDDDEEAQLKLDDEVKVAPWNTTRAYIQAMRGKCLLQLSGPADPTGCGEGF  1008

Query  675   SYVRVPNKPQQSKEDGGSQQPVKKTVTGTDADLRRLSLSNAKQLLRKFGVPEDEIKKLSR  854
             SYVRVPNKP Q+KE+  SQ   K++VTGTDADLRRL L  AK+LLR+F VPE+EIKKLSR
Sbjct  1009  SYVRVPNKPTQTKEEQESQP--KRSVTGTDADLRRLPLQRAKELLRQFKVPEEEIKKLSR  1066

Query  855   WEVIDVVRTLSTEQAKAGEE  914
             WEVIDVVRTLSTE+AKAGEE
Sbjct  1067  WEVIDVVRTLSTEKAKAGEE  1086
```

transcription initiation factor TFIID subunit 1 isoform X2 [Aedes aegypti]

Sequence ID: XP_021710567.1 Length: 1962 Number of Matches: 1

Range 1: 815 to 1073 GenPept Graphics ▼ Next Match ▲ Previous Match

Score	Expect	Method	Identities	Positives	Gaps	Frame
449 bits(1155)	7e-145	Compositional matrix adjust.	209/260(80%)	239/260(91%)	1/260(0%)	+3

```
Query  135   QELPLFEVPGPNSKRANNFVRDFLQVFIYRLFWKSTDSPRRIKMEDIKKAFPSHSESSIR  314
             QE PL+EVPGPNSKRANNFVRDFLQVFIYRLFWKS D+PR+I+M+DIKKAFP+HSESSIR
Sbjct  815   QECPLYEVPGPNSKRANNFVRDFLQVFIYRLFWKSKDNPRKIRMDDIKKAFPAHSESSIR  874

Query  315   KRLKLCADFKRTGMDSNWWVLKPEFRLPTEDEIRTMVSPEQCCAYYSMISAEQRLKDAGY  494
             KRLK CADFKRTGMDSN+WV+KPEFRLP+E+EIR MVSPEQCCAY+SMI+AEQRLKDAGY
Sbjct  875   KRLKQCADFKRTGMDSNFWVIKPEFRLPSEEEIRAMVSPEQCCAYFSMIAAEQRLKDAGY  934

Query  495   GEKSLFAPEDENDEEMQVKMDDEVKAAPWNTTRAFISSVKGKCLLQLTGVADPTSCGEGF  674
             GEK +FA ++++DEEMQ+KMDDEVK APWNTTRA+I +++GKC+LQL G ADPT CGEGF
Sbjct  935   GEKFIFAQQEDDDEEMQLKMDDEVKVAPWNTTRAYIQAMRGKCILQLNGPADPTGCGEGF  994

Query  675   SYVRVPNKPQQSKEDGGSQQPVKKTVTGTDADLRRLSLSNAKQLLRKFGVPEDEIKKLSR  854
             SYVR+PNKP Q  ++    QP K+TVTGTDADLRRLSL+NAK LLRKF VPE+EIKKLSR
Sbjct  995   SYVRMPNKPTQQNKEEIESQP-KRTVTGTDADLRRLSLNNAKALLRKFQVPEEEIKKLSR  1053

Query  855   WEVIDVVRTLSTEQAKAGEE  914
             WEVIDVVRTLSTE+AKAGEE
Sbjct  1054  WEVIDVVRTLSTEKAKAGEE  1073
```

transcription initiation factor TFIID subunit 1 isoform X4 [Homo sapiens]

Sequence ID: XP_024308197.1 Length: 1800 Number of Matches: 1

Range 1: 678 to 935 GenPept Graphics ▼ Next Match ▲ Previous Match

Score	Expect	Method	Identities	Positives	Gaps	Frame
466 bits(1199)	8e-151	Compositional matrix adjust.	217/259(84%)	241/259(93%)	1/259(0%)	+3

```
Query  135   QELPLFEVPGPNSKRANNFVRDFLQVFIYRLFWKSTDSPRRIKMEDIKKAFPSHSESSIR  314
             Q+ PLFEVPGPNSKRAN  +RDFLQVFIYRLFWKS D PRRI+MEDIKKAFPSHSESSIR
Sbjct  678   QQCPLFEVPGPNSKRANTHIRDFLQVFIYRLFWKSKDRPRRIRMEDIKKAFPSHSESSIR  737

Query  315   KRLKLCADFKRTGMDSNWWVLKPEFRLPTEDEIRTMVSPEQCCAYYSMISAEQRLKDAGY  494
             KRLKLCADFKRTGMDSNWWVLK +FRLPTE+EIR MVSPEQCCAYYSMI+AEQRLKDAGY
Sbjct  738   KRLKLCADFKRTGMDSNWWVLKSDFRLPTEEEIRAMVSPEQCCAYYSMIAAEQRLKDAGY  797

Query  495   GEKSLFAPEDENDEEMQVKMDDEVKAAPWNTTRAFISSVKGKCLLQLTGVADPTSCGEGF  674
             GEKS FAPE+EN+E+ Q+K+DDEV+ APWNTTRAFI+++KGKCLL++TGVADPT CGEGF
Sbjct  798   GEKSFFAPEEENEEDFQMKIDDEVRTAPWNTTRAFIAAMKGKCLLEVTGVADPTGCGEGF  857

Query  675   SYVRVPNKPQQSKEDGGSQQPVKKTVTGTDADLRRLSLSNAKQLLRKFGVPEDEIKKLSR  854
             SYV++PNKP Q K+D    QPVKKTVTGTDADLRRLSL NAKQLLRKFGVPE+EIKKLSR
Sbjct  858   SYVKIPNKPTQQKDD-KEPQPVKKTVTGTDADLRRLSLKNAKQLLRKFGVPEEEIKKLSR  916

Query  855   WEVIDVVRTLSTEQAKAGE  911
             WEVIDVVRT+STEQA++GE
Sbjct  917   WEVIDVVRTMSTEQARSGE  935
```

Protein alignment of Ir13

RUN domain-containing protein 1 isoform X1 [Ixodes scapularis]

Sequence ID: XP_029837219.1 Length: 564 Number of Matches: 1

See 2 more title(s) See all Identical Proteins(IPG)

Range 1: 1 to 268 GenPept Graphics ▼ Next Match ▲ Previous Match

Score	Expect	Method	Identities	Positives	Gaps	Frame
349 bits(895)	9e-117	Compositional matrix adjust.	266/268(99%)	266/268(99%)	0/268(0%)	+1

```
Query  196   MEVSSPEDIERESPDEDAPLERWAPVGSSEIDDAWDDHDACslseferlrhleeeqeqlN  375
             MEVSS EDIERESPDEDAPLERWAPVGSSEIDDAWDDHDACSLSEFERLRHLEEEQEQLN
Sbjct  1     MEVSSLEDIERESPDEDAPLERWAPVGSSEIDDAWDDHDACSLSEFERLRHLEEEQEQLN  60

Query  376   SSLVALTTHFAQVQFRLKQIVNASQeekevllkeleeFAFRGIPDLRSYQTGVGIllset  555
             SSLVALTTHFAQVQFRLKQIVNASQEEKEVLLKELEEFAFRGIPDLRSYQTGVGILLSET
Sbjct  61    SSLVALTTHFAQVQFRLKQIVNASQEEKEVLLKELEEFAFRGIPDLRSYQTGVGILLSET  120

Query  556   eqeqeekleqqrqkqkelieqlkeqledlekYAFETGEAGMPSSMVLERQTVIIEHlkek  735
             EQEQEEKLEQQRQKQKELIEQLKEQLEDLEKYAFETGEAGMPSSMVLERQTVIIEHLKEK
Sbjct  121   EQEQEEKLEQQRQKQKELIEQLKEQLEDLEKYAFETGEAGMPSSMVLERQTVIIEHLKEK  180

Query  736   lplnldeldlldpddlRKQIDQAVREMVNPVKMKEQLVSQLKTQISDLERFIQFIQGEGS  915
             LPLNLDELDLLDPDDLRKQIDQAVREMVNPVKMKEQLVSQLKTQISDLERFIQFIQGEGS
Sbjct  181   LPLNLDELDLLDPDDLRKQIDQAVREMVNPVKMKEQLVSQLKTQISDLERFIQFIQGEGS  240

Query  916   DGKPRCTCNCPVHGKVDVSHSETSSECY  999
             DGKPRCTCNCPVHGKVDV HSETSSECY
Sbjct  241   DGKPRCTCNCPVHGKVDVLHSETSSECY  268
```

uncharacterized protein Dmel_CG3703 [Drosophila melanogaster]

Sequence ID: NP_569874.1 Length: 711 Number of Matches: 1

See 2 more title(s) See all Identical Proteins(IPG)

Range 1: 112 to 281 GenPept Graphics ▼ Next Match ▲ Previous Match

Score	Expect	Method	Identities	Positives	Gaps	Frame
81.6 bits(200)	1e-16	Compositional matrix adjust.	79/177(45%)	116/177(65%)	10/177(5%)	+1

```
Query  400   HFAQVQFRLKQIVNASQeekevllkeleeFAFRGIPDLRSYQTGVGIllseteqeqeekl  579
             HFA VQ R++QIV A  EE++ LL++LE+FAF+GIPD  + Q+           +
Sbjct  112   HFAHVQLRVRQIVEAPAEERDQLLRDLEDFAFQGIPD--AVQS-----KESHPDKPASDG  164

Query  580   eqqrqkqkelieqlkeqledlekYAFETGEAG-MPSSMVLERQTVIIEHlkeklplnlde  756
             E+      +LI+QLK QL +LE+ A+E GE G +P  ++LE+Q  I++ L+ KL L +++
Sbjct  165   EKDHGPDSQLIQQLKSQLTELEQIAYEAGEPGILPQHVLLEKQKFILDELRAKLNLQVEQ  224

Query  757   ld--lldpddlRKQIDQAVREMVNPVKMKEQLVSQLKTQISDLERFIQFIQGEGSDG  921
              +   L  + LR Q+D A+ E V P+KMKEQLV+QLKTQI+DLERFI F+Q +  +G
Sbjct  225   HELPALSTEQLRHQVDNAIGEFVGPLKMKEQLVAQLKTQITDLERFIAFLQCDAIEG  281
```

RUN domain-containing protein 1 [Aedes aegypti]

Sequence ID: XP_001657703.1 Length: 700 Number of Matches: 1

See 1 more title(s) See all Identical Proteins(IPG)

Range 1: 102 to 281 GenPept Graphics ▼ Next Match ▲ Previous Match

Score	Expect	Method	Identities	Positives	Gaps	Frame
101 bits(252)	2e-23	Compositional matrix adjust.	100/180(56%)	132/180(73%)	4/180(2%)	+1

```
Query  376   SSLVALTTHFAQVQFRLKQIVNASQeekevllkeleeFAFRGIPDLRSYQTGVGIllset  555
             S+L+ALT+HFAQVQ RL+QIV A  EE++ LLK LEEFAF GIP+L+        I
Sbjct  102   SNLIALTSHFAQVQLRLRQIVEAPPEERDNLLKNLEEFAFLGIPELQQDSNKKNIPEIVA  161

Query  556   eqeqe-ekleqqrqkqkelieqlkeqledlekYAFETGEAGMPSSMVLERQTVIIEHlke  732
             + ++  E +E  R+KQ  LI+QLK QL DLE+YA+E+G   +P +++LE+Q VII+ +K
Sbjct  162   DLDRSPESVEHLREKQHALIDQLKNQLIDLERYAYESGAGILPHTILLEKQKVIIDEIKN  221

Query  733   klplnldeld--lldpddlRKQIDQAVRE-MVNPVKMKEQLVSQLKTQISDLERFIQFIQ  903
             K+ LNL+ELD   L  +DLR Q+D A+ E +VNP+KMKEQLVSQLKTQI DLERFI F+Q
Sbjct  222   KINLNLNELDLPQLTTEDLRNQVDTALDEQLVNPLKMKEQLVSQLKTQIQDLERFISFLQ  281
```

RecName: Full=RUN domain-containing protein 1 [Homo sapiens]

Sequence ID: Q96C34.3 Length: 613 Number of Matches: 1

See 1 more title(s) See all Identical Proteins(IPG)

Range 1: 91 to 316 GenPept Graphics ▼ Next Match ▲ Previous Match

Score	Expect	Method	Identities	Positives	Gaps	Frame
95.1 bits(235)	3e-25	Compositional matrix adjust.	105/228(46%)	146/228(64%)	24/228(10%)	+1

```
Query  373   NSSLVALTTHFAQVQFRLKQIVNASQeekevllkeleeFAFRGIPDLRSYQ--------T  528
             +S+L+AL++HFAQVQFRL+Q+V  +  E++ LL+ELE+FAFRG P +  Y+
Sbjct  91    DSALLALSSHFAQVQFRLRQVVRGAPAEQQRLLRELEDFAFRGCPHVLGYEGPGDPASDE  150

Query  529   GVGI--------llseteqeqeekleqqrqkqkelieqlkeqledlekYAFETGEA-GMP  681
             G G+           ++EQE++E+LE QR+KQKELI QLK QL+DLE +A++ G    +P
Sbjct  151   GDGLPGDRPWLRGEDQSEQEKQERLETQREKQKELILQLKTQLDDLETFAYQEGSYDSLP  210

Query  682   SSMVLERQTVII-EHlkeklplnldeldlldpddlRKQIDQAVREMVNPVKMKEQLVSQL  858
              S+VLERQ VII E +K+      +++  L  ++LR+++D AV ++VNP ++KEQLV QL
Sbjct  211   QSVVLERQRVIIDELIKKLDMNLNEDISSLSTEELRQRVDAAVAQIVNPARVKEQLVEQL  270

Query  859   KTQISDLERFIQFIQGEGSDGKPRCT----CNCPVHGKVDVSHSETSS  990
             KTQI DLE FI FIQ E   G P  T    C C   GK     S T S
Sbjct  271   KTQIRDLEMFINFIQDE--VGSPLQTGGGHCECKAGGKTGNGCSRTGS  316
```

Protein alignment of Ir14

methionine--tRNA ligase, cytoplasmic [Ixodes scapularis]

Sequence ID: XP_029846836.1 **Length:** 962 **Number of Matches:** 1

Range 1: 251 to 492 GenPept Graphics ▼ Next Match ▲ Previous Match

Score	Expect	Method	Identities	Positives	Gaps	Frame
429 bits(1103)	1e-143	Compositional matrix adjust.	240/242(99%)	240/242(99%)	0/242(0%)	+2

```
Query  215  LTSPMKVKAKVEANGeaspeikleeekelsaaeVGKAFSAWNDPKNLAkelkkkpcpilp  394
            LTSPMKVKAKVEANGEASPEIKLEEEKELSAAEVGKAF AWNDPKNLAKELKKKPCPILP
Sbjct  251  LTSPMKVKAKVEANGEASPEIKLEEEKELSAAEVGKAFLAWNDPKNLAKELKKKPCPILP  310

Query  395  kkGERNVLITSALPYVNNVPHLGNIVGSVLSADVFARYCRLRNWNTLYVCGTDEYGTATE  574
            KKGERNVLITSALPYVNNVPHLGNIVGSVLSADVFARYCRLRNWNTLYVCGTDEYGTATE
Sbjct  311  KKGERNVLITSALPYVNNVPHLGNIVGSVLSADVFARYCRLRNWNTLYVCGTDEYGTATE  370

Query  575  TKALEMGITPREICDRFNKLHADIYKWFNISFDHFGRTTTEQQTKIAQDIFLKLHKNQYM  754
            TKALEMGITPREICDRFNKLHADIYKWFNISFDHFGRTTTEQQTKIAQDIFLKLHKNQYM
Sbjct  371  TKALEMGITPREICDRFNKLHADIYKWFNISFDHFGRTTTEQQTKIAQDIFLKLHKNQYM  430

Query  755  LEEVVEQLFCNRCERFLADRFVEGTCPFCAYEDARGDQCDLCSKLINPTELKQARCKLCK  934
             EEVVEQLFCNRCERFLADRFVEGTCPFCAYEDARGDQCDLCSKLINPTELKQARCKLCK
Sbjct  431  REEVVEQLFCNRCERFLADRFVEGTCPFCAYEDARGDQCDLCSKLINPTELKQARCKLCK  490

Query  935  EP  940
            EP
Sbjct  491  EP  492
```

Methionyl-tRNA synthetase [Drosophila melanogaster]

Sequence ID: NP_611382.1 **Length:** 1022 **Number of Matches:** 1

See 1 more title(s) See all Identical Proteins(IPG)

Range 1: 252 to 430 GenPept Graphics ▼ Next Match ▲ Previous Match

Score	Expect	Method	Identities	Positives	Gaps	Frame
266 bits(680)	2e-81	Compositional matrix adjust.	121/179(68%)	143/179(79%)	2/179(1%)	+2

```
Query  401  GERNVLITSALPYVNNVPHLGNIVGSVLSADVFARYCRLRNWNTLYVCGTDEYGTATETK  580
            GERNVLITSALPYVNNVPHLGNI+G VLSAD++ARY R   +NTL +CGTDEYGTATE K
Sbjct  252  GERNVLITSALPYVNNVPHLGNIIGCVLSADIYARYSRSAGYNTLLICGTDEYGTATENK  311

Query  581  ALEMGITPREICDRFNKLHADIYKWFNISFDHFGRTTTEQQTKIAQDIFLKLHKNQYMLE  760
            AL   +TPREICD++ +LH  IY+WF I FD+FGRTTT++QT I Q+ F  + K  Y++
Sbjct  312  ALAENLTPREICDKYFELHNAIYRWFGIGFDYFGRTTTQEQTDIVQEAFKDVLKAGYIIT  371

Query  761  EVVEQLFCNRCERFLADRFVEGTCPF--CAYEDARGDQCDLCSKLINPTELKQARCKLC  931
            E VEQL C +C+RFLADRFVEGTCP   C YEDARGDQCD C KL+N TEL + RCK+C
Sbjct  372  ESVEQLLCQKCDRFLADRFVEGTCPHPGCGYEDARGDQCDKCGKLVNATELIRPRCKVC  430
```

methionine--tRNA ligase, cytoplasmic [Aedes aegypti]

Sequence ID: XP_001649418.2 **Length:** 966 **Number of Matches:** 1

Range 1: 254 to 432 GenPept Graphics ▼ Next Match ▲ Previous Match

Score	Expect	Method	Identities	Positives	Gaps	Frame
262 bits(670)	3e-80	Compositional matrix adjust.	117/179(65%)	144/179(80%)	2/179(1%)	+2

```
Query  401  GERNVLITSALPYVNNVPHLGNIVGSVLSADVFARYCRLRNWNTLYVCGTDEYGTATETK  580
            G++N LITSALPYVNNVPHLGNI+G VLSADVFARY RL  +NTLY+CGTDEYGTATETK
Sbjct  254  GQKNNLITSALPYVNNVPHLGNIIGCVLSADVFARYSRLCGYNTLYICGTDEYGTATETK  313

Query  581  ALEMGITPREICDRFNKLHADIYKWFNISFDHFGRTTTEQQTKIAQDIFLKLHKNQYMLE  760
            AL   +TP++ICD++ ++H  IY+WF I FD+FGRTTT +QT+I QD+F  L+    ++
Sbjct  314  ALAENLTPKQICDKYFEIHNSIYRWFGIGFDYFGRTTTAEQTQIVQDMFKDLYAGGFIET  373

Query  761  EVVEQLFCNRCERFLADRFVEGTCPF--CAYEDARGDQCDLCSKLINPTELKQARCKLC  931
              V+QL C +C+R+LADRFVEGTCP+  C YEDARGDQCD C KLIN  EL + RCK+C
Sbjct  374  RAVDQLLCQKCDRYLADRFVEGTCPYPGCGYEDARGDQCDACGKLINAIELVRPRCKVC  432
```

methionine--tRNA ligase, cytoplasmic isoform X1 [Homo sapiens]

Sequence ID: XP_006719461.1 **Length:** 666 **Number of Matches:** 1

Range 1: 28 to 205 GenPept Graphics ▼ Next Match ▲ Previous Match

Score	Expect	Method	Identities	Positives	Gaps	Frame
297 bits(760)	8e-95	Compositional matrix adjust.	135/178(76%)	154/178(86%)	0/178(0%)	+2

```
Query  401  GERNVLITSALPYVNNVPHLGNIVGSVLSADVFARYCRLRNWNTLYVCGTDEYGTATETK  580
            GERNVLITSALPYVNNVPHLGNI+G VLSADVFARY RLR WNTLY+CGTDEYGTATETK
Sbjct  28   GERNVLITSALPYVNNVPHLGNIIGCVLSADVFARYSRLRQWNTLYLCGTDEYGTATETK  87

Query  581  ALEMGITPREICDRFNKLHADIYKWFNISFDHFGRTTTEQQTKIAQDIFLKLHKNQYMLE  760
            ALE G+TP+EICD+++ +HADIY+WFNISFD FGRTTT QQTKI QDIF +L K  ++L+
Sbjct  88   ALEEGLTPQEICDKYHIIHADIYRWFNISFDIFGRTTTPQQTKITQDIFQQLLKRGFVLQ  147

Query  761  EVVEQLFCNRCERFLADRFVEGTCPFCAYEDARGDQCDLCSKLINPTELKQARCKLCK  934
            + VEQL C  C RFLADRFVEG CPFC YE+ARGDQCD C KLIN  ELK+ +CK+C+
Sbjct  148  DTVEQLRCEHCARFLADRFVEGVCPFCGYEEARGDQCDKCGKLINAVELKKPQCKVCR  205
```

Protein alignment of Ir15

unconventional myosin-Va [Ixodes scapularis]

Sequence ID: XP_029843866.1 **Length:** 1825 **Number of Matches:** 2

Range 1: 1203 to 1441 GenPept Graphics ▼ Next Match ▲ Previous Match

Score	Expect	Method	Identities	Positives	Gaps	Frame
447 bits(1150)	3e-144	Compositional matrix adjust.	239/239(100%)	239/239(100%)	0/239(0%)	+3

```
Query  177    KELMRLHELEMENSKLKEDVKKMVKTLATEDKTSREKDLMNHYESIQDelerrreeclql  356
              KELMRLHELEMENSKLKEDVKKMVKTLATEDKTSREKDLMNHYESIQDELERRREECLQL
Sbjct  1203   KELMRLHELEMENSKLKEDVKKMVKTLATEDKTSREKDLMNHYESIQDELERRREECLQL  1262

Query  357    rAVLANQSEDLKSVVALDSYRGNMDRLNEDGELLMAFETQKKLIRQLEAELQTEKVRMQS  536
              RAVLANQSEDLKSVVALDSYRGNMDRLNEDGELLMAFETQKKLIRQLEAELQTEKVRMQS
Sbjct  1263   RAVLANQSEDLKSVVALDSYRGNMDRLNEDGELLMAFETQKKLIRQLEAELQTEKVRMQS  1322

Query  537    MEHEFRDEIKKLQEDNDRQQKLISQNLKKTPQAQSDAILQHEINRLTGENVDLREKIDGL  716
              MEHEFRDEIKKLQEDNDRQQKLISQNLKKTPQAQSDAILQHEINRLTGENVDLREKIDGL
Sbjct  1323   MEHEFRDEIKKLQEDNDRQQKLISQNLKKTPQAQSDAILQHEINRLTGENVDLREKIDGL  1382

Query  717    AEQLKKYKRQLKIYAKKMKDGGILDQAEVKVEVGEDHGKNMPEIKHKDAQNLGMFEYKK  893
              AEQLKKYKRQLKIYAKKMKDGGILDQAEVKVEVGEDHGKNMPEIKHKDAQNLGMFEYKK
Sbjct  1383   AEQLKKYKRQLKIYAKKMKDGGILDQAEVKVEVGEDHGKNMPEIKHKDAQNLGMFEYKK  1441
```

Range 2: 1454 to 1474 GenPept Graphics ▼ Next Match ▲ Previous Match ⩓ First Match

Score	Expect	Method	Identities	Positives	Gaps	Frame
38.5 bits(88)	0.012	Compositional matrix adjust.	18/21(86%)	19/21(90%)	0/21(0%)	+2

```
Query  929    GLEAKLAVTLLPGLPAYILFM  991
               L+ KLAVTLLPGLPAYILFM
Sbjct  1454   DLKPKLAVTLLPGLPAYILFM  1474
```

myosin V [Drosophila melanogaster]

Sequence ID: AAC99496.1 **Length:** 1792 **Number of Matches:** 2

Range 1: 1185 to 1412 GenPept Graphics ▼ Next Match ▲ Previous Match

Score	Expect	Method	Identities	Positives	Gaps	Frame
46.2 bits(108)	1e-08	Compositional matrix adjust.	75/249(30%)	127/249(51%)	38/249(15%)	+3

```
Query  186    MRLHELEMENSKLKEDVKKMVKTLATEDKTSREKDLMN-HYESIQDelerrreeclqlrA  362
               R+ ELE+EN KL+ +  +    L T  K   E + +N  + ++Q+E+ RRREEC+QL+A
Sbjct  1185   FRVSELEVENEKLRSEYDQ----LRTSIKHGVEINELNAQHAALQEEVRRRREECIQLKA  1240

Query  363    VLANQSEDLKSVVALDSYRGNMDRLNEDGELLMAFETQKKLIRQLEAELQTEKVRMQSME  542
              VL  QS+ ++S   L+     M R N+  EL+ AF +QK + RQLE+EL+       S
Sbjct  1241   VLLQQSQSMRS---LEPESLQM-RGNDVNELMEAFHSQKLINRQLESELKAITEEHNSKL  1296

Query  543    HEFRDEIKKLQEDNDRQQKLISQNLKKTPQA------QSDAILQHEINRLTGENVDLREK  704
               E   EI++L  + D  QK++ +++ +   +      Q+D  L+ E+ +   + + ++E+
Sbjct  1297   VEMTQEIERLNNEKDELQKVMFESIDEFEDSNVDTLRQNDRYLRRELQKAVAQFLLVQEE  1356

Query  705    IDGLAEQLKKYKRQLKIYAKKMKDGGILDQAEVKVE----------VGEDHGKNMPEIKH  854
              +     +LK Y+          +DGG   Q E K+E             D G N+ + K
Sbjct  1357   LKLANAKLKAYR----------QDGG---QLEHKIEEEMIRNKSNGTSADVGANVTKQKS  1403

Query  855    KDAQNLGMF  881
              ++ Q L  F
Sbjct  1404   QNPQGLMKF  1412
```

unconventional myosin-Vb isoform X2 [Aedes aegypti]

Sequence ID: XP_021696415.1 **Length:** 1888 **Number of Matches:** 2

Range 1: 1284 to 1488 GenPept Graphics ▼ Next Match ▲ Previous Match

Score	Expect	Method	Identities	Positives	Gaps	Frame
56.6 bits(135)	2e-08	Compositional matrix adjust.	62/209(30%)	117/209(55%)	13/209(6%)	+3

```
Query  186    MRLHELEMENSKLKEDVKKMVKTLATEDKTSREKDLMNHYESIQDelerrreeclqlrAV  365
              +R  ELE+E  KL++D   +  ++    +   +++L   + ++Q+EL+RRR+EC+ L+AV
Sbjct  1284   IRASELEVEIEKLRQDYNLLRNSI---KRGVEDRELEAQHNALQEELKRRRDECISLKAV  1340

Query  366    LANQSEDLKSVVALDSYRGNMD---RLNEDGELLMAFETQKKLIRQLEAELQTEKVRMQS  536
              LA QS  L+++        N D   R++++GEL+ AF+ QK + RQLE+EL+
Sbjct  1341   LAQQSHSLRTL-GQSQVNPNGDSSLRIHDEGELMEAFQAQKLVNRQLESELRAMMDASNE  1399

Query  537    MEHEFRDEIKKLQEDNDRQQKLISQNLKKTPQ------AQSDAILQHEINRLTGENVDLR  698
                 E    I+ L+++    Q ++   L  + +       QSD  L+HE+ + T   VDL+
Sbjct  1400   TLVENNKVIEALRKEVQELQMILQGRLDTSNEDNLEALRQSDQYLRHELKKSTAAYVDLQ  1459

Query  699    EKIDGLAEQLKKYKRQLKIYAKKMKDGGI  785
              E+++ L  ++ +  ++  I + +++D GI
Sbjct  1460   EQVNELLAKINELTKKNNILSNRLRDNGI  1488
```

Range 2: 1527 to 1552 GenPept Graphics ▼ Next Match ▲ Previous Match ⩓ First Match

Score	Expect	Method	Identities	Positives	Gaps	Frame
37.0 bits(84)	0.030	Composition-based stats.	14/26(54%)	22/26(84%)	0/26(0%)	+2

```
Query  914    QKPYSGLEAKLAVTLLPGLPAYILFM  991
              Q+  + L+ ++AVTL+PGLPAY++FM
Sbjct  1527   QRLVTDLKPRVAVTLIPGLPAYVVFM  1552
```

unconventional myosin-Va isoform 5 [Homo sapiens]

Sequence ID: NP_001369278.1 **Length:** 1879 **Number of Matches:** 2

Range 1: 1353 to 1505 GenPept Graphics ▼ Next Match ▲ Previous Match

Score	Expect	Method	Identities	Positives	Gaps	Frame
90.1 bits(222)	4e-21	Compositional matrix adjust.	68/164(41%)	103/164(62%)	22/164(13%)	+3

```
Query  435    LNEDGELLMAFETQKKLIRQLEAELQTEKVRMQSMEHEFRDEIKKLQEDNDRQQKLISQN  614
              LNEDGEL + +E  K+  R LE++LQ++K   ++     R EI+ L+E+N+RQQ+L++QN
Sbjct  1353   LNEDGELWLVYEGLKQANRLLESQLQSQKRSHENEAEALRGEIQSLKEENNRQQQLLAQN  1412

Query  615    LKKTPQAQSDAILQHEINRLTGENVDLREKIDGLAEQLKKYKRQLKIYAKKMKD------  776
              L+  P+A+ +A LQHEI RLT EN+DL E+++   + ++K K+QLK++AKK+ +
Sbjct  1413   LQLPPEARIEASLQHEITRLTNENLDLMEQLEKQDKTVRKLKKQLKVFAKKIGELEVGQM  1472

Query  777    -----GGILDQAEVKVEVGEDHGKNMPEIKHKDAQNLGMFEYKK  893
                   G I+D+    V        N+P  K KD Q  GM EYKK
Sbjct  1473   ENISPGQIIDEPIRPV--------NIPR-KEKDFQ--GMLEYKK  1505
```

Range 2: 1505 to 1539 GenPept Graphics ▼ Next Match ▲ Previous Match ⩓ First Match

Score	Expect	Method	Identities	Positives	Gaps	Frame
33.1 bits(74)	4e-21	Composition-based stats.	19/35(54%)	21/35(60%)	5/35(14%)	+2

```
Query  902    KFDHQKPYSGLEAKL-----AVTLLPGLPAYILFM  991
              K D QK    L  +L     AV L+PGLPAYILFM
Sbjct  1505   KEDEQKLVKNLILELKPRGVAVNLIPGLPAYILFM  1539
```

Protein alignment of Ir16

adenosine kinase 2 [Ixodes scapularis]

Sequence ID: XP_002405419.1 **Length:** 348 **Number of Matches:** 1

See 1 more title(s) See all Identical Proteins(IPG)

Range 1: 153 to 348 GenPept Graphics ▼ Next Match ▲ Previous Match

Score	Expect	Method	Identities	Positives	Gaps	Frame
397 bits(1020)	2e-139	Compositional matrix adjust.	191/196(97%)	194/196(98%)	0/196(0%)	+3

```
Query  120  QPDNKALMEEASHYYISGFFLSVSLDSILTVAKHACSKGKTFCMNLSAPFLCRIFKEQMM  299
            +PDNKALMEEAS+YYISGFFLSVSLDSILTVAKHACSKGKTFCMNLSAPFLC IFKEQMM
Sbjct  153  KPDNKALMEEASYYYISGFFLSVSLDSILTVAKHACSKGKTFCMNLSAPFLCSIFKEQMM  212

Query  300  QAFPYIDILFGNETEAREFADVHNLGTTDTVEIAKLISKFPKESGHFERMVVITQGAEDV  479
            QAFPYIDILFGNETEAREFA+VHNLGTTDTVEIAKLISKFPKESGHFERMVVITQGAEDV
Sbjct  213  QAFPYIDILFGNETEAREFANVHNLGTTDTVEIAKLISKFPKESGHFERMVVITQGAEDV  272

Query  480  IVAQGNDTQTFPVPKLKTEDIVDTNGAGDAFVGGFLAMYLLGKPIETCVRCGITVSVEVI  659
            IVAQGNDTQTF VPKLKTEDIVDTNGAGDAFVGGFLAMYLLGKPIETCVRCGITVSVEVI
Sbjct  273  IVAQGNDTQTFSVPKLKTEDIVDTNGAGDAFVGGFLAMYLLGKPIETCVRCGITVSVEVI  332

Query  660  KKSGCTLPDRESVHVV  707
            KKSGCTLPDRESVHVV
Sbjct  333  KKSGCTLPDRESVHVV  348
```

adenosine kinase, isoform C [Drosophila melanogaster]

Sequence ID: NP_001287054.1 **Length:** 345 **Number of Matches:** 1

See 4 more title(s) See all Identical Proteins(IPG)

Range 1: 151 to 336 GenPept Graphics ▼ Next Match ▲ Previous Match

Score	Expect	Method	Identities	Positives	Gaps	Frame
170 bits(430)	3e-50	Compositional matrix adjust.	86/186(46%)	124/186(66%)	0/186(0%)	+3

```
Query  120  QPDNKALMEEASHYYISGFFLSVSLDSILTVAKHACSKGKTFCMNLSAPFLCRIFKEQMM  299
            +P NKAL++ A +YYISGFFL+V+  SI+ VA  A +K + F MNLSAPF+ + +   ++
Sbjct  151  EPSNKALVDNAQYYYISGFFLTVNPPSIMQVAATAHAKQRPFLMNLSAPFISQFYMAPLL  210

Query  300  QAFPYIDILFGNETEAREFADVHNLGTTDTVEIAKLISKFPKESGHFERMVVITQGAEDV  479
             A PY+DI+FGNE EA+ FA+     + D  EI K +    K++    R+ ++TQG + V
Sbjct  211  AALPYVDIIFGNEAEAQAFAEAQQWPSGDLREIGKRLVAMEKKNPTRPRIAILTQGCDPV  270

Query  480  IVAQGNDTQTFPVPKLKTEDIVDTNGAGDAFVGGFLAMYLLGKPIETCVRCGITVSVEVI  659
            ++ Q +  Q FPV KL   +IVDTNGAGDAFVGGFL+ ++ GK ++ C+RCG   +  +I
Sbjct  271  LLIQQDSVQEFPVTKLAVHEIVDTNGAGDAFVGGFLSQFVQGKSLDVCIRCGNYAAGHII  330

Query  660  KKSGCT  677
            K  GCT
Sbjct  331  KNPGCT  336
```

adenosine kinase [Aedes aegypti]

Sequence ID: XP_001652432.2 **Length:** 344 **Number of Matches:** 1

Range 1: 149 to 344 GenPept Graphics ▼ Next Match ▲ Previous Match

Score	Expect	Method	Identities	Positives	Gaps	Frame
204 bits(518)	2e-63	Compositional matrix adjust.	99/196(51%)	136/196(69%)	3/196(1%)	+3

```
Query  117  RQPDNKALMEEASHYYISGFFLSVSLDSILTVAKHACSKGKTFCMNLSAPFLCRIFKEQM  296
            +  +N+  ++ A ++YISGFFL+VSL+SILTVAKHA SK + F MNLSAPF+ + FK+ +
Sbjct  149  KSAENEKYLQNAEYFYISGFFLTVSLESILTVAKHALSKDRLFMMNLSAPFIPQFFKDNL  208

Query  297  MQAFPYIDILFGNETEAREFADVHNLGTTDTVEIAKLISKFPKESGHFERMVVITQGAED  476
             Q  PYIDILFGNETEA  FA+    GT D  EI   IS  PK++   +R+ +ITQG++
Sbjct  209  DQVMPYIDILFGNETEALAFAEAQKFGTEDLKEIGLKISALPKQNESRKRVAIITQGSDP  268

Query  477  VIVAQGNDTQTFPVPKLKTEDIVDTNGAGDAFVGGFLAMYLLGKPIETCVRCGITVSVEV  656
            V++ +      FPV KL + IVDTNGAGDAFVGGFLA  +  +  +TC+ CGI  + ++
Sbjct  269  VLLIRDGTITEFPVEKLAADQIVDTNGAGDAFVGGFLAQLVQKRNFDTCIECGIWAARKI  328

Query  657  IKKSGCT---LPDRES  695
            I++SGCT   +PD E+
Sbjct  329  IQRSGCTFEGVPDFEA  344
```

adenosine kinase [Homo sapiens]

Sequence ID: AAB01689.1 **Length:** 334 **Number of Matches:** 1

Range 1: 144 to 334 GenPept Graphics ▼ Next Match ▲ Previous Match

Score	Expect	Method	Identities	Positives	Gaps	Frame
181 bits(458)	9e-54	Compositional matrix adjust.	86/191(45%)	121/191(63%)	0/191(0%)	+3

```
Query  129  NKALMEEASHYYISGFFLSVSLDSILTVAKHACSKGKTFCMNLSAPFLCRIFKEQMMQAF  308
            N  L+E+A   YI+GFFL+VS +S+L VA HA    + F +NLSAPF+ + +KE +M+
Sbjct  144  NWMLVEKARVCYIAGFFLTVSPESVLKVAHHASENNRIFTLNLSAPFISQFYKESLMKVM  203

Query  309  PYIDILFGNETEAREFADVHNLGTTDTVEIAKLISKFPKESGHFERMVVITQGAEDVIVA  488
            PY+DILFGNETEA  FA      T D  EIAK     PK +   +R+V+ TQG +D I+A
Sbjct  204  PYVDILFGNETEAATFAREQGFETKDIKEIAKKTQALPKMNSKRQRIVIFTQGRDDTIMA  263

Query  489  QGNDTQTFPVPKLKTEDIVDTNGAGDAFVGGFLAMYLLGKPIETCVRCGITVSVEVIKKS  668
              ++   F V      +I+DTNGAGDAFVGGFL+  +  KP+  C+R G   +  +I+++
Sbjct  264  TESEVTAFAVLDQDQREIIDTNGAGDAFVGGFLSQLVSDKPLTECIRAGHYAASIIIRRT  323

Query  669  GCTLPDRESVH  701
            GCT P++   H
Sbjct  324  GCTFPEKPDFH  334
```

Protein alignment of Ir17

serine/threonine-protein kinase 26 isoform X2 [Ixodes scapularis]

Sequence ID: XP_029827735.1 **Length:** 574 **Number of Matches:** 1

Range 1: 125 to 226 GenPept Graphics ▼ Next Match ▲ Previous Match

Score	Expect	Method	Identities	Positives	Gaps	Frame
218 bits(554)	1e-69	Compositional matrix adjust.	102/102(100%)	102/102(100%)	0/102(0%)	+3

```
Query  3    DYLHCERKLHRDIKAANVLLSEMGDVKLADFGVAGQLTNTTSKRNTFVGTPFWMAPEVIK  182
            DYLHCERKLHRDIKAANVLLSEMGDVKLADFGVAGQLTNTTSKRNTFVGTPFWMAPEVIK
Sbjct  125  DYLHCERKLHRDIKAANVLLSEMGDVKLADFGVAGQLTNTTSKRNTFVGTPFWMAPEVIK  184

Query  183  QSAYDSKADIWSLGITAIELAKGEPPNSDLHPMRVLFLIPKN  308
            QSAYDSKADIWSLGITAIELAKGEPPNSDLHPMRVLFLIPKN
Sbjct  185  QSAYDSKADIWSLGITAIELAKGEPPNSDLHPMRVLFLIPKN  226
```

germinal centre kinase III [Drosophila melanogaster]

Sequence ID: NP_650596.1 **Length:** 642 **Number of Matches:** 1

See 2 more title(s) ⌄ See all Identical Proteins(IPG)

Range 1: 122 to 223 GenPept Graphics ▼ Next Match ▲ Previous Match

Score	Expect	Method	Identities	Positives	Gaps	Frame
207 bits(528)	4e-65	Compositional matrix adjust.	97/102(95%)	99/102(97%)	0/102(0%)	+3

```
Query  3    DYLHCERKLHRDIKAANVLLSEMGDVKLADFGVAGQLTNTTSKRNTFVGTPFWMAPEVIK  182
            DYLH ERKLHRDIKAANVLLSE GDVKLADFGVAGQLTNTTSKRNTFVGTPFWMAPEVIK
Sbjct  122  DYLHSERKLHRDIKAANVLLSEQGDVKLADFGVAGQLTNTTSKRNTFVGTPFWMAPEVIK  181

Query  183  QSAYDSKADIWSLGITAIELAKGEPPNSDLHPMRVLFLIPKN  308
            QS YD+KADIWSLGITAIELAKGEPPNS+LHPMRVLFLIPKN
Sbjct  182  QSQYDAKADIWSLGITAIELAKGEPPNSELHPMRVLFLIPKN  223
```

serine/threonine-protein kinase 26 isoform X5 [Aedes aegypti]

Sequence ID: XP_021700926.1 **Length:** 622 **Number of Matches:** 1

Range 1: 122 to 223 GenPept Graphics ▼ Next Match ▲ Previous Match

Score	Expect	Method	Identities	Positives	Gaps	Frame
211 bits(538)	7e-67	Compositional matrix adjust.	98/102(96%)	100/102(98%)	0/102(0%)	+3

```
Query  3    DYLHCERKLHRDIKAANVLLSEMGDVKLADFGVAGQLTNTTSKRNTFVGTPFWMAPEVIK  182
            DYLH ERKLHRDIKAANVLLSE+GDVKLADFGVAGQLTNTTSKRNTFVGTPFWMAPEVIK
Sbjct  122  DYLHSERKLHRDIKAANVLLSELGDVKLADFGVAGQLTNTTSKRNTFVGTPFWMAPEVIK  181

Query  183  QSAYDSKADIWSLGITAIELAKGEPPNSDLHPMRVLFLIPKN  308
            QS YDSKADIWSLGITAIELAKGEPPNS+LHPMRVLFLIPKN
Sbjct  182  QSMYDSKADIWSLGITAIELAKGEPPNSELHPMRVLFLIPKN  223
```

serine/threonine-protein kinase 25 isoform 3 [Homo sapiens]

Sequence ID: NP_001269234.1 **Length:** 332 **Number of Matches:** 1

See 2 more title(s) ⌄ See all Identical Proteins(IPG)

Range 1: 35 to 136 GenPept Graphics ▼ Next Match ▲ Previous Match

Score	Expect	Method	Identities	Positives	Gaps	Frame
200 bits(508)	2e-64	Compositional matrix adjust.	95/102(93%)	97/102(95%)	0/102(0%)	+3

```
Query  3    DYLHCERKLHRDIKAANVLLSEMGDVKLADFGVAGQLTNTTSKRNTFVGTPFWMAPEVIK  182
            DYLH ERK+HRDIKAANVLLSE GDVKLADFGVAGQLT+T  KRNTFVGTPFWMAPEVIK
Sbjct  35   DYLHSERKIHRDIKAANVLLSEQGDVKLADFGVAGQLTDTQIKRNTFVGTPFWMAPEVIK  94

Query  183  QSAYDSKADIWSLGITAIELAKGEPPNSDLHPMRVLFLIPKN  308
            QSAYD KADIWSLGITAIELAKGEPPNSDLHPMRVLFLIPKN
Sbjct  95   QSAYDFKADIWSLGITAIELAKGEPPNSDLHPMRVLFLIPKN  136
```

Protein alignment of Ir18

fibrillin-2 isoform X2 [Ixodes scapularis]

Sequence ID: XP_029839014.1 Length: 2880 Number of Matches: 28

Range 1: 2490 to 2756 GenPept Graphics ▼ Next Match ▲ Previous Match

Score	Expect	Method	Identities	Positives	Gaps	Frame
491 bits(1265)	7e-159	Compositional matrix adjust.	256/267(96%)	260/267(97%)	0/267(0%)	+1

```
Query  148    KFPVSPCKSKCLNTEGSYICSCDDGYVLAEDKVSCKDVDECATDRHNCEHSCINTQGSYR  327
              + PVSPCKSKCLNTEGSYICSCDDGYVLAEDKVSCKDVDECATDRHNCEHSCINTQGSYR
Sbjct  2490   ELPVSPCKSKCLNTEGSYICSCDDGYVLAEDKVSCKDVDECATDRHNCEHSCINTQGSYR  2549

Query  328    CSCKDGYNVLESQCLDVNECREQPHLCAPSGTCTNVPGSYRCNCPRGYITDVSGQSCKDD  507
              CSCKDGYNVLESQCLDVNECREQPHLCAPSGTCTN+PGSYRCNCPRGYITD SGQSCKDD
Sbjct  2550   CSCKDGYNVLESQCLDVNECREQPHLCAPSGTCTNMPGSYRCNCPRGYITDASGQSCKDD  2609

Query  508    NECLSQPCGTANCVNTVGSYSCSCPNGFSFDEIMTSCVGGSGCSNSPCLFECMPTGSGGF  687
              NECLSQPCGTANCVNTVGSYSC+CPNGFSFDEIMTSCVGGSGCSNSPCLFECMPTGSGGF
Sbjct  2610   NECLSQPCGTANCVNTVGSYSCNCPNGFSFDEIMTSCVGGSGCSNSPCLFECMPTGSGGF  2669

Query  688    LCSCPEGYQRIGQGHCLSTHPESSTLgpgkgvpggvgIPTYDIDNAGPQDPDKVISTEGC  867
              LCSCPEGYQRIGQGHCLSTHPESSTLGPGKGVPG VGIP YD+ NAGPQDPDKVISTEGC
Sbjct  2670   LCSCPEGYQRIGQGHCLSTHPESSTLGPGKGVPGDVGIPAYDVGNAGPQDPDKVISTEGC  2729

Query  868    YSCKLNGGSVQRARRGLTNGTLSRRLT  948
              YSCKLNGGSVQRARRGL NGT SRRLT
Sbjct  2730   YSCKLNGGSVQRARRGLNNGTSSRRLT  2756
```

dumpy, isoform X [Drosophila melanogaster]

Sequence ID: NP_001260039.1 Length: 14825 Number of Matches: 19

See 1 more title(s) ⌄ See all Identical Proteins(IPG)

Range 1: 185 to 369 GenPept Graphics ▼ Next Match ▲ Previous Match

Score	Expect	Method	Identities	Positives	Gaps	Frame
119 bits(299)	2e-29	Compositional matrix adjust.	84/190(44%)	108/190(56%)	15/190(7%)	+1

```
Query  172   SKCLNTEGSYICSCDDGYVLAEDKVSCKDVDECATDRHNC--EHSCINTQGSYRCSCKDG  345
             ++C N    ++C C DGY   + +V C DVDEC  +  NC     C NT G+Y CSC DG
Sbjct  185   AECCNLPAHFLCKCKDGY-EGDGEVLCTDVDECR-NPENCGPNALCTNTPGNYTCSCPDG  242

Query  346   Y---NVLESQCLDVNECREQPHLCAPSGTCTNVPGSYRCNCPRGYITDVSGQS-CKDDNE  513
             Y   N     C DV+EC   P++C P   CTN+ GSYRC+CP GY  D   +S C D +E
Sbjct  243   YVGNNPYREGCQDVDEC-SYPNVCGPGAICTNLEGSYRCDCPPGYDGDGRSESGCVDQDE  301

Query  514   CLSQPCG-TANCVNTVGSYSCSCPNGFSFDEIMTSCVGGSGCS-NSPCLF--ECMPTGSG  681
             C   PCG  A+C+NT GS+ C CP+G+S D  M  C     C+ N+PC    EC+  G G
Sbjct  302   CARTPCGRNADCLNTDGSFRCLCPDGYSGDP-MNGCEDVDECATNNPCGLGAECVNLG-G  359

Query  682   GFLCSCPEGY  711
              F C CP G+
Sbjct  360   SFQCRCPSGF  369
```

fibrillin-1 [Aedes aegypti]

Sequence ID: XP_021695535.1 **Length:** 1425 **Number of Matches:** 11

Range 1: 587 to 792 GenPept Graphics ▼ Next Match ▲ Previous Match

Score	Expect	Method	Identities	Positives	Gaps	Frame
119 bits(299)	1e-29	Compositional matrix adjust.	72/207(35%)	106/207(51%)	8/207(3%)	+1

```
Query  166    CKSKCLNTEGSYICSCDDGYVLAEDKVSCKDVDECATDRHNCEHSCINTQGSYRCSCKDG  345
              C + C+N  G Y C+C+ G+ L +D+ +C+DVDEC+T  H+C H C+N  G++ C C  G
Sbjct  587    CSNICINLLGDYKCACEAGFELEDDQQTCRDVDECSTKIHDCSHICVNVPGTFECECPSG  646

Query  346    YNVLESQ--CLDVNECREQPHLCAPSGTCTNVPGSYRCNCPRGYITDVSGQSCKDDNECL  519
              + +   +  C D+NEC   P+       C N+PGS+RC C  G+  ++  ++C D +EC
Sbjct  647    FILGRDKFTCEDINECETLPNKADCEHHCINLPGSHRCGCEDGHRLELDNRTCSDIDECS  706

Query  520    SQPCG-TANCVNTVGSYSCSCPNGFSFDEIMTSCVGGSGCS----NSPCLFECMPTGSGG  684
               +    + +C NT GS+ CSCP+G   D    +C+    C     N  C   C     G
Sbjct  707    DELKRCSHDCFNTKGSFECSCPSGLRLDVDKVTCLDIDECKINNFNGGCSHIC-ENQHGS  765

Query  685    FLCSCPEGYQRIGQGHCLSTHPESSTL  765
              F C CPEG            H E S L
Sbjct  766    FKCLCPEGMNLGDDQATCEDHDECSIL  792
```

Range 2: 546 to 732 GenPept Graphics ▼ Next Match ▲ Previous Match ▲ First Match

Score	Expect	Method	Identities	Positives	Gaps	Frame
116 bits(290)	2e-28	Compositional matrix adjust.	71/190(37%)	102/190(53%)	10/190(5%)	+1

```
Query  166    CKSKCLNTEGSYICSCDDGYVLAEDKVSCKDVDECATDRHNCEHSCINTQGSYRCSCKDG  345
              C   C N  GSY CSC  G+ L  ++ +C+D+DEC  +  NC + CIN  G Y+C+C+ G
Sbjct  546    CSDTCTNLPGSYHCSCPVGFELGRNRHTCEDIDECIENNGNCSNICINLLGDYKCACEAG  605

Query  346    YNVLESQ--CLDVNECREQPHLCAPSGTCTNVPGSYRCNCPRGYITDVSGQSCKDDNECL  519
              + + + Q  C DV+EC  + H C  S  C NVPG++ C CP G+I      +C+D NEC
Sbjct  606    FELEDDQQTCRDVDECSTKIHDC--SHICVNVPGTFECECPSGFILGRDKFTCEDINECE  663

Query  520    SQPCGT---ANCVNTVGSYSCSCPNGFSFDEIMTSCVGGSGCSNS--PCLFECMPTGSGG  684
              + P       +C+N  GS+ C C +G   +    +C     CS+    C +C   T  G
Sbjct  664    TLPNKADCEHHCINLPGSHRCGCEDGHRLELDNRTCSDIDECSDELKRCSHDCFNT-KGS  722

Query  685    FLCSCPEGYQ  714
              F CSCP G +
```

Range 3: 625 to 818 GenPept Graphics ▼ Next Match ▲ Previous Match ▲ First Match

Score	Expect	Method	Identities	Positives	Gaps	Frame
107 bits(267)	2e-25	Compositional matrix adjust.	69/198(35%)	106/198(53%)	14/198(7%)	+1

```
Query  157    VSPCKSKCLNTEGSYICSCDDGYVLAEDKVSCKDVDECAT--DRHNCEHSCINTQGSYRC  330
              +  C   C+N  G++ C C  G++L  DK +C+D++EC T  ++ +CEH CIN  GS+RC
Sbjct  625    IHDCSHICVNVPGTFECECPSGFILGRDKFTCEDINECETLPNKADCEHHCINLPGSHRC  684

Query  331    SCKDGYNV-LESQ-CLDVNECREQPHLCAPSGTCTNVPGSYRCNCPRGYITDVSGQSCKD  504
               C+DG+ + L+++ C D++EC ++   C  S  C N  GS+ C+CP G   DV   +C D
Sbjct  685    GCEDGHRLELDNRTCSDIDECSDELKRC--SHDCFNTKGSFECSCPSGLRLDVDKVTCLD  742

Query  505    DNEC----LSQPCGTANCVNTVGSYSCSCPNGFSFDEIMTSCVGGSGCS--NSPCLFECM  666
               +EC     +  C    C N  GS+ C CP G +  +   +C     CS  N  C   C+
Sbjct  743    IDECKINNFNGGCSHI-CENQHGSFKCLCPEGMNLGDDQATCEDHDECSILNGGCSQRCV  801

Query  667    PTGSGGFLCSCPEGYQRI  720
                  GG+ C C  G+  +
Sbjct  802    -NFLGGYRCECESGFALM  818
```

Range 4: 422 to 647 GenPept Graphics ▼ Next Match ▲ Previous Match ▲ First Match

Score	Expect	Method	Identities	Positives	Gaps	Frame
106 bits(265)	4e-25	Compositional matrix adjust.	74/229(32%)	104/229(45%)	50/229(21%)	+1

```
Query  166    CKSKCLNTEGSYICSCDDGYVLAEDKVSCKDVDECATDRHNCEHSCINTQGSYRCSCKDG  345
              C   C N+EGS+ CSC  GY L+E+  SC D++EC     +C H CIN  GS++CSC  G
Sbjct  422    CDHACHNSEGSFHCSCHSGYRLSENGHSCLDINECEKSNGHCSHICINLLGSHQCSCPKG  481

Query  346    YNVLE--------------------------------------------SQCLDVNECRE  393
                ++E                                              C+DVNEC
Sbjct  482    LYLMEDGKTCDFVDECELNNGGCSHGCHYEHGVVSCTCPKGFELERNNFKTCIDVNECEH  541

Query  394    QPHLCAPSGTCTNVPGSYRCNCPRGYITDVSGQSCKDDNECLSQPCGTAN-CVNTVGSYS  570
              +   C  S TCTN+PGSY C+CP G+    +  +C+D +EC+      +N C+N +G Y
Sbjct  542    RNGGC--SDTCTNLPGSYHCSCPVGFELGRNRHTCEDIDECIENNGNCSNICINLLGDYK  599

Query  571    CSCPNGFSFDEIMTSCVGGSGCSNS--PCLFECMPTGSGGFLCSCPEGY  711
              C+C  GF  ++   +C     CS     C   C+    G F C CP G+
Sbjct  600    CACEAGFELEDDQQTCRDVDECSTKIHDCSHICVNV-PGTFECECPSGF  647
```

Range 5: 345 to 524 GenPept Graphics ▼ Next Match ▲ Previous Match First Match

Score	Expect	Method	Identities	Positives	Gaps	Frame
92.4 bits(228)	2e-20	Compositional matrix adjust.	62/188(33%)	95/188(50%)	19/188(10%)	+1

```
Query  184  NTEGSYICSCDDGYVLAEDKVSCKDVDECATDRHNCEHSCINTQGSYRCSCKDGYNVL--  357
            N +   +CSC  G+ L  D+ +C D++EC  D   CE +C+N +G ++CSC +G+
Sbjct  345  NPDSETLCSCPKGFKLDSDQRTCHDINECHEDNGGCEQNCLNFEGGHKCSCYEGFEPSPD  404

Query  358  -ESQCLDVNECREQPHLCAPSGTCTNVPGSYRCNCPRGYITDVSGQSCKDDNECLSQPCG  534
                C+D++EC      C     C N  GS+ C+C  GY    +G SC D NEC
Sbjct  405  DNRSCVDIDECSMSQGGC--DHACHNSEGSFHCSCHSGYRLSENGHSCLDINECEKSNGH  462

Query  535  TAN-CVNTVGSYSCSCPNGFSFDEIMTSC-------VGGSGCSNSPCLFECMPTGSGGFL  690
             ++ C+N +GS+ CSCP G    E   +C       +   GCS+  C +E      G
Sbjct  463  CSHICINLLGSHQCSCPKGLYLMEDGKTCDFVDECELNNGGCSHG-CHYE-----HGVVS  516

Query  691  CSCPEGYQ  714
            C+CP+G++
Sbjct  517  CTCPKGFE  524
```

Range 6: 796 to 951 GenPept Graphics ▼ Next Match ▲ Previous Match First Match

Score	Expect	Method	Identities	Positives	Gaps	Frame
83.2 bits(204)	3e-17	Compositional matrix adjust.	55/158(35%)	78/158(49%)	8/158(5%)	+1

```
Query  166  CKSKCLNTEGSYICSCDDGYVLAEDKVSCKDVDECATDRHNCEHSCINTQGSYRCSCKDG  345
            C  +C+N  G Y C C+ G+ L  D  +C+  + CA     C+H C    G   CSC++G
Sbjct  796  CSQRCVNFLGGYRCECESGFALMNDNKTCEVSNPCALRNGGCQHFCNLHNGVPSCSCREG  855

Query  346  YNVLESQ---CLDVNECREQPHLCAPSGTCTNVPGSYRCNCPRGYITDVSGQSCKDDNEC  516
            + V +S    C+D++EC + P+       C N  GSY C C  G+  +  GQ C D NEC
Sbjct  856  FMVSKSNSASCVDIDEC-QTPNDNNCQQKCVNTEGSYTCECFAGFEKNDLGQ-CIDVNEC  913

Query  517  LSQPCG---TANCVNTVGSYSCSCPNGFSFDEIMTSCV  621
            L    G    A C+N  GS+ C CP G    +   +C
Sbjct  914  LENNGGCGNKAKCINLAGSHRCVCPTGHKMGKDRKTCF  951
```

Range 7: 185 to 359 GenPept Graphics ▼ Next Match ▲ Previous Match First Match

Score	Expect	Method	Identities	Positives	Gaps	Frame
77.8 bits(190)	2e-15	Compositional matrix adjust.	65/181(36%)	85/181(46%)	13/181(7%)	+1

```
Query  193  GSYICSCDDGYVLAEDKVSCKDVDECATDRHNCEHSCINTQGSYRCSCKDGYNVL--ESQ  366
            G Y C C  GY   +   SC D+DEC   RH C H+C NT G Y+CSC  G  +
Sbjct  185  GDYRCECPLGY--QQHDHSCHDIDECELGRHLCSHNCHNTAGDYQCSCPKGLKLSTDSKT  242

Query  367  CLDVNECREQPH--LCAPSGTCTNVPGSYRCNCPRGYITDVSGQSCKDDNECLSQPCGTA  540
            C D++EC EQ +  LC     C N  GSYRC CP G   D  G  C+  + C +   G +
Sbjct  243  CDDIDECDEQDNEDLCG-DLNCLNTYGSYRCVCPEGKELDEYG-ICRQMDLCTTNNGGCS  300

Query  541  N-CVNTVGSYSCSCPNGFSFDEIMTSCVGGSGC--SNSPCLFECMPTGSGGFLCSCPEGY  711
            + C        C CP+     E   +C   + C  +N  C   C P      LCSCP+G+
Sbjct  301  HICTFFNRETFCDCPDDMELAEDGKTCTTINDCNLNNGGCSHICNPDSET--LCSCPKGF  358

Query  712  Q  714
            +
Sbjct  359  K  359
```

Range 8: 215 to 400 GenPept Graphics ▼ Next Match ▲ Previous Match First Match

Score	Expect	Method	Identities	Positives	Gaps	Frame
72.8 bits(177)	1e-13	Compositional matrix adjust.	64/191(34%)	85/191(44%)	13/191(6%)	+1

```
Query  166  CKSKCLNTEGSYICSCDDGYVLAEDKVSCKDVDECATDRHNCEH-----SCINTQGSYRC  330
            C   C NT G Y CSC  G  L+ D  +C D+DEC  D  + E      +C+NT GSYRC
Sbjct  215  CSHNCHNTAGDYQCSCPKGLKLSTDSKTCDDIDEC--DEQDNEDLCGDLNCLNTYGSYRC  272

Query  331  SCKDGYNVLE-SQCLDVNECREQPHLCAPSGTCTNVPGSYRCNCPRGYITDVSGQSCKDD  507
             C +G  + E   C  ++ C      C  S  CT       C+CP        G++C
Sbjct  273  VCPEGKELDEYGICRQMDLCTTNNGGC--SHICTFFNRETFCDCPDDMELAEDGKTCTTI  330

Query  508  NECLSQPCGTANCVNTVGSYSCSCPNGFSFDEIMTSCVGGSGCS--NSPCLFECMPTGSG  681
            N+C     G ++  N      CSCP GF  D    +C   + C   N  C   C+    G
Sbjct  331  NDCNLNNGGCSHICNPDSETLCSCPKGFKLDSDQRTCHDINECHEDNGGCEQNCL-NFEG  389

Query  682  GFLCSCPEGYQ  714
            G  CSC EG++
Sbjct  390  GHKCSCYEGFE  400
```

Range 9: 34 to 286 GenPept Graphics ▼ Next Match ▲ Previous Match ⩓ First Match

Score	Expect	Method	Identities	Positives	Gaps	Frame
67.8 bits(164)	4e-12	Compositional matrix adjust.	72/257(28%)	92/257(35%)	75/257(29%)	+1

```
Query  178   CLNTEGSYICSCDDGYVLAEDKVSCKDVDECATDRHN---------------CEHSCINT  312
             C N  G Y C+C   GY LA+D   SC+D++EC                   C H C+N
Sbjct  34    CRNAYGGYECTCPAGYELADDDRSCQDINECEAYGQQEEDDYEDEAGHGVGICSHECVNA  93

Query  313   QGSYRCSCKDGYNV-------LESQCLDVNE-----------------------------  384
              GS+ C C D +++       +   C D+ E
Sbjct  94    VGSFECRCPDNFHLNHDRRTCVRDFCADLYENPNKTRCSHECKDGTEGFVCSCPEFYILD  153

Query  385   ------CR-------EQPHLCAPSGTCTNVP-GSYRCNCPRGYITDVSGQSCKDDNEC-L  519
                   CR       E    C P G C  +  G YRC CP GY       SC D +EC L
Sbjct  154   EYDQKTCRNVYSCGEEYKKRCNP-GVCRMIAGGDYRCECPLGY--QQHDHSCHDIDECEL  210

Query  520   SQPCGTANCVNTVGSYSCSCPNGFSFDEIMTSCVGGSGCSNSPCL-----FECMPTGSGG  684
              +   + NC NT G Y CSCP G        +C     C             C+ T  G
Sbjct  211   GRHLCSHNCHNTAGDYQCSCPKGLKLSTDSKTCDDIDECDEQDNEDLCGDLNCLNT-YGS  269

Query  685   FLCSCPEGYQRIGQGHC  735
             + C CPEG +    G C
Sbjct  270   YRCVCPEGKELDEYGIC  286
```

Range 10: 17 to 197 GenPept Graphics ▼ Next Match ▲ Previous Match ⩓ First Match

Score	Expect	Method	Identities	Positives	Gaps	Frame
56.2 bits(134)	2e-08	Compositional matrix adjust.	52/184(28%)	75/184(40%)	30/184(16%)	+1

```
Query  247   SCKDVDECATDRHNCEHSCINTQGSYRCSCKDGYNVLESQ--CLDVNECR----------  390
              C+D+DEC    H C++ C N  G Y C+C  GY + +    C D+NEC
Sbjct  17    ECEDIDECVETEHGCDY-CRNAYGGYECTCPAGYELADDDRSCQDINECEAYGQQEEDDY  75

Query  391   --EQPH-LCAPSGTCTNVPGSYRCNCPRGYITDVSGQSCKDD--NECLSQPCGT---ANC  546
               E  H +   S  C N  GS+ C CP  +  +   ++C  D   +    P  T     C
Sbjct  76    EDEAGHGVGICSHECVNAVGSFECRCPDNFHLNHDRRTCVRDFCADLYENPNKTRCSHEC  135

Query  547   VNTVGSYSCSCPNGFSFDEI-MTSCVGGSGCSN------SPCLFECMPTGSGGFLCSCPE  705
              +    + CSCP  +  DE    +C     C        +P +  C     G + C CP
Sbjct  136   KDGTEGFVCSCPEFYILDEYDQKTCRNVYSCGEEYKKRCNPGV--CRMIAGGDYRCECPL  193

Query  706   GYQR  717
             GYQ+
Sbjct  194   GYQQ  197
```

Range 11: 5 to 106 GenPept Graphics ▼ Next Match ▲ Previous Match ⩓ First Match

Score	Expect	Method	Identities	Positives	Gaps	Frame
33.9 bits(76)	0.26	Compositional matrix adjust.	27/104(26%)	40/104(38%)	19/104(18%)	+1

```
Query  454   NCPRGYITDVSGQSCKDDNECLSQPCGTANCVNTVGSYSCSCPNGFSFDEIMTSCVGGSG  633
             +C  G   +  G+ C+D +EC+    G   C N  G Y C+CP G+   +   SC   +
Sbjct  5     HCREGLRLNSHGE-CEDIDECVETEHGCDYCRNAYGGYECTCPAGYELADDDRSCQDINE  63

Query  634   CSN-----------------SPCLFECMPTGSGGFLCSCPEGYQ  714
             C                     C  EC+    G F C CP+ +
Sbjct  64    CEAYGQQEEDDYEDEAGHGVGICSHECV-NAVGSFECRCPDNFH  106
```

fibrillin, partial [Homo sapiens]

Sequence ID: CAA45118.1 **Length:** 3002 **Number of Matches:** 27

Range 1: 2579 to 2867 GenPept Graphics ▼ Next Match ▲ Previous Match

Score	Expect	Method	Identities	Positives	Gaps	Frame
177 bits(449)	1e-48	Compositional matrix adjust.	116/312(37%)	148/312(47%)	72/312(23%)	+1

```
Query  142   CTKFPVSPCKSKCLNTEGSYICSCDDGYVLAEDKVSCKDVDECATDRHNCEHSCINTQGS  321
             C + P  PC   C NTEGSY CSC  GY+L ED  SCKD+DECAT +HNC+  C+NT G
Sbjct  2579  CNQAP-KPCNFICKNTEGSYQCSCPKGYILQEDGRSCKDLDECATKQHNCQFLCVNTIGG  2637

Query  322   YRCSCKDGYNVLESQCLDVNECREQPHLCAPSGTCTNVPGS-------------------  444
             + C C  G+    + C+D NEC    +LC   G C N PGS
Sbjct  2638  FTCKCPPGFTQHHTSCIDNNECTSDINLCGSKGICQNTPGSFTCECQRGFSLDQTGSSCE  2697

Query  445   ---------------------YRCNCPRGYITDVSGQSCKDDNECLS-QPCGTANCVNTV  558
                                  YRC+CP+GY+       C D+NECLS   CG A+C NT+
Sbjct  2698  DVDECEGNHRCQHGCQNIIGGYRCSCPQGYLQHYQWNQCVDENECLSAHICGGASCHNTL  2757

Query  559   GSYSCSCPNGFSFDEIMTSC--VGGSGCSNSPCLFECMPTGSGGFLCSCPEGYQRIGQGH  732
             GSY C CP GF +++    C  +   G +  +PC + C  T  GG+LC CP GY RIGQGH
Sbjct  2758  GSYKCMCPAGFQYEQFSGGCQDINECGSAQAPCSYGCSNT-EGGYLCGCPPGYFRIGQGH  2816

Query  733   CLSTHPESSTLgpgkgvpggvgIPTYDIDNAGPQDP------DKVISTEGCYSCKLNGGS  894
             C+S                        +    P+ P      D  +S E CY CK+NG
Sbjct  2817  CVS---------------------GMGMGRGNPEPPVSGEMDDNSLSPEACYECKINGYP  2855

Query  895   VQRARRGLTNGT  930
              +  +R  TN T
Sbjct  2856  KRGRKRRSTNET  2867
```

Protein alignment of Ir19

TNF receptor-associated factor 4 isoform X2 [Ixodes scapularis]

Sequence ID: XP_029836788.1 **Length:** 386 **Number of Matches:** 2

Range 1: 9 to 249 GenPept Graphics ▼ Next Match ▲ Previous Match

Score	Expect	Method	Identities	Positives	Gaps	Frame
166 bits(419)	1e-47	Compositional matrix adjust.	107/294(36%)	144/294(48%)	53/294(18%)	+1

```
Query  133    QFNIPYHEVKGFTGLGVDFLRFPCKVPDSARCKHCSCIPKRICHLLCPQHGLCLDCKNDY  312
              +FNIPY +VKGF GLGVDFLRFPC+VPD A+CK C CIP+RI  LLCP HGLC DCK  +
Sbjct  9      KFNIPYCKVKGFIGLGVDFLRFPCQVPDRAKCKICDCIPERIYTLLCPHHGLCSDCKEFF  68

Query  313    QGYYCNVCHINTTPEQLGALRSDYNLDGLRLEVLCNVCDeelllgqleehlYKHTNQGHK  492
               G+YC  C I+TTPEQLGALRSDYNLDGLRL+VLC +CD++LLL
Sbjct  69     IGFYCRECDIHTTPEQLGALRSDYNLDGLRLKVLCPMCDKKLLL----------------  112

Query  493    EASKVQETTNQHGDVAEKPLRVSYAVSARCHFCNENVKNSDYVEHVMMHVKQRSNQGDSI  672
                          G++ E        +  +C  C   V N +   H  +  ++R     S
Sbjct  113    ------------GELEEHNRSECPKIVKKCSDCGVKVLNENLQSHQEVECQKRVVLCGSC  160

Query  673    VQETANEDTAMEVQETESDETRPHTDNKEHHLNREHHKNCDDDAVTCSRCGLFVQRNYMS  852
                  + +   +                        H + C +  V C  C   V R   S
Sbjct  161    KSAMSRDKLGV------------------------HDQVCPEKPVVCDDCKGEVLRKDES  196

Query  853    QHTLLTCPKRTIYCKLCYSSFPCLTEETHKYQCPKMERECSDCGVNVLNENLKN  1014
              +H    CP R + C+ C  S+   +E+ H+ QC   +  C  C + +  ++ K
Sbjct  197    KHA-SECPMRVVCCENCEGSYAYSSEKEHRKQCEIKKLACEYCKLYLKGDDEKK  249
```

Range 2: 116 to 147 GenPept Graphics ▼ Next Match ▲ Previous Match ⩓ First Match

Score	Expect	Method	Identities	Positives	Gaps	Frame
43.5 bits(101)	2e-04	Compositional matrix adjust.	19/32(59%)	27/32(84%)	0/32(0%)	+1

```
Query  928    EETHKYQCPKMERECSDCGVNVLNENLKNHLE  1023
              EE ++ +CPK+ ++CSDCGV VLNENL++H E
Sbjct  116    EEHNRSECPKIVKKCSDCGVKVLNENLQSHQE  147
```

transcription factor grauzone [Aedes aegypti]

Sequence ID: XP_001659339.1 **Length:** 640 **Number of Matches:** 1

See 1 more title(s) See all Identical Proteins(IPG)

Range 1: 424 to 521 GenPept Graphics ▼ Next Match ▲ Previous Match

Score	Expect	Method	Identities	Positives	Gaps	Frame
31.6 bits(70)	1.5	Compositional matrix adjust.	23/98(23%)	41/98(41%)	12/98(12%)	+1

```
Query  763    HLNREHHKNCDDDAVTCSRCGLFVQ-RNYMSQHTLLTCPKR-TIYCKLCYSSFPCLT---  927
              H++ ++H+   +    C+ C  F    + + QH  L   K     C +C   F  L+
Sbjct  424    HVHEKYHRKMSEKNFQCAHCDKFYAFESMLKQHERLVHTKECHFVCHICARGFQALSSYS  483

Query  928    -------EETHKYQCPKMERECSDCGVNVLNENLKNHL  1020
                     EE  K + P+   +CS C + V  +N + H+
Sbjct  484    SHLASHDEEAKKDKPPEERLQCSVCSIWVYKQNYRKHV  521
```

TNF receptor-associated factor 4 isoform X3 [Homo sapiens]

Sequence ID: XP_011523808.1 **Length:** 454 **Number of Matches:** 2

Range 1: 140 to 224 GenPept Graphics ▼ Next Match ▲ Previous Match

Score	Expect	Method	Identities	Positives	Gaps	Frame
57.0 bits(136)	7e-08	Compositional matrix adjust.	32/85(38%)	47/85(55%)	3/85(3%)	+1

```
Query  778    HHKNCDDDAVTC-SRCGLFVQRNYMSQHTLLTCPKRTIYCKLCYSSFPCLTEETHKYQCP  954
              H   C  ++V C ++CG  + R  ++QH    CPKRT  C  C   F   T ++H+YQCP
Sbjct  140    HEGMCPQESVYCENKCGARMMRRLLAQHATSECPKRTQPCTYCTKEFVFDTIQSHQYQCP  199

Query  955    KMERECSD-CGVN-VLNENLKNHLE  1023
              ++   C + CGV  V  E+L  HL+
Sbjct  200    RLPVACPNQCGVGTVAREDLPGHLK  224
```

Range 2: 86 to 167 GenPept Graphics ▼ Next Match ▲ Previous Match ⩓ First Match

Score	Expect	Method	Identities	Positives	Gaps	Frame
38.9 bits(89)	0.045	Compositional matrix adjust.	23/82(28%)	37/82(45%)	2/82(2%)	+1

```
Query  778    HHKNCDDDAVTC-SRCGLFVQRNYMSQHTLLTCPKRTIYCKLCYSSFPCLTEETHKYQCP  954
              H   C  + + C +RC + + R  +  H    CPKR + C+ C   F     E+H+  CP
Sbjct  86     HLNTCSFNVIPCPNRCPMKLSRRDLPAHLQHDCPKRRLKCEFCGCDFSGEAYESHEGMCP  145

Query  955    KMERECSD-CGVNVLNENLKNH  1017
              +    C + CG  ++   L  H
Sbjct  146    QESVYCENKCGARMMRRLLAQH  167
```

Protein alignment of Ir20

protein SGT1 homolog [Ixodes scapularis]

Sequence ID: XP_029842401.1 Length: 337 Number of Matches: 1

Range 1: 1 to 197 GenPept Graphics ▼ Next Match ▲ Previous Match

Score	Expect	Method	Identities	Positives	Gaps	Frame
384 bits(986)	7e-135	Compositional matrix adjust.	185/197(94%)	187/197(94%)	7/197(3%)	+2

```
Query  203  MAGSLETILSKANSAFVDENYTEALNLYNKALEESPSDAEIYVKRSHAHFRLGNWQASFD  382
            MA SLETILSKANSAFVDENYTEALNLYNKALEESPSDAEIYVKRSHAHFRLGNWQA+FD
Sbjct  1    MADSLETILSKANSAFVDENYTEALNLYNKALEESPSDAEIYVKRSHAHFRLGNWQATFD  60

Query  383  DLKAALMHGHQSAKAFLRMGISAFHLGKFQDAKDALEKGRALDSTETQFNEWLDKCGAQL  562
            DLKAALMHGHQSAKAFLRMG+SAFHLGKFQDAKDALEKGRALDSTETQF EWLDKCGAQL
Sbjct  61   DLKAALMHGHQSAKAFLRMGVSAFHLGKFQDAKDALEKGRALDSTETQFCEWLDKCGAQL  120

Query  563  KTSEDTKQRPAPVPPPSAQSRIRHEWYQTESHVTITILLKNQKAENIE-------IRFKA  721
            KTSEDTKQR APVPPPSAQSRIRHEWYQTESHVTITILLKNQKAENIE       IRFKA
Sbjct  121  KTSEDTKQRSAPVPPPSAQSRIRHEWYQTESHVTITILLKNQKAENIETSFTRDTIRFKA  180

Query  722  RLPSGDDYELFLEVAHP  772
            RLPSGDDYELFLEVAHP
Sbjct  181  RLPSGDDYELFLEVAHP  197
```

small glutamine-rich tetratricopeptide containing protein, isoform B [Drosophila melanogaster]

Sequence ID: NP_001246058.1 Length: 331 Number of Matches: 1

See 4 more title(s) ⌄ See all Identical Proteins(IPG)

Range 1: 117 to 214 GenPept Graphics ▼ Next Match ▲ Previous Match

Score	Expect	Method	Identities	Positives	Gaps	Frame
66.2 bits(160)	3e-12	Compositional matrix adjust.	32/98(33%)	56/98(57%)	0/98(0%)	+2

```
Query  218  ETILSKANSAFVDENYTEALNLYNKALEESPSDAEIYVKRSHAHFRLGNWQASFDDLKAA  397
            E+I ++ N    +  Y EAL  YN+A+   P +   Y  R+ AH RLG  + +  D K+A
Sbjct  117  ESIKNEGNRLMKENKYNEALLQYNRAIAFDPKNPIFYCNRAAAHIRLGENERAVTDCKSA  176

Query  398  LMHGHQSAKAFLRMGISAFHLGKFQDAKDALEKGRALD  511
            L++ +  +KA+ R+G++  ++G F+ A+ A  K   L+
Sbjct  177  LVYNNNYSKAYCRLGVAYSNMGNFEKAEQAYAKAIELE  214
```

small glutamine-rich tetratricopeptide repeat-containing protein beta [Aedes aegypti]

Sequence ID: XP_001657084.1 Length: 327 Number of Matches: 1

See 2 more title(s) ⌄ See all Identical Proteins(IPG)

Range 1: 86 to 177 GenPept Graphics ▼ Next Match ▲ Previous Match

Score	Expect	Method	Identities	Positives	Gaps	Frame
59.7 bits(143)	4e-10	Compositional matrix adjust.	30/92(33%)	54/92(58%)	0/92(0%)	+2

```
Query  218  ETILSKANSAFVDENYTEALNLYNKALEESPSDAEIYVKRSHAHFRLGNWQASFDDLKAA  397
            E + ++ N    +E Y EALN Y+KA+    ++   Y  R+ A+ RLG++QA+ DD + +
Sbjct  86   ENLKNEGNRLMKEEKYQEALNTYSKAISLDATNPVFYCNRAAAYSRLGDYQAAADDCRMS  145

Query  398  LMHGHQSAKAFLRMGISAFHLGKFQDAKDALE  493
            L +    +KA+ R+G++   + K + A DA +
Sbjct  146  LRYDPNYSKAYGRLGLAYSKMNKHEQALDAYQ  177
```

protein SGT1 homolog isoform A [Homo sapiens]

Sequence ID: NP_006695.1 Length: 333 Number of Matches: 1

See 3 more title(s) ⌄ See all Identical Proteins(IPG)

Range 1: 21 to 195 GenPept Graphics ▼ Next Match ▲ Previous Match

Score	Expect	Method	Identities	Positives	Gaps	Frame
121 bits(303)	2e-31	Compositional matrix adjust.	63/183(34%)	99/183(54%)	15/183(8%)	+2

```
Query  245  AFVDENYTEALNLYNKALEESPSDAEIYVKRSHAHFRLGNWQASFDDLKAALMHGHQSAK  424
            A +DE+   AL    KALE+ P DA+ Y +R++ H  LGN+  +  D K +L     ++
Sbjct  21   ALIDEDPQAALEELTKALEQKPDDAQYYCQRAYCHILLGNYCVAVADAKKSLELNPNNST  80

Query  425  AFLRMGISAFHLGKFQDAKDALEKGRALDSTETQFNEWLDKCGAQLKTSEDTKQRPAPVP  604
            A LR GI  +H   +  A +   +G+ LDS +  F+ W+ +C      SE
Sbjct  81   AMLRKGICEYHEKNYAAALETFTEGQKLDSADANFSVWIKRCQEAQNGSESEVW------  134

Query  605  PPSAQSRIRHEWYQTESHVTITILLKNQKAENIEIRFK-------ARLPSGDDYELFLEV  763
              + QS+I+++WYQTES V IT+++KN +  ++ + F         +LPSG+DY L LE+
Sbjct  135  --THQSKIKYDWYQTESQVVITLMIKNVQKNDVNVEFSEKELSALVKLPSGEDYNLKLEL  192

Query  764  AHP  772
             HP
Sbjct  193  LHP  195
```

Protein alignment of Ir21

E3 ubiquitin-protein ligase TRIM68-like [Ixodes scapularis]

Sequence ID: XP_029828056.1 **Length:** 508 **Number of Matches:** 5

Range 1: 203 to 417 GenPept Graphics ▼ Next Match ▲ Previous Match

Score	Expect	Method	Identities	Positives	Gaps	Frame
213 bits(542)	2e-65	Compositional matrix adjust.	119/215(55%)	146/215(67%)	0/215(0%)	+3

```
Query  132  DRDSWKLKAEDTNHTLESVRNDLGKKTADRDSWKFLAEHATRTLESVRNALRRTKADRDS  311
            +RD WKLKAED + TLES+ NDLGK  ADRDSWK  AE A+R LESVRN L +T ADRDS
Sbjct  203  ERDGWKLKAEDASRTLESLCNDLGKTMADRDSWKLKAEDASRMLESVRNDLGKTMADRDS  262

Query  312  WKLKAEDASRTLKRVRKDLGKKTADRDSWKLKAEDTNHTLESVRNDLGKTTADRDSWKLK  491
            WKLKAEDA R LK VR DL K TADRD W+LKA+D ++ ++  R+DL + TADRD+WK
Sbjct  263  WKLKAEDAIRMLKSVRNDLAKTTADRDCWELKAKDASNIVKIARDDLAQMTADRDNWKFT  322

Query  492  AEDTNHTLESVRNDLGKTTADRDSWKFLAEHATRTLESVRNDLGKTTADRDSWKRKAEDA  671
            A + N  LE +R+ L K  ADRDSWKF A   +R LES+R  L + TADRDSWK KA D+
Sbjct  323  AGNANLNLEGLRDVLAKAAADRDSWKFKAGDTSRKLESLRESLTQMTADRDSWKYKAGDS  382

Query  672  SNTVKSARNDLAQMKQDKDI*KVTAGVTNFNLDVL  776
               ++S    L +     D  K+ A   N  L++L
Sbjct  383  GRMLESLHQSLTEKTAYGDSWKLKADDANRKLEIL  417
```

Range 2: 160 to 393 GenPept Graphics ▼ Next Match ▲ Previous Match First Match

Score	Expect	Method	Identities	Positives	Gaps	Frame
206 bits(524)	9e-63	Compositional matrix adjust.	118/234(50%)	153/234(65%)	0/234(0%)	+3

```
Query  87   NQTSLYKKVGGRPTADRDSWKLKAEDTNHTLESVRNDLGKKTADRDSWKFLAEHATRTLE  266
            N + + K      TA  + W+ +A + +  ++ + N L +   +RD WK  AE A+RTLE
Sbjct  160  NHSCMLKLRLQETTASLEKWQQEASERSELVKCLDNTLAEIKLERDGWKLKAEDASRTLE  219

Query  267  SVRNALRRTKADRDSWKLKAEDASRTLKRVRKDLGKKTADRDSWKLKAEDTNHTLESVRN  446
            S+ N L +T ADRDSWKLKAEDASR L+ VR DLGK  ADRDSWKLKAED    L+SVRN
Sbjct  220  SLCNDLGKTMADRDSWKLKAEDASRMLESVRNDLGKTMADRDSWKLKAEDAIRMLKSVRN  279

Query  447  DLGKTTADRDSWKLKAEDTNHTLESVRNDLGKTTADRDSWKFLAEHATRTLESVRNDLGK  626
            DL KTTADRD W+LKA+D ++ ++  R+DL + TADRD+WKF A +A   LE +R+ L K
Sbjct  280  DLAKTTADRDCWELKAKDASNIVKIARDDLAQMTADRDNWKFTAGNANLNLEGLRDVLAK  339

Query  627  TTADRDSWKRKAEDASNTVKSARNDLAQMKQDKDI*KVTAGVTNFNLDVLLGAL  788
              ADRDSWK KA D S  ++S R  L QM  D+D  K  AG +   L+ L  +L
Sbjct  340  AAADRDSWKFKAGDTSRKLESLRESLTQMTADRDSWKYKAGDSGRMLESLHQSL  393
```

Range 3: 226 to 429 GenPept Graphics ▼ Next Match ▲ Previous Match First Match

Score	Expect	Method	Identities	Positives	Gaps	Frame
206 bits(523)	1e-62	Compositional matrix adjust.	113/204(55%)	141/204(69%)	0/204(0%)	+3

```
Query  117  GRPTADRDSWKLKAEDTNHTLESVRNDLGKKTADRDSWKFLAEHATRTLESVRNALRRTK  296
            G+  ADRDSWKLKAED +  LESVRNDLGK  ADRDSWK  AE A R L+SVRN L +T
Sbjct  226  GKTMADRDSWKLKAEDASRMLESVRNDLGKTMADRDSWKLKAEDAIRMLKSVRNDLAKTT  285

Query  297  ADRDSWKLKAEDASRTLKRVRKDLGKKTADRDSWKLKAEDTNHTLESVRNDLGKTTADRD  476
            ADRD W+LKA+DAS  +K  R DL + TADRD+WK  A + N  LE +R+ L K  ADRD
Sbjct  286  ADRDCWELKAKDASNIVKIARDDLAQMTADRDNWKFTAGNANLNLEGLRDVLAKAAADRD  345

Query  477  SWKLKAEDTNHTLESVRNDLGKTTADRDSWKFLAEHATRTLESVRNDLGKTTADRDSWKR  656
            SWK KA DT+  LES+R  L + TADRDSWK+ A  + R LES+   L + TA  DSWK
Sbjct  346  SWKFKAGDTSRKLESLRESLTQMTADRDSWKYKAGDSGRMLESLHQSLTEKTAYGDSWKL  405

Query  657  KAEDASNTVKSARNDLAQMKQDKD  728
            KA+DA+  ++  R  LA+  ++++
Sbjct  406  KADDANRKLEILRESLAETTENRE  429
```

Range 4: 254 to 495 GenPept Graphics ▼ Next Match ▲ Previous Match First Match

Score	Expect	Method	Identities	Positives	Gaps	Frame
176 bits(446)	3e-51	Compositional matrix adjust.	109/242(45%)	142/242(58%)	21/242(8%)	+3

```
Query  117  GRPTADRDSWKLKAEDTNHTLESVRNDLGKKTADRDSWKFLAEHATRTLESVRNALRRTK  296
            G+  ADRDSWKLKAED    L+SVRNDL K TADRD W+  A+ A+  ++  R+ L +
Sbjct  254  GKTMADRDSWKLKAEDAIRMLKSVRNDLAKTTADRDCWELKAKDASNIVKIARDDLAQMT  313

Query  297  ADRDSWKLKAEDASRTLKRVRKDLGKKTADRDSWKLKAEDTNHTLESVRNDLGKTTADRD  476
            ADRD+WK  A +A+  L+ +R  L K  ADRDSWK KA DT+  LES+R  L + TADRD
Sbjct  314  ADRDNWKFTAGNANLNLEGLRDVLAKAAADRDSWKFKAGDTSRKLESLRESLTQMTADRD  373

Query  477  SWKLKAEDTNHTLESVRNDLGKTTADRDSWKFLAEHATRTLESVRNDLGKT---------  629
            SWK KA D+   LES+   L + TA  DSWK  A+ A R LE +R  L +T
Sbjct  374  SWKYKAGDSGRMLESLHQSLTEKTAYGDSWKLKADDANRKLEILRESLAETTENRENWKR  433

Query  630  ------------TADRDSWKRKAEDASNTVKSARNDLAQMKQDKDI*KVTAGVTNFNLDV  773
                        TADRDSWKRKAEDA +T++S  + L +   D +  K +  + N    +
Sbjct  434  RALMAIRSQREMTADRDSWKRKAEDAKSTLESLPDSLPRTTADGESWKCSVLLENCIAVI  493

Query  774  LL  779
            L+
Sbjct  494  LV  495
```

Range 5: 400 to 420 GenPept Graphics ▼ Next Match ▲ Previous Match First Match

Score	Expect	Method	Identities	Positives	Gaps	Frame
42.4 bits(98)	4e-04	Compositional matrix adjust.	20/21(95%)	20/21(95%)	0/21(0%)	+1

```
Query  808  GDSWKLKAIDANRKLEILRES  870
            GDSWKLKA DANRKLEILRES
Sbjct  400  GDSWKLKADDANRKLEILRES  420
```

collagen alpha-2(IV) chain-like [Homo sapiens]

Sequence ID: XP_003959982.2 **Length:** 396 **Number of Matches:** 3

See 1 more title(s) See all Identical Proteins(IPG)

Range 1: 145 to 316 GenPept Graphics

▼ Next Match ▲ Previous Match

Score	Expect	Method	Identities	Positives	Gaps	Frame
44.7 bits(104)	2e-07	Compositional matrix adjust.	46/172(27%)	62/172(36%)	0/172(0%)	+2

```
Query  137  RQLEAQGGGHQPHAGKRTQRPWKEDGGQRQLEILSGACHPHAGKRTQRP*KDEGGQRQLE  316
            R L+ +G    P        PW+E G  R LE  +    P        P ++ G  R LE
Sbjct  145  RPLDRRGSVASPGEKGECGVPWREGGVWRPLERRASVASPGQKGECGVPWREGGVWRPLE  204

Query  317  AQGGGRQPHAEKSTQRPWKEDGGQRQLEAQGGGHQPHAGKRTQRPWKDDGGQRQLEAQGG  496
             +G    P  +     PW+E    R LE +     P        PW++    R LE +G
Sbjct  205  RRGSVASPGEKGECGVPWREGRVWRPLERRASVASPGQKGECGVPWREGPVWRPLERRGS  264

Query  497  GHQPHAGKRTQRPWKDDGGQRQLEILSGACHPHAGKRTQRPWKDDGGQRQLE  652
               P        PW + G  R LE  +    P        PW + G  R LE
Sbjct  265  VASPGEKGECGVPWTEGGVWRPLERRASVASPGEKGECGVPWTEGGVWRPLE  316
```

Range 2: 170 to 347 GenPept Graphics

▼ Next Match ▲ Previous Match ⩓ First Match

Score	Expect	Method	Identities	Positives	Gaps	Frame
41.2 bits(95)	3e-06	Compositional matrix adjust.	46/178(26%)	62/178(34%)	0/178(0%)	+2

```
Query  128  GGQRQLEAQGGGHQPHAGKRTQRPWKEDGGQRQLEILSGACHPHAGKRTQRP*KDEGGQR  307
            G  R LE +     P        PW+E G  R LE       P        P ++    R
Sbjct  170  GVWRPLERRASVASPGQKGECGVPWREGGVWRPLERRGSVASPGEKGECGVPWREGRVWR  229

Query  308  QLEAQGGGRQPHAEKSTQRPWKEDGGQRQLEAQGGGHQPHAGKRTQRPWKDDGGQRQLEA  487
             LE +     P  +     PW+E    R LE +G    P        PW + G  R LE
Sbjct  230  PLERRASVASPGQKGECGVPWREGPVWRPLERRGSVASPGEKGECGVPWTEGGVWRPLER  289

Query  488  QGGGHQPHAGKRTQRPWKDDGGQRQLEILSGACHPHAGKRTQRPWKDDGGQRQLEAQG  661
            +     P        PW + G  R LE  +    P        PW++ G  R L+ +G
Sbjct  290  RASVASPGEKGECGVPWTEGGVWRPLERRASVASPGEKGECGVPWREGGVWRPLDRRG  347
```

Range 3: 95 to 263 GenPept Graphics

▼ Next Match ▲ Previous Match ⩓ First Match

Score	Expect	Method	Identities	Positives	Gaps	Frame
38.5 bits(88)	2e-05	Compositional matrix adjust.	44/169(26%)	64/169(37%)	2/169(1%)	+2

```
Query  161  GHQPHAGKRTQ--RPWKEDGGQRQLEILSGACHPHAGKRTQRP*KDEGGQRQLEAQGGGR  334
            GH   AG++ +   PW E    R LE  +    P        P ++    R L+ +G
Sbjct  95   GHSEAAGEKGECGIPWTEGQVWRPLERRASVASPGEKGECGVPWREGRVWRPLDRRGSVA  154

Query  335  QPHAEKSTQRPWKEDGGQRQLEAQGGGHQPHAGKRTQRPWKDDGGQRQLEAQGGGHQPHA  514
             P  +     PW+E G  R LE +     P        PW++ G  R LE +G    P
Sbjct  155  SPGEKGECGVPWREGGVWRPLERRASVASPGQKGECGVPWREGGVWRPLERRGSVASPGE  214

Query  515  GKRTQRPWKDDGGQRQLEILSGACHPHAGKRTQRPWKDDGGQRQLEAQG  661
                  PW++    R LE  +    P        PW++    R LE +G
Sbjct  215  KGECGVPWREGRVWRPLERRASVASPGQKGECGVPWREGPVWRPLERRG  263
```

Protein alignment of Ir22

glutathione S-transferase, putative, partial [Ixodes scapularis]

Sequence ID: EEC11349.1 **Length:** 197 **Number of Matches:** 1

Range 1: 60 to 197 GenPept Graphics ▼ Next Match ▲ Previous Match

Score	Expect	Method	Identities	Positives	Gaps	Frame
275 bits(702)	7e-95	Compositional matrix adjust.	134/138(97%)	136/138(98%)	0/138(0%)	+2

```
Query  107  EEQQRRVDMVEMQAFDIIMYCVRVCYDPEYTDDKRRQFLTEVAPDKLKQFVTFLAKAGPF  286
            EEQQRRVDMVEMQAFD+IMYCVRVCYDPEYT+DKRRQFLTEVAPDKLKQFVTFLAKAGPF
Sbjct  60   EEQQRRVDMVEMQAFDVIMYCVRVCYDPEYTEDKRRQFLTEVAPDKLKQFVTFLAKAGPF  119

Query  287  VAGDKVTYADFILYEALQVVRAMGPSSFKNYPELEKYCARVAALPGLREYLASPRFKAWP  466
            VAGDKVTYADFILYEALQVVRAMGPSSFKNYPELEKYCARVAALPGLREYLASPRFKAWP
Sbjct  120  VAGDKVTYADFILYEALQVVRAMGPSSFKNYPELEKYCARVAALPGLREYLASPRFKAWP  179

Query  467  FWSPFAKALAVQHQPFTD  520
            FWSPFAKALAV HQP TD
Sbjct  180  FWSPFAKALAVPHQPPTD  197
```

glutathione S-transferase 1-1 [Aedes aegypti]

Sequence ID: XP_001654674.2 **Length:** 222 **Number of Matches:** 1

Range 1: 137 to 190 GenPept Graphics ▼ Next Match ▲ Previous Match

Score	Expect	Method	Identities	Positives	Gaps	Frame
35.8 bits(81)	0.016	Compositional matrix adjust.	15/54(28%)	29/54(53%)	0/54(0%)	+2

```
Query  251  QFVTFLAKAGPFVAGDKVTYADFILYEALQVVRAMGPSSFKNYPELEKYCARVA  412
            +F+        +VAGDK+T ADF +   +  ++      F  YP +E++ A+++
Sbjct  137  EFLEVYLGKTTYVAGDKLTVADFCILATITTMKVAAEIDFSKYPSIERWYAQLS  190
```

glutathione S-transferase Mu 1 isoform X1 [Homo sapiens]

Sequence ID: XP_005270839.1 **Length:** 184 **Number of Matches:** 1

Range 1: 57 to 179 GenPept Graphics ▼ Next Match ▲ Previous Match

Score	Expect	Method	Identities	Positives	Gaps	Frame
80.9 bits(198)	3e-22	Compositional matrix adjust.	44/126(35%)	70/126(55%)	3/126(2%)	+2

```
Query  107  EEQQRRVDMVEMQAFDIIMYCVRVCYDPEYTDDKRRQFLTEVAPDKLKQFVTFLAKAGPF  286
            EE++ RVD++E Q  D  M    +CY+PE+  +K +    E  P+KLK +  FL K  P+
Sbjct  57   EEEKIRVDILENQTMDNHMQLGMICYNPEF--EKLKPKYLEELPEKLKLYSEFLGKR-PW  113

Query  287  VAGDKVTYADFILYEALQVVRAMGPSSFKNYPELEKYCARVAALPGLREYLASPRFKAWP  466
             AG+K+T+ DF++Y+ L + R   P     +P L+ + +R   L  +  Y+ S RF   P
Sbjct  114  FAGNKITFVDFLVYDVLDLHRIFEPKCLDAFPNLKDFISRFEGLEKISAYMKSSRFLPRP  173

Query  467  FWSPFA  484
             +S  A
Sbjct  174  VFSKMA  179
```
