# Supplementary material for: Exploration of binary protein–protein interactions between tick-borne flaviviruses and Ixodes ricinus
Source: Parasit Vectors. 2021 Mar 6;14:144. doi: 10.1186/s13071-021-04651-3 (PMC7937244; doi:10.1186/s13071-021-04651-3)
Supplement: Supplementary file 7 — Additional file 7. Drosophila melanogaster orthologues of Ixodes ricinus genes and encoded proteins identified by yeast two-hybrid screening and gap repair. [file 13071_2021_4651_MOESM7_ESM.pdf]

***Drosophila melanogaster* orthologues of *Ixodes ricinus* genes and encoded proteins identified by yeast two-hybrid screening and gap repair**

I. ricinus **ID**	***D. melanogaster*** **gene stable ID**	***D. melanogaster*** **protein name and/or description**	**% identity of *I. ricinus* protein to *D. melanogaster***	**% query cover**	**E-value**
**Ir1**	FBgn0025366	Ip259: Intronic protein 259	78.12	95	3.00E-28
**Ir2**	FBgn0002525	Lam: Lamin	46.13	85	2.00E-80
**Ir3**	FBgn0001291	Jra: Djun gene product	35.9	55	3.00E-19
**Ir4**	-	-	-	-	-
**Ir5**	FBgn0011760	Ctp: Dynein light chain	96.39	39	4.00E-56
**Ir6**	-	-	-	-	-
**Ir7**	FBgn0031057	Ubqn: Ubiquilin	49.52	57	4.00E-21
**Ir8**	FBgn0032640	Sgt: Small glutamine-rich tetratricopeptide containing protein	31.47	62	1.00E-28
**Ir9**	FBgn0283649	Elgi: Early girl	24.02	62	2.00E-07
**Ir10**	FBgn0266465	GckIII: Germinal centre kinase III	93.12	69	8.00E-129
**Ir11**	FBgn0283649	TRAF6: TNF receptor-associated factor 6	25.36	40	6.20E-01
**Ir12**	FBgn0010355	TAF1: TBP-associated factor 1	80.38	85	1.00E-144
**Ir13**	FBgn0040348	CG3703: Uncharacterized protein Dmel	44.63	52	1.00E-16
**Ir14**	FBgn0034401	MetRS: Methionyl-tRNA synthetase	67.6	56	2.00E-81
**Ir15**	FBgn0261397	Didum: Myosin V	30.12	78	1.00E-08
**Ir16**	FBgn0036337	AdenoK: Adenosine kinase	46.24	62	3.00E-50
**Ir17**	FBgn0266465	GckIII: Germinal centre kinase III	95.1	99	4.00E-65
**Ir18**	FBgn0053196	Dpy: Dumpy	44.21	69	2.00E-29
**Ir19**	-	-	-	-	-
**Ir20**	FBgn0032640	Sgt: Small glutamine-rich tetratricopeptide containing protein	32.65	38	3.00E-12
**Ir21**	-	-	-	-	-
**Ir22**	-	-	-	-	-
