# Supplementary material for: Exploration of binary protein–protein interactions between tick-borne flaviviruses and Ixodes ricinus
Source: Parasit Vectors. 2021 Mar 6;14:144. doi: 10.1186/s13071-021-04651-3 (PMC7937244; doi:10.1186/s13071-021-04651-3)
Supplement: Supplementary file 8 — Additional file 8. Aedes aegypti orthologues of Ixodes ricinus genes and encoded proteins identified by yeast two-hybrid screening and gap repair. [file 13071_2021_4651_MOESM8_ESM.pdf]

***Aedes aegypti* orthologues of *Ixodes ricinus* genes and encoded proteins identified by yeast two-hybrid screening and gap repair.**

I. ricinus **ID**	***Ae. aegypti*** **gene stable ID**	***Ae. aegypti*** **protein name and/or description**	**% identity of *I. ricinus* protein to *Ae. aegypti***	**% query cover**	**E-value**
**Ir1**	AAEL013533	Ribosome biogenesis protein NSA2 homolog	75	95	2.00E-28
**Ir2**	AAEL003415	Lamin Dm0	41.26	86	1.00E-61
**Ir3**	AAEL003505	Transcription factor AP-1	40.59	56	2.00E-31
**Ir4**	AAEL006649	TNF receptor-associated factor 4	43.75	9	6.30E-01
**Ir5**	AAEL011478	Dynein light chain 1	91.86	41	4.00E-55
**Ir6**	-	-	-	-	-
**Ir7**	AAEL007160	Ubiquilin-1	63.81	36	7.00E-27
**Ir8**	AAEL003634	Small glutamine-rich tetratricopeptide repeat-containing protein beta	41.31	65	4.00E-51
**Ir9**	AAEL008184	E3 ubiquitin-protein ligase NRDP1	23.5	62	1.00E-06
**Ir10**	AAEL000589	Germinal center kinase 1	84.8	80	5.00E-132
**Ir11**	AAEL000730	E3 ubiquitin-protein ligase Bre1	31.48	18	7.00E-03
**Ir12**	AAEL025013	Transcription initiation factor TFIID subunit 1	80.38	85	7.00E-145
**Ir13**	AAEL000134	RUN domain-containing protein 1	55.56	52	2.00E-23
**Ir14**	AAEL014709	Methionin-trna ligase	65.36	56	3.00E-80
**Ir15**	AAEL024528	Unconventional myosin-Vb	29.67	68	2.00E-08
**Ir16**	AAEL001102	Adenosine kinase	50.51	64	2.00E-63
**Ir17**	AAEL000589	Serine/threonine-protein kinase 26	96.08	99	7.00E-67
**Ir18**	AAEL026163	Fibrillin-1	34.78	60	5.00E-30
**Ir19**	AAEL008551	Transcription factor grauzone	23.47	25	1.50E+00
**Ir20**	AAEL003634	Small glutamine-rich tetratricopeptide repeat-containing protein beta	32.61	35	4.00E-10
**Ir21**	-	-	-	-	-
**Ir22**	AAEL010582	Glutathione S-transferase 1-1	27.78	26	1.60E-02
