# Supplementary material for: Exploration of binary protein–protein interactions between tick-borne flaviviruses and Ixodes ricinus
Source: Parasit Vectors. 2021 Mar 6;14:144. doi: 10.1186/s13071-021-04651-3 (PMC7937244; doi:10.1186/s13071-021-04651-3)
Supplement: Supplementary file 9 — Additional file 9. Homo sapiens orthologues of Ixodes ricinus genes and encoded proteins identified by yeast two-hybrid screening and gap repair. [file 13071_2021_4651_MOESM9_ESM.pdf]

***Homo sapiens* orthologues of *Ixodes ricinus* genes and encoded proteins identified by yeast two-hybrid screening and gap repair.**

***I. ricinus* ID**	***H. sapiens* gene stable ID**	***H. sapiens* protein name and/or description**	**% identity of *I. ricinus* protein to *H. sapiens***	**% query cover**	**E-value**
**Ir1**	ENSG00000164346	NSA2: Ribosome biogenesis protein NSA2 homolog	80	97	2.00E-31
**Ir2**	ENSG00000113368	LMNB1: Lamin-B1	35.84	86	2.00E-51
**Ir3**	ENSG00000130522	JUND: JUN protein	81.13	25	6.00E-20
**Ir4**	ENSG00000175104	TRAF6: TNF Receptor-associated factor 6	26.09	14	4.70E+00
**Ir5**	ENSG00000264364	DYNLL2: Dynein light chain 2	95.18	39	3.00E-54
**Ir6**	-	-	-	-	-
**Ir7**	ENSG00000135018	UBQLN1: Ubiquilin-1	58.02	38	4.00E-26
**Ir8**	ENSG00000104969	SGTA: Small glutamine-rich tetratricopeptide repeat-containing protein alpha	48.95	73	1.00E-67
**Ir9**	ENSG00000179580	RNF151: RING finger protein 151	30.16	46	2.00E-09
**Ir10**	ENSG00000134602	STK26: Serine/threonine-protein kinase 26	86.18	69	7.00E-119
**Ir11**	ENSG00000082512	TRAF5: TNF receptor-associated factor 5	32.84	44	2.00E-11
**Ir12**	ENSG00000147133	TAF1: Transcription initiation factor TFIID subunit 1	83.78	84	1.00E-161
**Ir13**	ENSG00000198863	RUNDC1: RUN domain-containing protein 1	46.05	61	3.00E-25
**Ir14**	ENSG00000166986	MARS : Methionine-tRNA ligase	75.84	56	8.00E-95
**Ir15**	ENSG00000197535	MYO5A: Unconventional myosin-Va	41.46	55	4.00E-21
**Ir16**	ENSG00000156110	ADK: Adenosine kinase	45.03	64	9.00E-54
**Ir17**	ENSG00000115694	STK25 : Serine/threonine-protein kinase 25	93.14	99	2.00E-64
**Ir18**	ENSG00000138829	FBN: Fibrillin	37.18	87	1.00E-48
**Ir19**	ENSG00000076604	TRAF4: TNF receptor-associated factor 4	37.65	24	7.00E-08
**Ir20**	ENSG00000165416	SUGT1: Protein SGT1 homolog	34.43	68	2.00E-31
**Ir21**	ENSG00000134871	COL4A2: Collagen alpha-2(IV) chain-like	26.74	61	2.00E-07
**Ir22**	ENSG00000134184	GSTM1: Glutathione S-transferase Mu 1	34.92	62	3.00E-22
